# Metagenomic-Based Screening and Molecular Characterization of Cowpea-Infecting Viruses in Burkina Faso

**DOI:** 10.1371/journal.pone.0165188

**Published:** 2016-10-20

**Authors:** Essowè Palanga, Denis Filloux, Darren P. Martin, Emmanuel Fernandez, Daniel Gargani, Romain Ferdinand, Jean Zabré, Zakaria Bouda, James Bouma Neya, Mahamadou Sawadogo, Oumar Traore, Michel Peterschmitt, Philippe Roumagnac

**Affiliations:** 1 Laboratoire de Génétique et Biotechnologies Végétales, Université de Ouagadougou, 03 BP 7021, Ouagadougou, Burkina Faso; 2 Laboratoire de Virologie et de Biotechnologies Végétales, INERA, 01 BP 476, Ouagadougou, Burkina Faso; 3 CIRAD-INRA-SupAgro, UMR BGPI, F-34398, Montpellier, France; 4 LMI Patho-Bios, 01 BP 476, Ouagadougou, Burkina Faso; 5 Computational Biology Group, Institute of Infectious Disease and Molecular Medicine, Faculty of Health Sciences, University of Cape Town, Observatory, South Africa; University of Basel, SWITZERLAND

## Abstract

Cowpea, (*Vigna unguiculata* L. (Walp)) is an annual tropical grain legume. Often referred to as “poor man’s meat”, cowpea is one of the most important subsistence legumes cultivated in West Africa due to the high protein content of its seeds. However, African cowpea production can be seriously constrained by viral diseases that reduce yields. While twelve cowpea-infecting viruses have been reported from Africa, only three of these have so-far been reported from Burkina Faso. Here we use a virion-associated nucleic acids (VANA)-based metagenomics method to screen for the presence of cowpea viruses from plants collected from the three agro-climatic zones of Burkina Faso. Besides the three cowpea-infecting virus species which have previously been reported from Burkina Faso (Cowpea aphid borne mosaic virus [Family *Potyviridae*], the Blackeye cowpea mosaic virus—a strain of Bean common mosaic virus—[Family *Potyviridae*] and Cowpea mottle virus [Family *Tombusviridae*]) five additional viruses were identified: Southern cowpea mosaic virus (Sobemovirus genus), two previously uncharacterised polerovirus-like species (Family *Luteoviridae*), a previously uncharacterised tombusvirus-like species (Family *Tombusviridae*) and a previously uncharacterised mycotymovirus-like species (Family *Tymoviridae*). Overall, potyviruses were the most prevalent cowpea viruses (detected in 65.5% of samples) and the Southern Sudan zone of Burkina Faso was found to harbour the greatest degrees of viral diversity and viral prevalence. Partial genome sequences of the two novel polerovirus-like and tombusvirus-like species were determined and RT-PCR primers were designed for use in Burkina Faso to routinely detect all of these cowpea-associated viruses.

## Introduction

Cowpea, (*Vigna unguiculata* L. (Walp)), which is one of the most important subsistence legumes cultivated in West Africa [1] is an annual tropical grain legume that has seeds and leaves with a 25–30% protein content [2–4]. Cowpea is therefore one of the most important subsistance crops that are cultivated in West Africa.

Viral diseases, which can often occur as multiple infections, are a major constraint on cowpea production [5], and can cause plant stunting, reduced foliage, decreased seed protein content, and, in individual plants, yield losses of up to 93% [3, 6, 7]. While members of 140 virus species can naturally or artificially infect cowpea (reviewed in [8]), only twelve of these species have so far been found in Africa (Table 1), from which, only three have been reported from Burkina Faso. Viruses in eight of these twelve species are seedborne (Table 1): a factor that seriously hampers their effective control [9, 10]. For example, seed transmission can reach 2% for CMV, 6.9% for Blackeye cowpea mosaic virus—a strain of Bean common mosaic virus (BCMV-BlCM), and 13.3% for Cowpea aphid-borne mosaic virus (CABMV; [11]. Given that a far broader diversity of cowpea-infecting viruses has been discovered elsewhere in Africa, it is likely that additional cowpea-infecting viruses remain to be discovered in Burkina Faso. The limited available knowledge on cowpea infecting viruses in this country hinders the control of diseases, particularly with respect to the production of disease-free seeds and the creation of virus-resistant cowpea varieties. Hence, the main objective of this study was to further investigate the diversity of cowpea viruses in Burkina Faso.

**Table 1:** List of cowpea-infecting viruses present in Africa.

Virus	Country	Seedborne virus	Reference
Cowpea aphid-borne mosaic virus (CABMV)	Botswana, Burkina Faso, Cameroun, Ghana, Kenya, Mali, Morocco, Mozambique, Niger, Nigeria, Uganda, Senegal, South Africa, Togo, Zambia, Zimbabwe	Yes	[5, 9, 11, 12], this study
Blackeye cowpea mosaic virus—a strain of Bean common mosaic virus (BCMV-BlCMV)	Burkina Faso, Ghana, Togo, Nigeria, Kenya, Tanzania, Zambia	Yes	[5, 9, 11, 12], this study
Cowpea mottle virus (CPMoV)	Burkina Faso, Uganda, Senegal, Togo, Ivory coast	Yes	[5, 9, 13, 14], this study
Cowpea chlorotic mottle virus (CCMV)	Nigeria, Uganda	-	[5, 9]
Cucumber mosaic virus (CMV)	Benin, Cameroun, Ghana, Ivory coast, Uganda, Botswana, Mali, Niger, Nigeria, Kenya	Yes	[5, 9, 11]
Cowpea golden mosaic virus (CPGMV)	Nigeria	-	[5]
Cowpea mosaic virus (CPMV)	Uganda, Togo	Yes	[5, 9]
Cowpea severe mosaic virus (CPSMV)	Uganda, Senegal	Yes	[5, 9]
Cowpea yellow mosaic virus (CYMV)	Nigeria, Togo	-	[5, 9, 13]
Southern bean mosaic virus (SBMV)	Botswana, Ghana, Ivory coast, Kenya, Nigeria, Senegal, Togo	Yes	[5, 9, 15, 16]
Southern cowpea mosaic virus (SCPMV)	Burkina Faso, Botswana, Ghana, Ivory coast, Kenya, Nigeria	Yes	[5, 9, 16], this study
Sunn-hemp mosaic virus (SHMV)	Nigeria	-	[5]
Cowpea mild mottle virus (CPMMV)	Ivory coast, Uganda, Sudan, Tanzania, Togo	Yes	[5, 9]
Cowpea polerovirus 1	Burkina Faso		This study
Cowpea polerovirus 2	Burkina Faso		This study
Cowpea tombusvirid 1	Burkina Faso		This study
Cowpea tombusvirid 2	Burkina Faso		This study
Cowpea associated mycotymovirid 1	Burkina Faso		This study

The rapid advances in both nucleic acid sequencing technologies (next generation sequencing, NGS) and metagenomics-based approaches to study viromes at scales ranging from individual organisms to entire communities, have enabled the discovery of increasing numbers of viruses in both wild ecosystems and agro-ecosystems [17–21]. Metagenomics-based approaches have also provided estimation of the plant community prevalence of plant viruses at the agro-ecosystem scale [22, 23].

Here, we used a virion-associated nucleic acids (VANA) based metagenomics approach [24–27] to screen for the presence of cowpea viruses within cowpea plants collected from the Sudan (humid), Sudan-Sahel (sub-humid), and Sahel (dry) agro-climatic zones of Burkina Faso. Besides detecting four viruses that have so far been found infecting cowpea in Africa, we report the discovery of three novel plant virus species that have never before been found infecting cowpea plants, and one novel mycotymovirus, which probably infects a fungus species that is associated with cowpea plants.

## Materials and Methods

### Plant sampling

Three hundred and twelve leaf samples were randomly collected (i.e. irrespective of the presence of potential symptoms) in 2013 (S1 Table). 104 plants were sampled in the humid Sudan zone, 142 in the sub-humid Sudan-Sahel zone and 66 from the dry Sahel zone. The sampled plants were collected from 110 farmer’s fields or experimental plots. We confirm that owners of the cowpea fields gave permission to conduct the study on their sites. We confirm that the field studies did not involve endangered or protected species. Leaf samples were dried in the presence of CaCl2 and stored at 4°C until virion-associated nucleic acid extraction. Additionally, in 2014, 103 samples were collected in Burkina Faso, including 25 samples from the Sudan-Sahel zone and 78 from the Sudan zone (S1 Table).

### Detection of seed-borne viruses from cowpea seedlings

Eight cowpea cultivars (Komcallé, Nafi, Tiligré, Gorgou, Niizwé, Yiis-yandé, Kvx61-1, and Moussa local) obtained from Burkina Agricultural institute (INERA, Institut de l’Environnement et de Recherches Agricoles) and one unknown cultivar from Togo were grown at Montpellier, France within an insect-proof plant growth-chamber. Eighty-one seeds of each Burkina accession and twenty seeds of the Togo cultivar were sown in single use plastic pots containing sterilized peat and compost. Germinated seeds were examined daily during two weeks for the presence of symptoms on the primary and trifoliate leaves (S1 Table).

### Virion-associated nucleic acids extraction, cDNA amplification, sequencing and sequence analysis

The VANA-based 454 pyrosequencing approach [24] was used to analyse 384 cowpea plants, including 312 field plants sampled in Burkina Faso in 2013 and 72 plants grown in a growth-chamber at CIRAD (S1 Table). 150–250 mg of dried leaf material from the 384 plants were ground in Hanks’ buffered salt solution (HBSS) (1:10) with four ceramic beads (MP Biomedicals, USA) using a tissue homogeniser (MP biomedicals, USA). The homogenised plant extracts were centrifuged at 3,200 X g for 5 min and 6 ml of the supernatants were further centrifuged at 8,228 X g for 3 min. The resulting supernatants were then filtered through a 0.45 μm sterile syringe filter. The filtrate was then centrifuged at 148,000 X g for 2.5 hrs at 4°C to concentrate viral particles. The resulting pellet was resuspended overnight at 4°C in 200 μl of HBSS. Unencapsidated nucleic acids were eliminated by adding 15 U of bovine pancreas DNase I (Euromedex) and 1.9 U of bovine pancreas RNase A (Euromedex, France) followed by incubation at 37°C for 90 min. Total nucleic acids were finally extracted from 100μl of resuspended virions using a NucleoSpin 96 Virus Core Kit (Macherey-Nagel, Germany) following the manufacturer’s protocol. Viral cDNA synthesis was performed by incubation of 10 μl of extracted viral nucleic acids with 100 pmol of primer DoDec (5’-CCT TCG GAT CCT CCN NNN NNN NNN NN-3’) at 85°C for 2 min. The mixture was immediately placed on ice. Subsequently, 10 mM dithiothreitol, 1 mM of each deoxynucloside triphosphate (dNTP), 4 μl of 5X Superscript buffer, and 5 U of SuperScript III (Invitrogen, USA) were added to the mixture (final volume of 20 μl), which was then incubated at 25°C for 10 min, followed by 42°C incubation for 60 min and 70°C incubation for 5 min before being placed on ice for 2 min. cDNAs were purified using the QiaQuick PCR cleanup kit (Qiagen). Priming and extension was then performed using Large (Klenow) Fragment DNA polymerase (Promega). First, 20 μl of cDNA in the presence of 2 μM of primer DoDec were heated to 95°C for 2 min and then cooled to 4°C. 2.5 U of Klenow Fragment, 10X Klenow reaction buffer and 0.4 mM of each dNTP (final volume of 25 μl) were added. The mixture was incubated at 37°C for 60 min followed by 75°C for 10 min. PCR amplification was carried out using 5 μl of the reaction described above in a 20 μl reaction containing 2 μM of one of the 96 primers listed in S2 Table, and 10 μl of HotStarTaq Plus Master Mix Kit (Qiagen). The following cycling conditions were used: one cycle of 95°C for 5 min, five cycles of 95°C for 1 min, 50°C for 1 min, 72°C for 1.5 min, 35 cycles of 95°C for 30 sec, 50°C for 30 sec, 72°C for 1.5 min +2 sec at each cycle. An additional final extension for 10 min at 72°C was then performed. DNA products obtained from 96 cowpea samples were pooled, cleaned using the Wizard SV Gel and PCR Clean-Up System (Promega) and sequenced on 1/8th of a 454 pyrosequencing plate using GS FLX Titanium reagents (Beckman Coulter Cogenics, USA). The resulting reads were processed using a custom-built computational pipeline dedicated to the processing of multiplex identifier (MID) tagged DNA samples. Briefly, MID-tags and primers were identified in each raw read using agrep [28] in order to assign them to the particular samples from which they originated. Separated raw reads were then cleaned to eliminate MID-tags, primers and low quality regions (cut-off Phred quality score of 25) using cutadapt [29]. *De novo* assemblies of cleaned reads were performed using CAP3 [30]. Contigs and non-assembled reads with a minimum length of 45 bp were compared to sequences in the GenBank database using BlastN and BlastX methods [31]. Open reading frames (ORFs) were identified using the ORF Finder NCBI analysis tool (http://www.ncbi.nlm.nih.gov/gorf/gorf.html). Primary sequence outputs have been deposited in the sequence read archive of GenBank (accession number: SRP083221).

### Virus prevalence

The prevalence of a particular group of viruses was defined as the proportion of the 307 field sampled cowpea plants containing at least one VANA-read with a high degree of similarity (either BlastN or BlastX e-values <0.001) to that group of viruses. Five samples were considered to have failed because no VANA-reads were produced.

### RT-PCR, nested PCR and semi-nested PCR detection of viruses

A subset of fifty-two cowpea plants (S1 Table) that were initially processed by the VANA-based metagenomics approach was tested by RT-PCR to verify the presence of viruses identified during the metagenomic screen (S1 Table). This subset of plants included 20 plants within which one or more of these eight viruses were detected together with (i) twenty-seven plants that were collected within close proximity to these 20 plants and (ii) five seedlings grown at Montpellier in which potyvirus sequences were identified. In addition to these 52 plants, a further 103 cowpea plants collected in 2014 were tested by RT-PCR for the presence of the eight viruses.

Total RNA was extracted from 35–40 mg of CaCl_2_ dried cowpea leaves with the Qiagen^®^ RNeasy Plant Mini Kit (Qiagen, Valencia, CA) as described by the manufacturer. The detection of potyviruses was carried out using the primer pair Oligo1N/Oligo2N [32]. For the other viruses, contigs and reads produced in this study were aligned with related sequences obtained from GenBank (S3 Table) using ClustalW with default settings [33] and primers were designed (Table 2) using Oligo Explorer version 1.1.0 (www.uku.fi/~kuulasma/OligoSoftware) with customized settings (Tm, ~60°C; 40%<%GC<60%).

**Table 2:** List of detection primers designed in this study.

Cowpea viruses	Primers	Sequences	Gene	Annealing temperature (°C)	Extension duration (Sec)	Amplicon length (pb)
Cowpea polerovirus 1 and	PoleroNB3897F	GAGTTCATCTCCGAGGCC	*cp*	55	30	263
Cowpea polerovirus 2	PoleroNB4160R	CDTCTACCTATTTSGGRTTHTG				
SCPMV	SCPMVNB2698F	CTGGGARTTRTGGGCTGATG	*RdRp*	63	60	721
	SCPMVNB3419R	CTGAGCAATAGGGGCCATG				
	SCPMVNB2783F	TCRTGYTTCATGAACTCAGTC		53	30	133
	SCPMVNB2916R	AGYTCAGCCATRAGGCAWCG				
CPMoV	CPMoV1138F	TGAGYACTTTCATCAAAGCWGA	*RdRp*	53	60	548
	CPMoV1686R	ACACARTCRTCWCCGTTGTT				
	CPMoV1138F	TGAGYACTTTCATCAAAGCWGA		51	30	455
	CPMoV1593R	GTGTTCATRTCMCCACTCAT				
Cowpea tombusvirid 1	Tombus3NB31F	CAAGGTTCGACCAACATGTG	*RdRp*	57	30	412
	Tombus4NB79R	CCAGTTTACAACCTTGAGGAG				
	Tombus2NB237F	TGTCTCTCGTGCCGATGCT		55	30	308
	Tombus3NB52R	GGTTCGACCAACATGTGGG				
	Tombus2NB237F	TGTCTCTCGTGCCGATGCT	*RdRp/cp*	55	120	1772
	Tombus1NB44R	CCTGGTGTCGATGTGGCC				
	Tombus3NB31F	CAAGGTTCGACCAACATGTG		55	90	1485
	Tombus1NB44R	CCTGGTGTCGATGTGGCC				
Cowpea tombusvirid 2	Tomb2NB50F	CTGTGTGCTGTTCGTGGAG	*RdRp*	55	30	122
	Tomb2NB172R	TCAATCTTCTCTATATCGTAAAC				
Cowpea tombusvirid 3	Tomb1NB18F	TATCGGGGAGCGTTTGTACA	*RdRp*	55	30	175
	Tomb1NB193R	TGCATGTCGGGTGTAATACC				
Cowpea associated mycotymovirid 1	TymoNB120F	CTTTGGGTAGCACTATCCAC	*RP*	55	30	295
	TymoNB415R	GAGTTTTGCTCCTTGAGACG				
	TymoNB42F	GCTGCCATAGAAAAGCGCC	*RP*	55	30	154
	TymoNB196R	TAAAGAAGCTCGTCGAAGGG				

*cp*: coat protein; *RdRp*: *RNA dependant RNA polymerase; RP*: replication-associated polyprotein

RT-PCR reactions were performed using the Qiagen^®^ OneStep RT-PCR Kit. The 25 μL RT-PCR reaction mix consisted of 1 μL of eluted RNA (concentration range of 12–350 ng/μL), 14 μL of RNAse-free water, 5 μL of RT-PCR buffer (5X), 1 μL of dNTP mix (10 mM), 1.5 μL of each primer (10 μM) and 1 μL of RT-PCR enzyme mix. The RT-PCR program was as follows with the annealing temperature (Ta) and extension time (Ext) for each targeted virus listed in Table 2: 50°C for 30 min, 95°C for 15 min, 35 cycles at 94°C for 1 min, Ta for 1 min and 72°C for Ext with a final 72°C extension for 10 min. PCR products were analyzed by electrophoresis on a 1.2% agarose gel in TAE buffer stained with ethidium bromide and visualized under UV light.

Specific nested or semi-nested-PCR assays were also designed to improve the detection of Cowpea mottle virus (CPMoV), Southern cowpea mosaic virus (SCPMV), tombusvirus-like viruses and mycotymovirus. RT-PCRs were performed as described above using the following primers: CPMoV1138F/CPMoV1686R for CPMoV; SCPMVNB2698F /SCPMVNB3419R for SCPMV, Tombus2NB237F/Tombus4NB79R for Cowpea tombusvirid 1 and TymoNB120F/TymoNB415R for Cowpea associated mycotymovirid 1 (Table 2). PCR amplifications were carried out using 1 μL of the reaction volume described above in a 25 μL reaction mix containing 0.5 μl at 10 μM of each primer, 10.5 μL of RNAse-free water and 12.5 μL of the HotStarTaq Plus Master Mix Kit (Qiagen). The following cycling conditions were used: one cycle at 95°C for 5 min, 35 cycles at 94°C for 1 min, Ta (Table 2) for 1 min, Ext (Table 2) at 72°C. An additional final extension for 10 min at 72°C was then performed. Amplification products were sequenced using the Sanger method (Beckman Coulter Cogenics, USA).

### Recovery of partial genomes of Cowpea polerovirus 1 and Cowpea polerovirus 2

Twenty specific primers (S4 Table) were designed from the VANA-contigs assigned to Cowpea polerovirus 1. These primers were scattered along the VANA-contigs and were expected to amplify 1 Kb amplicons with 500 bp of sequence overlap between adjacent amplicons. In addition, two small products of 161 bp and 201 bp were amplified to confirm the 5’ end of the genome using primers PoleroNB1F/PoleroNB162R and PoleroNB1F/PoleroNB202R (S4 Table). Twelve specific primers were also designed, as described to amplify fragments of the Cowpea polerovirus 2 genome (S4 Table). RT-PCRs were performed as described above and amplicons were sequenced using the Sanger method (Beckman Coulter Cogenics, USA). Nucleotidic sequences were further assembled using DNAMAN v 7.0.2 (Lynnon Corporation).

### Cloning and sequencing of partial genome of Cowpea tombusvirid 1

VANA-contigs potentially coding RdRp and coat proteins of a novel virus hereafter referred to as Cowpea tombusvirid 1 were used to design primers for amplifying the genomic region encompassing these two positive sense single stranded RNA virus genes (Tombus2NB237F/Tombus1NB44R and Tombus3NB31F/Tombus1NB44R primer pairs; Table 2). RT-PCR was performed as described above using an annealing temperature of 55°C for the two primer combinations and an extension time of 2 min for Tombus2NB237F/Tombus1NB44R (1772 bp) and 1 min 30 sec for Tombus3NB31F/Tombus1NB44R (1485 bp). Amplified products were gel purified with the QIAquick Gel Extraction Kit (Promega), inserted into the pGEM^®^-T vector as recommended by the manufacturer (Promega) and sequenced by the Sanger method (Beckman Coulter Cogenics, USA) using the universal primers, T7 and SP6.

### GenBank accession numbers

Partial genome of Cowpea polerovirus 1 (KX599154), partial *RdRp* gene of Cowpea polerovirus 1 (KX599155-KX599163), partial genome of Cowpea polerovirus 2 (KX599164), partial *RdRp* gene of Cowpea mottle virus (KX599165-KX599169), partial genome of Southern cowpea mosaic virus (KX599170), partial *RdRp* gene of Southern cowpea mosaic virus (KX599171-KX599173), partial genome of Cowpea tombusvirid 1 (KX599174), partial *RdRp* gene of Cowpea tombusvirid 1 (KX599175-KX599177), partial *RdRp* gene of Cowpea tombusvirid 2 (KX599183), partial *RdRp* gene of Cowpea tombusvirid 3 (KX599184), partial *RP* gene of Cowpea associated mycotymovirid 1 (KX599178-KX599182).

### Phylogenetic analysis

Sanger sequences were assembled using DNAMAN and were used as queries to perform BlastN and BlastX searches [31]. Sequences were subsequently aligned using MUSCLE 3.7 with default settings [34]. Maximum likelihood phylogenetic trees were produced from this alignment using PhyML 3.1 [35] implemented in MEGA version 6.06 [36] with a K2+G+I (Polerovirus) and K2+G (Potyvirus, Carmovirus, Sobemovirus and *Tombusviridae*) nucleotidic substitution models (selected as best fit by MEGA) and 1000 bootstrap replicates as a test for the support of branches.

## Results and Discussion

### Exploration of cowpea virus diversity using the VANA-based metagenomics-approach

A total of 669,589 reads were obtained from the 384 cowpea samples that were processed using the VANA approach (S1 Table). No reads were obtained in five of the 312 field plants. The average read count for each plant sample was 2848 reads/plant (standard deviation: 3037 reads/plant). A total of 45,901 reads (6.85%) were discarded after the quality control process. BlastN and BlastX comparisons between the VANA-reads and GenBank sequences indicated that 20.89% of the processed reads were potentially related to plant RNA viruses and that among the 307 field plants in which reads were obtained, 203 were positive for the presence of virus-related reads (66.1%; S1 Table). Unexpectedly, no reads corresponding to plant DNA viruses were obtained. Five family-level plant viral lineages were identified, including the *Potyviridae*, *Luteoviridae*, *Tombusviridae* and *Tymoviridae* families and the unassigned Sobemovirus genus (Table 3).

**Table 3:** Selection of plant virus VANA-contigs and VANA-reads recovered from cowpea plants collected in Burkina Faso.

Plant sample	Agroclimatic zone/Province	Contig/read length (bp)	Number of reads in contig	Results from BlastX search				
				Virus/Accession number	Viral family/genus	Locus	Percent identity	e-value
BE57	Sahel/Yatenga	335	11	CABMV/CAA76872	*Potyviridae* / Potyvirus	polypeptide	88	6e-20
BE57		287	1	CABMV/AGK29853	*Potyviridae* / Potyvirus	CP	100	1e-51
BE57		5408	1818	CABMV/AEB34825	*Potyviridae* / Potyvirus	polyprotein (CP)	89.2	0.0
BE4	Sudan sahel/Kadiogo	1352	206	BCMV/CAC86161	*Potyviridae* / Potyvirus	unknown protein (polyprotein)	99	0.0
BE4		411	12	BCMV/NP_734117	*Potyviridae* / Potyvirus	CI protein	98.5	6e-72
BE4		245	1	BCMV/AGL95882	*Potyviridae* / Potyvirus	CP	95	6e-48
BE256	Sudan/Comoe	2239	257	CABMV/AIZ48757	*Potyviridae* / Potyvirus	polyprotein	88	0.0
BE256		1195	256	CABMV/AEB34826	*Potyviridae* / Potyvirus	polyprotein	84	0.0
BE256		298	1	CABMV/ADX94778	*Potyviridae* / Potyvirus	CP	96.8	1e-59
BE250	Sudan/Comoe	427	8	SCPMV/NP_042301	Sobemovirus	Polyprotein P2a	96	4e-21
BE250		646	157	SCPMV/NP_042300	Sobemovirus	putative MP	90	3e-111
BE250		3437	1070	SCPMV/AAA46565	Sobemovirus	CP	98	0.0
BE167	Sudan sahel/Gourma	1440	126	^a^BrYV/ADW41603	*Luteoviridae* / Polerovirus	P1-2 fusion protein	68	0.0
BE167		850	336	^b^BWYV/ADR74374	*Luteoviridae* / Polerovirus	P3-P5 readthrough protein domain	82	7e-139
BE167		769	8	^c^GRAV/AAG29927	*Luteoviridae* / Polerovirus	CP	77.8	7e-63
BE179	Sudan sahel/Gourma	313	1	^e^CpCSV/AEI55842	*Luteoviridae* / Polerovirus	P3-P5	72	4e-44
BE179		309	1	^d^PBMYV/ALO61879	*Luteoviridae* / Polerovirus	RdRp	89	8e-33
BE179		295	2	^f^CABYV/AEH27577	*Luteoviridae* / Polerovirus	CP	78	1e-42
BE120	Sudan sahel/Sanmatenga	254	1	^g^FgMTV1/AMN92730	*Tymoviridae* / Mycotymovirus	Replication associated polyprotein	60	7e-26
BE120		513	3	^g^FgMTV1/AMN92730	*Tymoviridae* / Mycotymovirus	Replication associated polyprotein	51	2e-22
BE273	Sudan/Poni	1504	772	CPMoV/AAC54603	*Tombusviridae* / Carmovirus	RNA replicase	97	0.0
BE273		578	289	CPMoV/NP_619521	*Tombusviridae* / Carmovirus	replicase RdRp	97	2e-124
BE273		498	25	CPMoV/NP_613271	*Tombusviridae* / Carmovirus	CP	65	4e-22
BE81	Sahel/Soum	487	6	^h^MCMV/AKQ24598	*Tombusviridae* / Machlomovirus	putative replicase	39	1e-23
BE81		334	6	^i^SgCV/NP_044384	*Tombusviridae* / Carmovirus	SCVP57	48	1e-26
BE81		304	1	^k^VTMV/AFN89806	Sobemovirus	CP	43	0.002
BE158	Sudan sahel/Oubritenga	204	1	^j^BBSV/CBA34987	*Tombusviridae* / Betanecrovirus	RdRp	47	3e-11
BE137	Sudan sahel/Sanmatenga	237	1	^h^MCMV/AMD02991	*Tombusviridae* / Machlomovirus	putative replicase	44	6e-04

^a^: Brassica yellows virus,

^b^: Beet western yellows virus,

^c^: Groundnut rosette assistor virus,

^d^: Phasey bean mild yellows virus,

^e^: Chickpea chlorotic stunt virus,

^f^: Cucurbit aphid borne yellow virus,

^g^: Fusarium graminearum mycotymovirus 1,

^h^: Maize chlorotic mottle virus,

^i^: Saguaro cactus virus,

^j^: Beet black scorch virus,

^k^: Velvet tobacco mottle virus

### Detection of known cowpea viruses

BlastX comparisons between the 3510 VANA-contigs that were produced by *de novo* assembly of potyvirus-, sobemovirus- and carmovirus-related reads and GenBank sequences yielded identity scores of 78–93% with CABMV, 98–100% with BCMV-BlCM, 90–96% with SCPMV and 65–97% with CPMoV (Table 3). These contigs apparently correspond with potyvirus genes (coat protein [*cp*], cytoplasmic inclusion protein [*ci*]), sobemovirus genes (polyprotein P2a, movement protein [*mp*] and *cp*), and carmovirus genes (RNA replicase, RNA dependent RNA polymerase [*RdRp*] and *cp*; Table 3). The degrees of similarity between these contigs and the amino acid (aa) or nucleotidic (nt) sequences of classified viruses in GenBank are above the species demarcation thresholds recommended for potyviruses (80% aa identity in the coat protein; [37]), carmoviruses (52% aa identity of the polymerase, 41% aa identity of the coat protein; [38]) and sobemoviruses (72% genome-wide pairwise nt sequence identity; [39]) indicating that the viral isolates from which these genomic sequences were obtained could reasonably, albeit tentatively, belong to the CABMV, BCMV-BlCM, SCPMV and CPMoV species. Of the 203 virus positive plants, 197 contained CABMV (97.04%), six contained BCMV-BlCM (2.96%), three contained SCPMV (1.48%) and three contained CPMoV (1.48%).

It is noteworthy that SCPMV is, to our knowledge, identified here for the first time in Burkina Faso. One of the three contigs is 3437 nt long (Table 3), which corresponds to slightly more than 80% of a typical SCPMV genome. Three large ORFs were identified within this contig: two overlapping ORFs corresponding to the P2a polyprotein encoding region (SCPMV, accession number NP_042301, highest percent identity = 96%, e-value = 0.0) and the P2ab polyprotein encoding region (SCPMV, accession number NP_042302, highest percent identity = 97%, e-value = 0.0) and an ORF3 corresponding to the CP protein encoding region (SCPMV, accession number ABW34399, highest percent identity = 98%, e-value = 0.0).

### Discovery of novel cowpea viruses

Reads and contigs showing high degrees of similarity with viruses in the families *Luteoviridae* and *Tymoviridae*—families with no previously known cowpea-infecting viruses—were identified from several cowpea plants collected during the 2013 sampling survey. In addition, reads and contigs showing low degrees of similarity with CPMoV, a member of the *Tombusviridae* family, were also identified.

Reads related to sequences of viruses in the family *Luteoviridae* were found in 10/203 (4.92%) of the evaluated plants (S1 Table). Eleven contigs were produced by *de novo* assembly of reads from two plants (BE167 and BE179; Table 3). These contigs apparently encoded partial CPs (two contigs), partial RdRps (two contigs) and partial P3-P5 readthrough proteins (two contigs, Table 3). Contigs obtained from both plants were further compared to one another. The pairwise identity scores that we obtained ranged from 57.9% (for the partial CP aa sequences) to 54.08% (for the partial P3-P5 aa sequences), suggesting that the reads may originate from two or more different luteovirus-like species. Further, a single 231 nt long read obtained from plant BE179 displayed a relatively high degree of similarity (highest percent identity = 79%, e-value = 8e-09) with a polerovirus *mp* gene (ORF4 of Pepo aphid-borne yellows virus, accession number CRL92752).

Reads and contigs showing low degrees of similarity with CPMoV, a virus in the family *Tombusviridae*, were also identified from 3/203 plants (Table 3). Two contigs, both sharing similarities with tombusvirus sequences were assembled from plant BE81 (Table 3). One of these contigs potentially encodes a sobemovirus-like coat protein that is most similar to that of Velvet tobacco mosaic virus (accession number AFN89806, identity = 35%, e-value = 4e-09). In addition, single reads that were most similar to tombusvirus-like *RdRp* genes (47–54%, Table 3), were recovered from two other plants (Table 3).

One 513 nt long contig and one 254 nt long single read showing detectable degrees of similarity with viruses in the family *Tymoviridae* were obtained from one plant (Table 3). Both of these sequence fragments may encode partial replication-associated polyproteins that are most similar to that of Fusarium graminearum mycotymovirus 1 (accession number AMN92730, BlastX highest percent identity = 51% and 60%, e-value = 2e-22 and = 7e-26, respectively). These results suggest that these fragments are likely derived from a cowpea-associated fungus, that potentially belongs to the recently proposed lineage mycotymovirus in the family *Tymoviridae* [40].

The seven putative plant viruses identified here using the VANA-based approach (two potyviruses, one sobemovirus, one carmovirus, two poleroviruses, and one tombusvirus-like virus) sometimes occurred in mixed infections (14/307 plants, 4.6%; S1 Table). While the co-infected cowpea plants mostly contained two detectable viruses (13/14), a single case of triple infection was also observed (S1 Table). There was no correlation between average read count and the occurrence of multiple virus infection.

### Molecular detection and characterisation of known and novel cowpea viruses

To validate the results of the metagenomic screen, RT-PCR detection assays using virus-specific primers (Table 2) were carried out on a subset of 52 samples collected in 2013 survey and the 103 cowpea plants collected in a further 2014 survey.

#### Potyviruses (CABMV and BCMV-BlCM)

Fourty out of the 52 plants collected in 2013 tested positive for potyviruses (S1 Table). Among these 40 samples, potyvirus-related VANA reads went undetected in only a single plant (BE121), suggesting that the potyvirus detection results obtained with both these molecular virus detection approaches were consistent. In addition, 94.1% of a subset of 17 plants collected in 2014 tested positive for potyviruses (S1 Table). Phylogenetic analysis based on the 182 nt partial nuclear inclusion gene indicated that these Burkinabe isolates all belong to either the CABMV or BCMV-BlCM species (S1 Fig).

#### Cowpea mottle virus (CPMoV)

**Fig 1:**
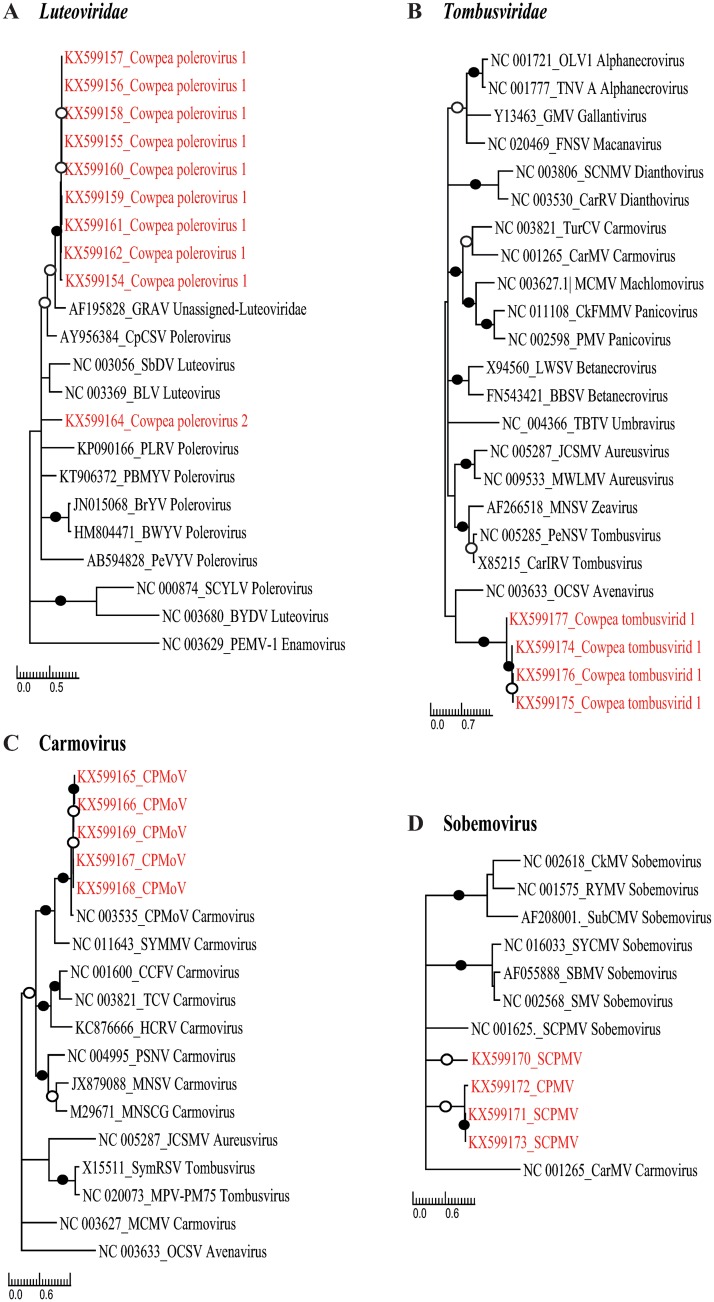
Maximum-likelihood phylogenetic trees depicting the relatedness of cowpea viruses from Burkina Faso. A) Maximum-likelihood phylogenetic trees of partial *cp* genes from nine isolates of Cowpea polerovirus 1 and representative species from the family *Luteoviridae*. SCYLV, Sugarcane yellow leaf virus; PLRV, Potato leafroll virus; PeVYV, Pepper vein yellows virus; CpCSV, Chickpea chlorotic stunt virus; BrYV, Brassica yellows virus; BWYV, Beet western yellows virus; BYDV, Barley yellow dwarf virus; BLRV, Bean leafroll virus; SbDV, Soybean dwarf virus; PEMV-1, Pea enation mosaic virus-1; GRAV, Groundnut rosette assistor virus; PBMYV, Phasey bean mild yellows virus. B) Maximum-likelihood phylogenetic trees of partial *RdRp* genes from four isolates of Cowpea tombusvirid-1 and representative species from the family *Tombusviridae*. TurCV, Turnip crinkle virus; MNSV, Melon necrotic spot virus; MCMV, Maize chlorotic mottle virus; JCSMV, Johnsongrass chlorotic stripe mosaic virus; OCSV, Oat chlorotic stunt virus; TNV A, Tobacco necrosis virus A; OLV1, Olive latent virus 1; PMV, Panicum mosaic virus; CMMV, Cocksfoot mild mosaic virus; CarMV, Carnation mottle virus; MWLMV, Maize white line mosaic virus; PNSV, Pelargonium necrotic spot virus; CIRV, Carnation Italian ringspot virus; GaMV, Galinsoga mosaic virus; FNSV, Furcraea necrotic streak virus; LWSV, Leek white stripe virus; BBSV, Beet black scorch virus; SCNMV, Sweet clover necrotic mosaic virus; CRSV, Carnation ringspot virus; CkMV, Cocksfoot mottle virus. C) Maximum-likelihood phylogenetic trees of partial *RdRp* gene from 5 isolates of CPMoV from Burkina Faso and representative species from Carmovirus genus and from the family *Tombusviridae*. CCFV, Cardamine chlorotic fleck virus; SYMMV, Soybean yellow mottle mosaic virus; HCRV, Hibiscus chlorotic ringspot virus; PSNV, Pea stem necrosis virus; MNSCG, Melon necrotic spot virus; CymRSV, Cymbidium ringspot tombusvirus; MPV-PM75, Moroccan pepper virus. D: Maximum-likelihood phylogenetic trees of partial *RdRp* genes from five isolates of SCPMV from Burkina Faso and representative species of the Sobemovirus genus. SCPMV, Southern cowpea mosaic virus; RYMV, Rice yellow Mottle virus; CfMV, Cocksfoot mottle virus_sobemovirus; SCMoV, Subterranean clover mottle virus; SYCMV, Soybean yellow common mosaic virus; SBMV, Southern bean mosaic virus; SeMV, Sesbania mosaic virus; CarMV, Carnation mottle virus. For all four trees, branches associated with a filled dot have bootstrap support above 90 per cent whereas those with an unfilled dot have bootstrap support above 70 per cent. All branches with less than 50 percent bootstrap support were collapsed.

RT-PCR detection was most successful using the CPMoV1138F/CPMoV1686R and CPMoV1138F/CPMoV1593R primer pairs (Table 2). When the sensitivity of the detection test was critical, the second pair could be used for a semi-nested RT-PCR. Whereas three of the 52 tested plant samples (BE273, BE276 and BE287) from the 2103 survey were found to contain detectable CPMoV-like sequence fragments using the VANA-based metagenomics approach, four of these 52 plants were found to potentially contain CPMoV RNA using the RT-PCR test (BE273, BE274, BE275 and BE276; S2 Fig). Unexpectedly, CPMoV-like sequences were detected by both approaches in only two of these plants (BE273, BE276). As the detection of CPMoV by RT-PCR required the semi-nested PCR approach, it is plausible that the concentration of viral nucleic acids in plants that tested positive by RT-PCR but negative by the VANA-based metagenomics approach may have simply been too low to detect using the metagenomics approach. However it is not understood why the detection of CPMoV by RT-PCR was negative for sample BE287 in which four reads and one contig were detected with the VANA-based metagenomics approach. Only 1/103 plants collected in Burkina Faso in 2014 tested positive for CPMoV by RT-PCR (S1 Table). Phylogenetic analysis based on a 415 nt partial *RdRp* gene unambiguously reveals that the Burkinabe CPMoV isolates are nested within the CPMoV species (Fig 1).

#### Southern cowpea mosaic virus (SCPMV)

While the four primers pairs (Table 2) enabled the amplification of the three SCPMV isolates identified using the VANA-based metagenomics approach, the primer pair SCPMVNB2698F/SCPMVNB3419R was further chosen for the detection of SCPMV (S2 Fig). Noteworthy, the primer pairs SCPMVNB2783F/SCPMVNB2916R and SCPMVNB2698F/SCPMVNB2916R could be further used for setting up either a nested PCRs or a semi-nested PCR in order to improve the sensitivity of SCPMV detection. Overall, six plant samples tested positive, including 3/307 samples collected in 2013 (that also tested positive using the VANA approach, S1 Table) and 3/103 samples collected in 2014 (S1 Table). Phylogenetic analysis based on a 495 nt partial *RdRp* gene unambiguously revealed that the SCPMV Burkinabe isolates are nested within the SCPMV species (Fig 1).

#### Cowpea-associated poleroviruses

A consensus sequence of the partial genome (5012 nt in length) of the luteovirus-like isolate infecting plant BE167 was obtained using specific primers designed from the luteovirus-like related VANA-contigs recovered from this plant. This consensus sequence corresponds to >83% of a typical polerovirus genome length. A BlastN search of GenBank returned Chickpea chlorotic stunt virus (CpCSV; accession number AY956384) as the closest match (highest percent identity = 68%, e-value = 0.0). Six ORFs were identified from this contig, including ORF0 (Beet mild yellowing virus (BMYV), accession number ACA61672, highest percent identity = 27%, e-value = 0.049), ORF1 (Phasey bean mild yellows virus (PBMYV), accession number ALR87184, highest percent identity = 33%, e-value = 9e-86), ORF2 (Brassica yellows virus (BrYV), accession number ADW41603, highest percent identity = 69%, e-value = 0.0), ORF3 (Groundnut rosette assistor virus (GRAV), accession number AAG29926, highest percent identity = 85%, e-value = 1e-94), ORF4 (CpCSV, accession number YP_667842, highest percent identity = 67%, e-value = 4e-55) and ORF3-ORF5 (Beet western yellows virus (BWYV), accession number ADR74374, highest percent identity = 81%, e-value = 2e-117). The partial genome that was obtained had an organization typical of poleroviruses in that it was comprised of six ORFs, including ORF0, which is absent in viruses of the genus Luteovirus, and ORF4, which is absent in Pea enation mosaic virus-1; which is presently the only member of the genus Enamovirus [41, 42]. Based on the current species demarcation criteria used by the ICTV *Lutoviridae* study group (less than 90% aa identity to any previously described species in any of the genes), it is likely that this virus represents a new Polerovirus species (it is hereafter referred to as Cowpea polerovirus 1; Table 1).

A second partial luteovirus-like genome fragment (3164 nt in length) was obtained by RT-PCR from plant BE179. A BlastN search revealed that this partial genome shares ~81% nucleotidic identity with PBMYV (accession number: KT963000, e-value = 0.0). Three complete ORFs identified in this sequence are most similar to the ORF2 of PBMYV (accession number: ALR87185, identity = 93%, e-value = 0.0), the ORF3 of PBMYV (accession number: ALR87186, identity = 76%, e-value = 7e-75) and the ORF4 of Suakwa aphid-borne yellows virus (SABYV; accession number: AHJ59956, identity = 56% and e-value = 7e-50). In addition, two partial ORFs were also found which were most similar to the ORF1 of PBMYV (accession number: ALR87184, identity = 71% and e-value = 8e-42) and the ORF5 of CpCSV (accession number: YP_667840, identity = 79% and e-value = 7e-85). The only canonical polerovirus ORF that was completely missing from this 3184 nt long fragment was ORF0. It is nevertheless likely that this luteovirus-like sequence is from a virus that should be classified as belonging to the Polerovirus genus of the *Luteoviridae*. It is also noteworthy that ORF2 shares >80% nucleotide sequence identity with that of PBMYV, a novel polerovirus also isolated from phasey bean (*Macroptilium lathyroides*), a legume of the *Fabaceae* family [43]. However, ORF3 and ORF4 share <80% nucleotidic identity with the corresponding ORFs of PBMYV, suggests that the new virus could potentially be considered as either a new variant of PBMYV or a new Polerovirus species. Although sequencing of the full genome of this second cowpea polerovirus will likely be required to resolve its taxonomic placement, we hereafter refer to this virus as Cowpea polerovirus 2 (Table 1).

Ten out of 52 plants collected in 2013 tested positive for the presence of poleroviruses using the primer pair designed in this study (Table 2), including plant BE168, from which no polerovirus-related reads were found using the VANA-based metagenomics approach (S1 Table and S2 Fig). Conversely, plant BE186 tested negative using the RT-PCR approach despite the recovery of polerovirus-related VANA-reads from this plant during the metagenomic screen (S1 Table). None of the samples from the 2014 sampling survey tested positive for poleroviruses using the RT-PCR assays. Based on the 233 nt partial *cp* gene sequences of these ten isolates, possible evolutionary relationships with other poleroviruses were investigated using phylogenetic analyses. While nine isolates tightly cluster around the isolate from plant BE167, from which we recovered the 5012 nt long partial genome of Cowpea polerovirus 1, the isolate BE179 branches from a different part of the tree, suggesting that this virus (Cowpea polerovirus 2) is probably a new species of cowpea-infecting polerovirus (Fig 1). However, due to the possibility of recombination (which is common in poleroviruses; [44]), further studies involving the characterization of the full genomes of these viruses are needed before it can be definitively confirmed whether or not these poleroviruses are new species.

#### Cowpea associated tombusvirids

The consensus 2142 nt long tombusvirus-like sequence obtained from plant BE81 was most similar to Panicum mosaic virus (PMV, accession number: U55002, identity = 73%, e-value = 0.006). One ORF was identified within this consensus sequence, encoding a tombusvirus-like RdRp protein that is most similar to that of Saguaro cactus virus (SCV, accession number: NP_044384, identity = 42%, e-value = 4e-79). While this result suggests that this virus, hereafter referred to as Cowpea tombusvirid 1 (Table 1), should belong to the family *Tombusviridae*, the VANA study also revealed an ORF potentially encoding a sobemovirus-like coat protein from plant BE81. However, since sobemovirus coat proteins are most similar to those found in the genus Necrovirus within the family *Tombusviridae* [45], Cowpea tombusvirid 1 can tentatively be classified in the family *Tombusviridae*.

RT-PCR detection was most successful using the Tombus2NB237F/Tombus4NB79R primer pair (Table 2), yielding a 700 bp fragment from plant BE81 as well as from three other plants (BE137, BE190 and BE197). Primer pairs Tombus3NB31F/Tombus4NB79R (Table 2 and S2 Fig) and Tombus2NB237F/Tombus3NB52R (Table 2) could be further used for setting up either a nested PCRs or a semi-nested PCR in order to improve the sensitivity of Cowpea tombusvirid 1 detection. Phylogenetic analysis based on a 660 nt *RdRp* gene fragment revealed that the four Cowpea tombusvirid 1 isolates from Burkina Faso cluster together on a branch that is not closely associated with any sequences classified within any of the established *Tombusviridae* species, suggesting that Cowpea tombusvirid 1 genome fragment is likely derived from a previously unknown tombusvirus species (Fig 1).

In addition, two other potentially novel tombusvirus-like sequences were detected in plants BE137 and BE158 using the primer pairs Tomb1NB18F/Tomb1NB193R and Tomb2NB50F/Tomb2NB172R, respectively. However, no additional plants collected in either 2013 or 2014 tested positive for these viruses. Based on the sequence of a 127 nt *RdRp* gene fragment phylogenetic analyses indicated that while the four isolates of Cowpea tombusvirid 1 cluster together tightly, the tombusvirus-like sequence from plant BE158, which we have named Cowpea tombusvirid 2 (Table 1), fall on an isolated branch in another part of the tree: suggesting that it is possibly derived from a novel tombusvirus species (S1 Fig). However, further studies will be needed to fully characterize these two tombusviruses before it can be decided whether they actually constitute new species in the family *Tombusviridae*.

#### Cowpea-associated tymovirus-like viruses

RT-PCR detection was most successful using the TymoNB120F/TymoNB415R primer pair (Table 2), yielding a 255 bp partial replication-associated polyprotein gene fragment from 5 cowpea samples collected in 2013 and 1 in 2014 (S1 Table and S2 Fig). Because of the extremely distant relationships that existed between these 255 nt amplicons and homologous sequences found in known tymovirus species, it was not possible to accurately align the sequences. However, tymovirus-like amplicons shared high degrees of similarity with sequences of a novel mycotymovirus species that has recently been characterized from the plant pathogenic fungus *Fusarium graminearum*, suggesting that the probable tymovirus-like virus species detected here (which will hereafter referred to as Cowpea associated mycotymovirid 1) is potentially a second member of the new mycotymovirus lineage of the family *Tymoviridae* [40].

### Symptomatology of cowpea plants collected in Burkina Faso

Field-sampled plants displayed a large range of symptom types (S1 Table and S2 Fig), including mild mosaic, severe mosaic, yellowing, mottling, leaf distortion, vein chlorosis and necrosis. However, since the majority of the cowpea plants infected by the novel viruses were also co-infected by potyviruses, it was not possible to clearly assign specific types of symptom to particular viruses. It is, however, noteworthy that plant BE81, which is apparently only infected by Cowpea tombusvirid 1 (S1 Table), displayed symptoms of leaf distortion (S3 Fig). Altogether, these results indicate that the virus pressure on cowpea plants is relatively high in Burkina Faso and suggests that the virus-related sequences identified in this study are probably part of functional viruses that could potentially have a detrimental impact on cowpea production.

### Detection sensitivities of VANA-based metagenomics and RT-PCR methods

Overall, RT-PCR assay sensitivities were found to be slightly higher than that of the VANA-based metagenomic assay (Table 4). While neither approach detected any viruses in the field plants scored as asymptomatic, RT-PCR assay sensitivities were slightly better for detecting plant viruses from field cowpea samples scored as being symptomatic (Table 4). Several plants infected with CPMoV (3/52 detected by VANA and 4/52 detected by RT-PCR), Cowpea tombusvirid 1 (1/52 by VANA, 4/47 by RT-PCR) and the Cowpea associated mycotymovirid 1 (1/47 by VANA, 5/47 by RT-PCR) were missed by the VANA-based approach (Table 4). Consequently, RT-PCR revealed a few more cases of viral co-infection than were revealed by the VANA-based metagenomics screen, including cases of triple and quadruple infections (Table 4). We hypothesize that the reduced efficiency of the random priming VANA-based approach compared to the specific priming RT-PCR approach can be accounted for by the relatively high numbers of mixed infections occurring in the subset of 52 cowpea samples (20/52; 38.46%), that may have hampered the detection of all co-infecting viruses using the VANA-based approach.

**Table 4:** Virus prevalence and mixed infection prevalence of 52 cowpea plants based on VANA-based and RT-PCR-based detection results.

Virus infection	VANA	RT-PCR
Symptomatic field samples	40/44 (90.9%)	41/44 (93.2%)
Asymptomatic field samples	0/3 (0%)	0/3 (0%)
Symptomatic “seedlings” samples	4/4 (100%)	1/4 (25%)
Asymptomatic “seedlings” samples	1/1 (100%)	0/1 (0%)
CABMV	41/52 (78.84%)	38/52 (73.07%)
BCMV-BlCM	2/52(3.84%)	2/52 (3.84%)
CPMoV	3/52(5.77%)	4/52 (7.69%)
SCPMV	3/52 (5.77%)	3/52 (5.77%)
Cowpea polerovirus 1	9/52 (17.3%)	9/52 (17.3%)
Cowpea polerovirus 2	1/52 (1.9%)	1/52 (1.9%)
Cowpea tombusvirid 1	1/52 (1.9%)	4/52 (1.9%)
Cowpea tombusvirid 2	1/52 (1.9%)	1/52 (1.9%)
Cowpea associated mycotymovirid 1	1/52 (1.9%)	5/52 (9.6%)
Single viral infection^a^	28/52 (53.84%)	22/52 (42.3%)
Double viral infection^b^	16/52 (30.77%)	15/52 (28.84%)
Triple viral infection^c^	1/52 (1.9%)	4/52 (7.69%)
Quadruple viral infection^d^	0.00%	1/52 (1.9%)

^a^: single infections consist of infection of: CABMV; BCMV-BlCM; Cowpea tombusvirid 1 or SCPMV

^b^: double infections consist of mixed infection of: CABMV / SCPMV; CABMV / CPMoV; CABMV / Cowpea associated mycotymovirid 1; CABMV / Cowpea tombusvirid 2; CABMV / Cowpea polerovirus 1; BCMV-BlCM / Cowpea polerovirus 1 or CABMV / Cowpea polerovirus 2

^c^: triple infections consist of mixed infection of: CABMV / SCPMV / CPMoV; CABMV / Cowpea tombusvirid 2 / Cowpea associated mycotymovirid 1 or CABMV / Cowpea polerovirus 1 / Cowpea tombusvirid 1

^d^: quadruple infections consist of mixed infection of: CABMV / Cowpea polerovirus 1 / Cowpea tombusvirid 1 / Cowpea associated mycotymovirid 1

### Geographic distribution and prevalence of cowpea-infecting viruses in Burkina Faso

Among the various groups of viruses identified using the VANA-based metagenomic approach, the potyvirus CABMV is the most prevalent within cowpea grown in Burkina Faso. Whereas CABMV was found in 195/201 (97.0%) of the plants testing positive for potyviruses, BCMV-BlCM was found in only 6/201 (2.99%) of these plants.

The prevalence of viruses from other families were low: 10/307 (3.26%) for the poleroviruses, 3/307 (0.98%) for the carmoviruses, and 3/307 (0.98%) for the sobemoviruses. Nevertheless, the prevalence of all cowpea viruses (other than perhaps the potyviruses) was probably slightly under-estimated because the detection rate of the VANA-based approach may have been reduced due to the high frequency of viral co-infections as evidenced by the comparison of RT-PCR/VANA cowpea virus detection approaches.

**Fig 2:**
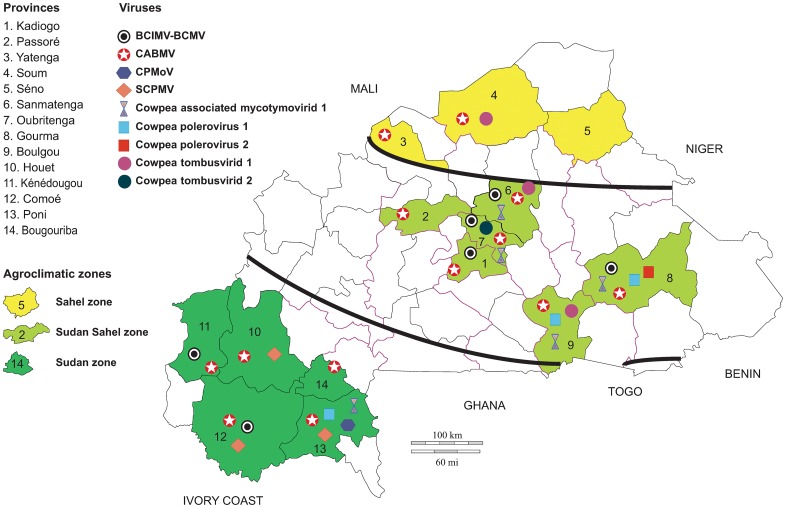
Geographical distribution and prevalence of Cowpea viruses in Burkina Faso.

While the five taxonomic viral groups occur in the Sudan zone (*Potyviridae*, sobemovirus, *Luteoviridae*, *Tombusviridae* and *Tymoviridae*) and four in the Sudan-Sahel zone (*Potyviridae*, *Luteoviridae*, *Tombusviridae* and *Tymoviridae*), only two taxonomic groups are present in the Sahel zone (*Potyviridae* and *Tombusviridae*, Fig 2). The percentage of plants infected with potyviruses decreased between the Sudan zone (87/101, 86.1%) and Sahel zone (15/65, 23.07%). This gradient, which was already reported in a previous study [46] can be accounted for by climatic conditions in Burkina Faso, which are more favourable for the growth and maintenance of insect populations in the Sudan zone which, in turn, favors the transmission of plant viruses in the Sudan and Sudan-Sahel zones [47].

By contrast, both CPMoV and SCPMV are only present in the Sudan zone (Fig 2). While CPMoV was already reported in Burkina Faso in 1989 [48], this is the first report of the occurrence of SCPMV in this country. The epidemiological dynamics of SCPMV will need to be monitored because, as has been reported for other African countries, this virus could become an important constraint on cowpea production in Burkina [16, 49, 50].

Poleroviruses were mainly detected from the Sudan-Sahel zone (9/141, 6.38%) although one isolate was found in the Sudan zone (1/101, 0.99%). Tombusvirus-like viruses were identified from the Sahel zone (Cowpea tombusvirid 1, 1/65) and the Sudan-Sahel zone (Cowpea tombusvirid 1, 4/65, and Cowpea tombusvirid 2, 1/141). Finally, the Cowpea associated mycotymovirid 1 was identified in the Sudan-Sahel zone firstly with VANA-based approach in one sample (1/141, 0.7%), whereas in RT-PCR method other positive samples were detected in both the Sudan and Sudan-Sahel zones.

The occurrence of potyviruses and poleroviruses in mixed infection can be related to the fact that these viruses are both aphid transmitted [51, 52], while the occurrence of SCPMV and CPMoV may be linked to the fact that both these viruses are beetle transmitted [53, 54].

### Detection of seed-borne cowpea-infecting viruses

Plant virus-associated VANA-reads were found from 16.66% (12/72) of the cowpea seedlings grown in an insect-proof growth chamber in France (UMR BGPI, CIRAD, Montpellier). While eight cowpea-infecting viruses (BCMV-BlCM, CABMV, SCPMV, CPMoV, CPMV, CPSMV, CPMMV and CMV) are reported to be potentially seed-transmissible (reviewed in [55]), only potyvirus-related reads were obtained from the cultivars Nafi, Tiligré, Yiis-yandé, Kvx61-1 and the unknown Togo cultivar (S1 Table). The rate of seedlings infected by potyviruses was highly variable and ranged from 0% to 100% for specific cultivars. Seedlings from the unknown Togo cultivar were all infected (100%), while seedlings from the Burkina Faso cultivars were heterogeneously infected, ranging from 0% (for 4 cultivars) to 12.5% (cultivars Tiligré, Yiis yandé and Kvx61-1) and 25% (cultivar Nafi). BCMV-BlCM was the only potyvirus species that was detected from the Togo cultivar while both BCMV-BlCM and CABMV were detected from the Burkina cultivars.

Overall, these results highlight the fact that potyvirus seed-transmission rates are likely high in Togo and Burkina Faso: a fact that could certainly have a major impact on the recurrence of diseases associated with potyviruses in this African region and can partly account for the very high prevalence of potyvirus infections in cowpeas grown throughout Burkina Faso. Minimizing or removing this primary source of viral inoculum would probably be a first step towards better control of potyvirus diseases of cowpea within this country.

## Conclusion

Overall, a combination of VANA-based metagenomics and classical RT-PCR- based molecular detection approaches have strengthened our knowledge about the diversity of viruses infecting cowpea in Burkina Faso; which is a first step towards minimizing the economic burden of these viral diseases on the smallholder farmers whose are the principal producers of legumes both in this country, and the rest of west Africa. The cowpea viruses identified in this study should be further studied and taken into account in future efforts to control diseases in this important crop.

## Supporting Information

S1 Fig Maximum-likelihood phylogenetic trees depicting the relatedness of potyvirus and tombusvirus-like viruses from Burkina Faso. A) Maximum-likelihood phylogenetic trees of partial nuclear inclusion genes from 21 isolates of Cowpea polerovirus and representative species from the family *Potyviridae*. CABMV, Cowpea aphid-borne mosaic virus; BCMV, Blackeye cowpea mosaic strain of Bean common mosaic virus; PPST, Passiflora virus; ABMV, Azuki bean mosaic virus; HMV, Hardenbergia mosaic virus. B) Maximum-likelihood phylogenetic trees of partial *RdRp* genes from five isolates of Cowpea tombusvirus-like virus and representative species from the family *Tombusviridae*. OCSV, Oat chlorotic stunt virus; TBTV, Tobacco bushy top virus; SCNMV, Sweet clover necrotic mosaic virus; CRSV, Carnation ringspot virus; FNSV, Furcraea necrotic streak virus; GaMV, Galinsoga mosaic virus; OLV1, Olive latent virus 1; TNV A, Tobacco necrosis virus A; JCSMV, Johnsongrass chlorotic stripe mosaic virus; MWLMV, Maize white line mosaic virus; MNSV, Melon necrotic spot virus; PNSV, Pelargonium necrotic spot virus; CIRV, Carnation Italian ringspot virus; LWSV, Leek white stripe virus; BBSV, Beet black scorch virus; TurCV, Turnip crinkle virus; CarMV, Carnation mottle virus; MCMV, Maize chlorotic mottle virus; PMV, Panicum mosaic virus; CkMMV, Cocksfoot mild mosaic virus; PMV, Panicum mosaic panicovirus.
(EPS)

S2 Fig Agarose gel illustrating RT-PCR detection of cowpea-infecting viruses. (A) RT-PCR for detection of potyviruses (B), RT-PCR for detection of Cowpea mottle virus (C), RT-PCR for detection of Southern cowpea mosaic virus (D), RT-PCR for detection of Cowpea polerovirus1 and Cowpea polerovirus2 (E) Nested RT-PCR for detection of Tombusvirid1 and (F) RT-PCR for detection of Cowpea associated mycotymovirid 1.
(DOC)

S3 Fig Symptoms observed on plants naturally infected by Cowpea-infecting viruses. (DOCX)

S1 Table List of cowpea viruses detected using VANA-based metagenomic and RT-PCR approaches. (DOCX)

S2 Table 10-nucleotide multiplex identifier (MID) tagged DNA primers used for PCR from cDNA. (DOC)

S3 Table List of NCBI GenBank sequences used for designing primers. (DOCX)

S4 Table List of primers used for Cowpea polerovirus 1 and Cowpea polerovirus 2 partial genome amplification. (DOCX)
