# Supplementary material for: Metagenomic-Based Screening and Molecular Characterization of Cowpea-Infecting Viruses in Burkina Faso
Source: PLoS One. 2016 Oct 20;11(10):e0165188. doi: 10.1371/journal.pone.0165188 (PMC5072566; doi:10.1371/journal.pone.0165188)

**A)** RT-PCR for detection of potyviruses (Cowpea aphid-borne mosaic virus, CABMV and Blackeye cowpea mosaic virus – a strain of Bean common mosaic virus, BCMV-BlCMV) using the universal primers pair Oligo1N/Oligo2N. **M**, Invitrogen 1 Kb Plus DNA Ladder; lane 1 to 6 and 8, plant samples testing positive for CABMV (lane 1, BE146; 2, BE151; 3, BE155; 4, BE214; 5, BE212; 6, BE1 and 8, BE4), lane 7, BE3 plant sample testing positive for BCMV-BlCMV; lane 9, water; lane 10, Sugarcane mosaic virus positive control.


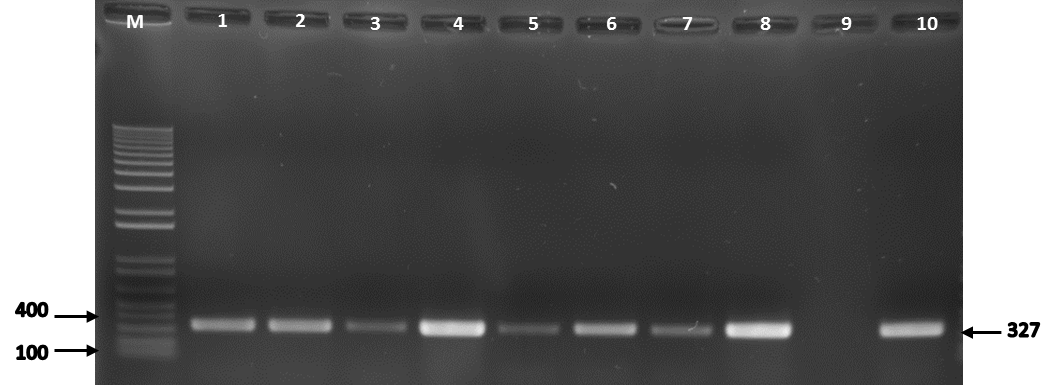


**B)** RT-PCR for detection of Cowpea mottle virus (CPMoV) using the detecting primers CPMoV1138F/CPMoV1686R. **M**, Invitrogen 1 Kb Plus DNA Ladder; lane 1, BE197; 2, BE198; 3, BE199; 4, BE250; 5, BE251; 6, BE252; 7, BE254; 8, BE273; 9, BE274; 10, BE275; 11, BE276; 12, healthy *Vigna unguiculata*; 13, water.


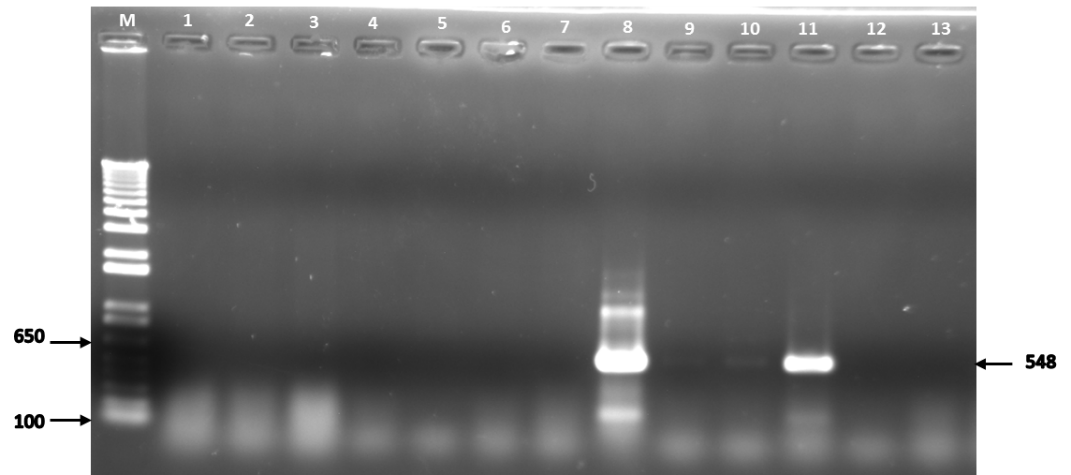


**C)** RT-PCR for detection of Southern cowpea mosaic virus (SCPMV) in the core sample using the detecting primers SCPMVNB2698F/SCPMVNB3419R. **M**, Invitrogen 1 Kb Plus DNA Ladder; lane 1, BE197; 2, BE198; 3, BE199; 4, BE250; 5, BE251; 6, BE252; 7, BE254; 8, BE273; 9, healthy *Vigna unguiculata*; 10, water.

**
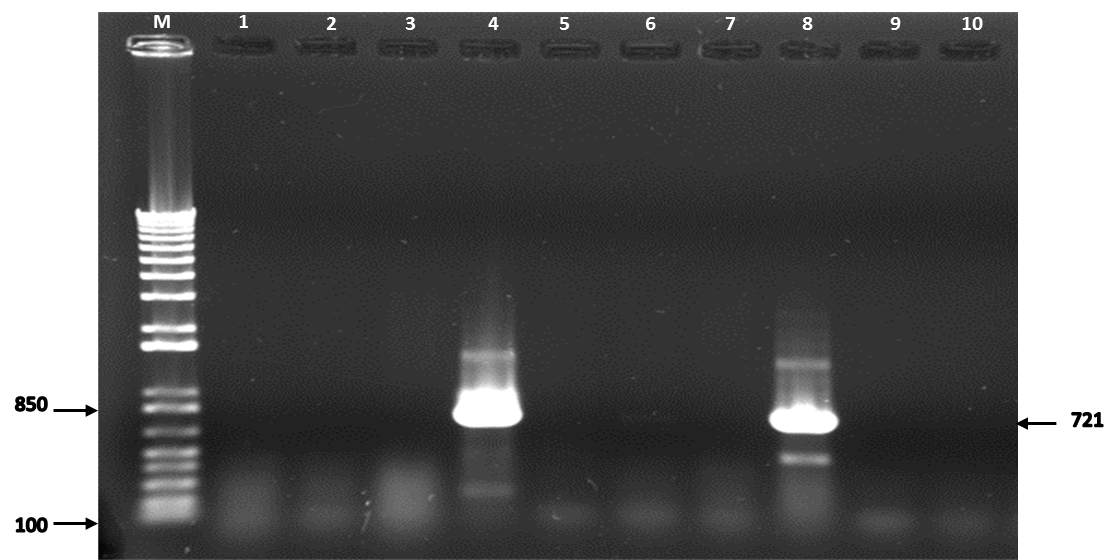
**

**D)** RT-PCR for detection of Cowpea polerovirus1 and Cowpea polerovirus2 in the core sample using generic detecting primers: PoleroNB3897F/ PoleroNB4160R. **M**, Invitrogen 1 Kb Plus DNA Ladder; lane 1, BE158; 2, BE159; 3, BE160; 4, BE162; 5, BE167; 6, BE168; 7, BE169; 8, BE170; 9, BE172; 10, BE173; 11, BE179; 12, BE180; 13, BE186; 14, BE187; 15, BE190; 16, water.


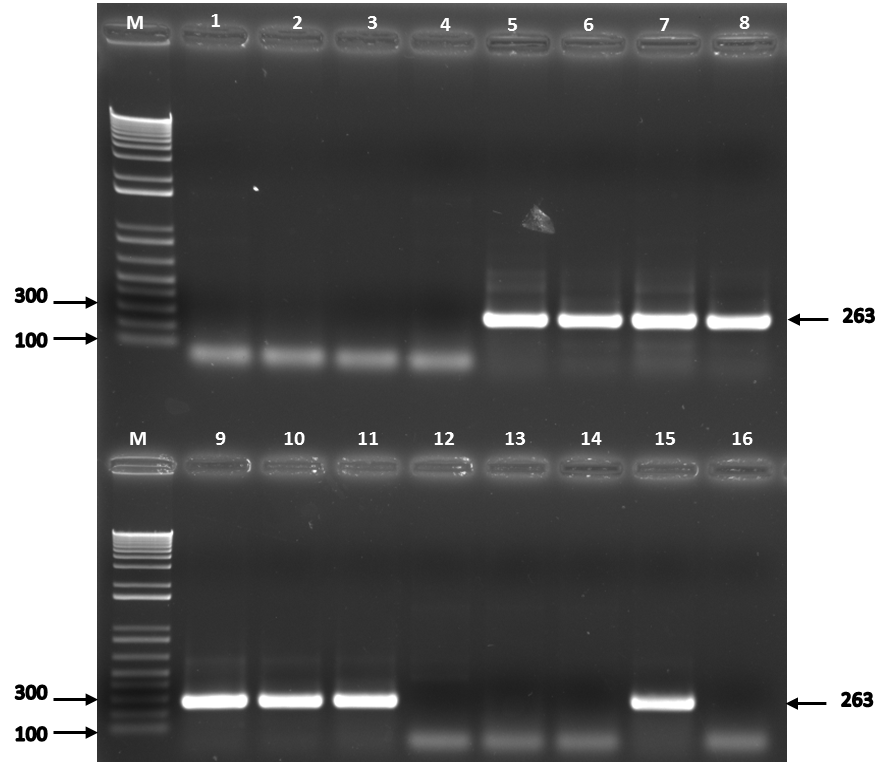


**E)** Nested RT-PCR for detection of Tombusvirid1 in the core sample using the primers pair Tombus3NB31F / Tombus4NB79R. **M**, Invitrogen 1 Kb Plus DNA Ladder; lane 1, BE120; 2, BE81; 3, BE121; 4, BE138; 5, BE139; 6, BE158; 7, BE162; 8, BE180; 9, BE137; 10, healthy *Vigna unguiculata*; 11, water.


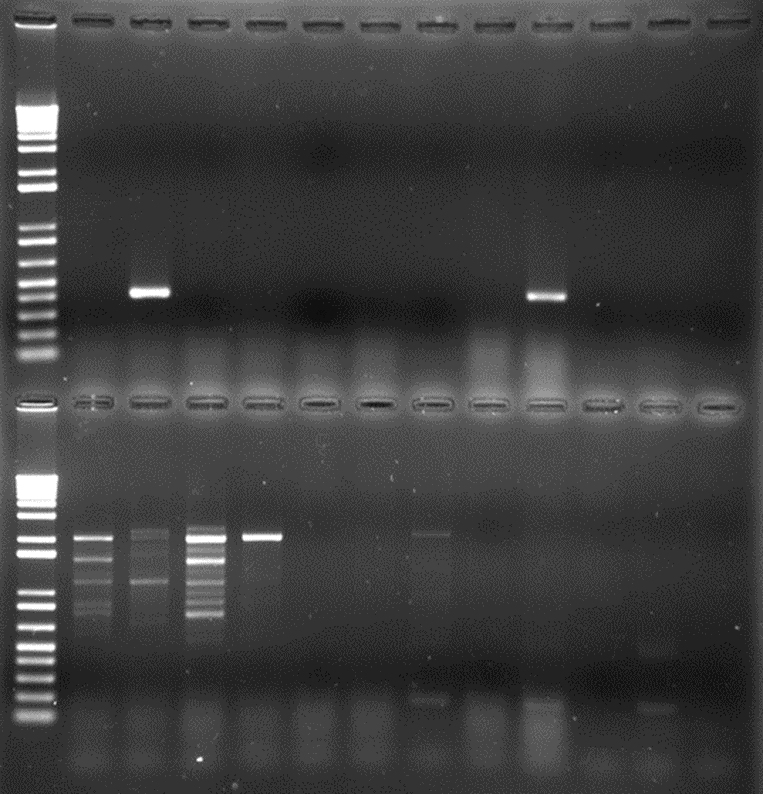


**M**

**5**

**6**

**7**

**8**

**10**

**9**

**4**

**3**

**2**

**1**

100

400

412412

**11**

**F)** RT-PCR for detection of Cowpea associated mycotymovirid 1 in the core sample using primers pair TymoNB120F / TymoNB415R. **M**, Invitrogen 1 Kb Plus DNA Ladder; lane 1, BE158; 2, BE159; 3, BE160; 4, BE190; 5, BE198; 6, BE273; 7, BE275; 8, BE276; 9, BE278; 10, BE120; 11, healthy *Vigna unguiculata*; 12, water.


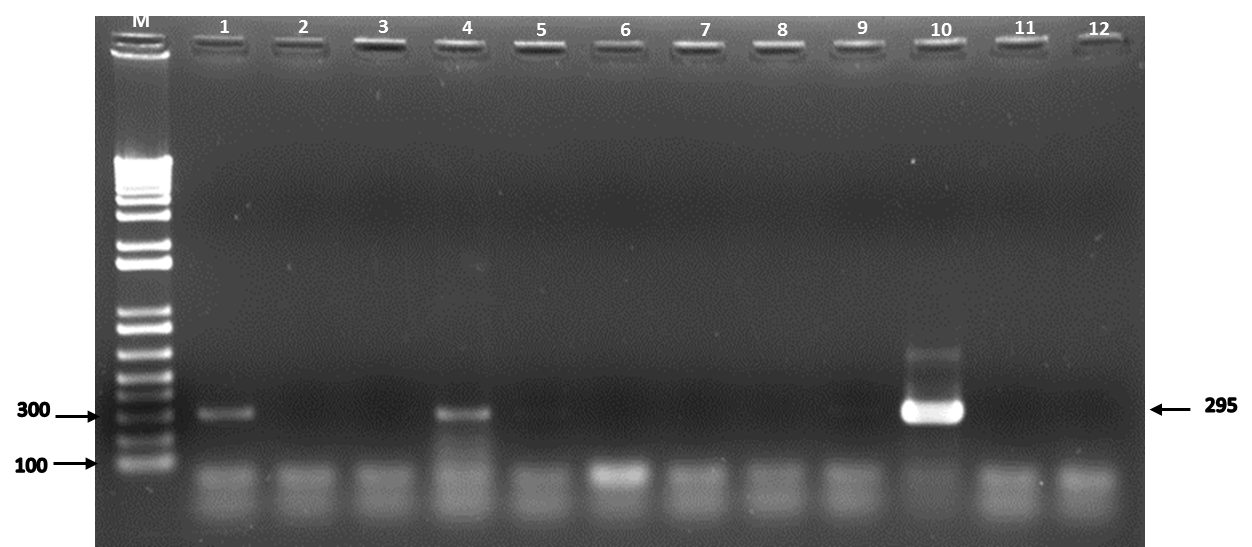

Supplement: S2 Fig — (A) RT-PCR for detection of potyviruses (B), RT-PCR for detection of Cowpea mottle virus (C), RT-PCR for detection of Southern cowpea mosaic virus (D), RT-PCR for detection of Cowpea polerovirus1 and Cowpea polerovirus2 (E) Nested RT-PCR for detection of Tombusvirid1 and (F) RT-PCR for detection of Cowpea associated mycotymovirid 1. (DOC) [file pone.0165188.s002.doc]
