# Supplementary material for: Metagenomic-Based Screening and Molecular Characterization of Cowpea-Infecting Viruses in Burkina Faso
Source: PLoS One. 2016 Oct 20;11(10):e0165188. doi: 10.1371/journal.pone.0165188 (PMC5072566; doi:10.1371/journal.pone.0165188)
Supplement: S3 Fig — (DOCX) [file pone.0165188.s003.docx]

**Symptoms observed on plants naturally infected by Cowpea aphid-borne mosaic virus (CABMV)**

**A.** Mosaic (BE255); **B.** Mild mosaic (BE214); **D.** Vein yellow mosaic (BE212); **E.** Yellow mosaic (BE6).


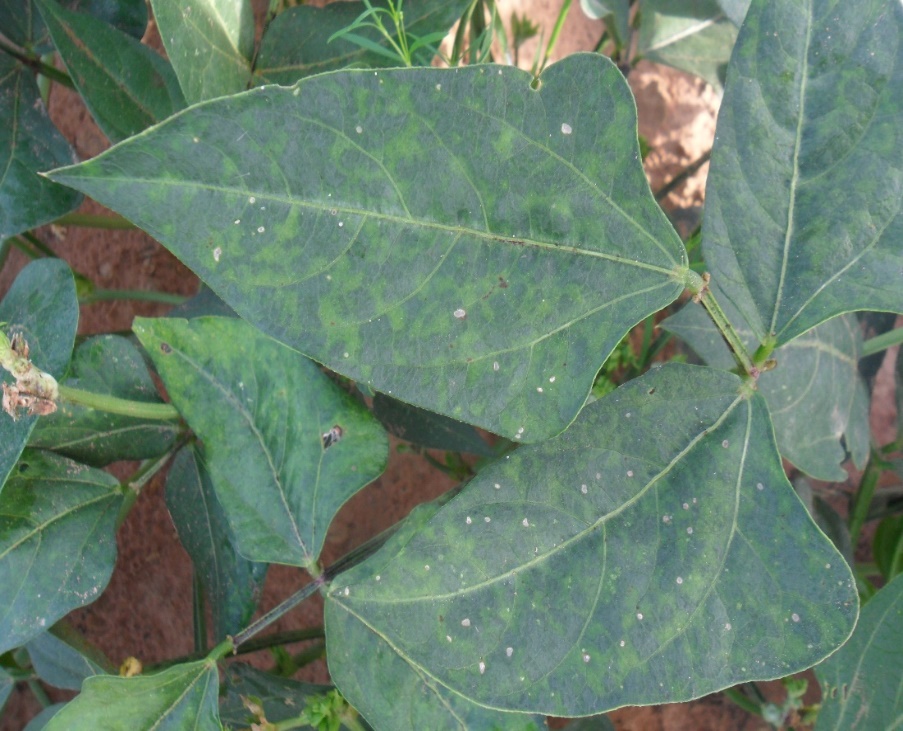


C. BE214


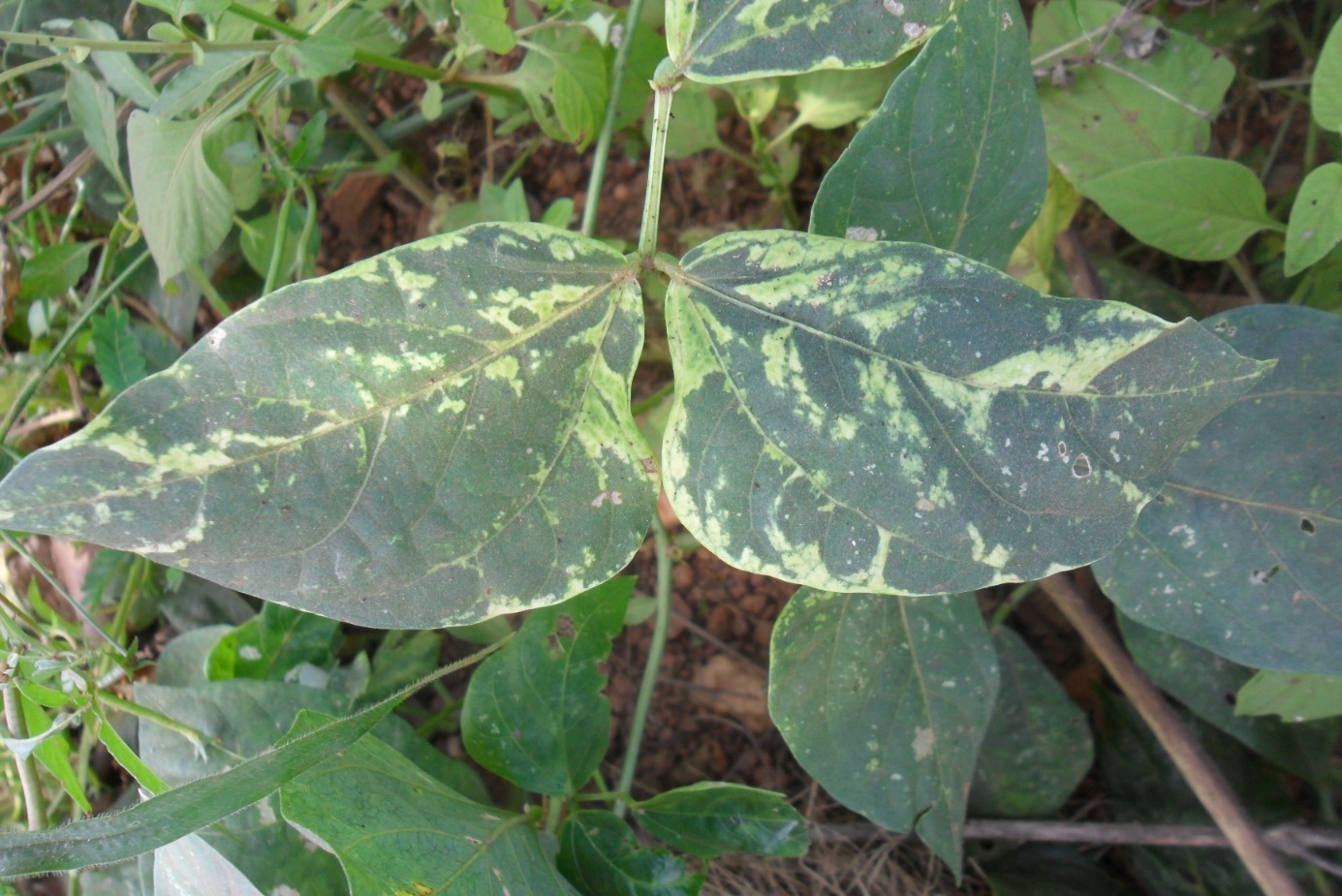


A. BE255


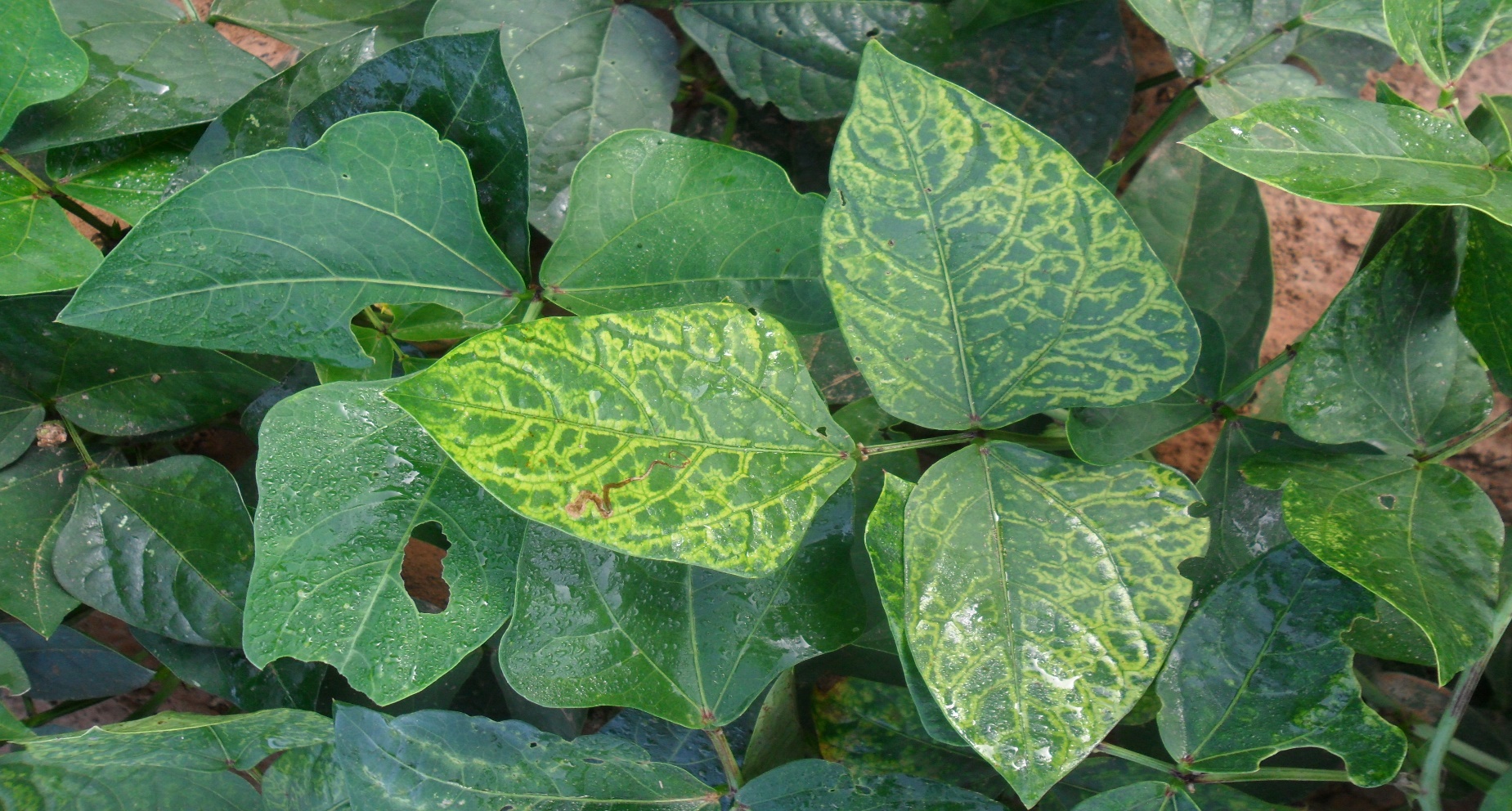


D. BE212


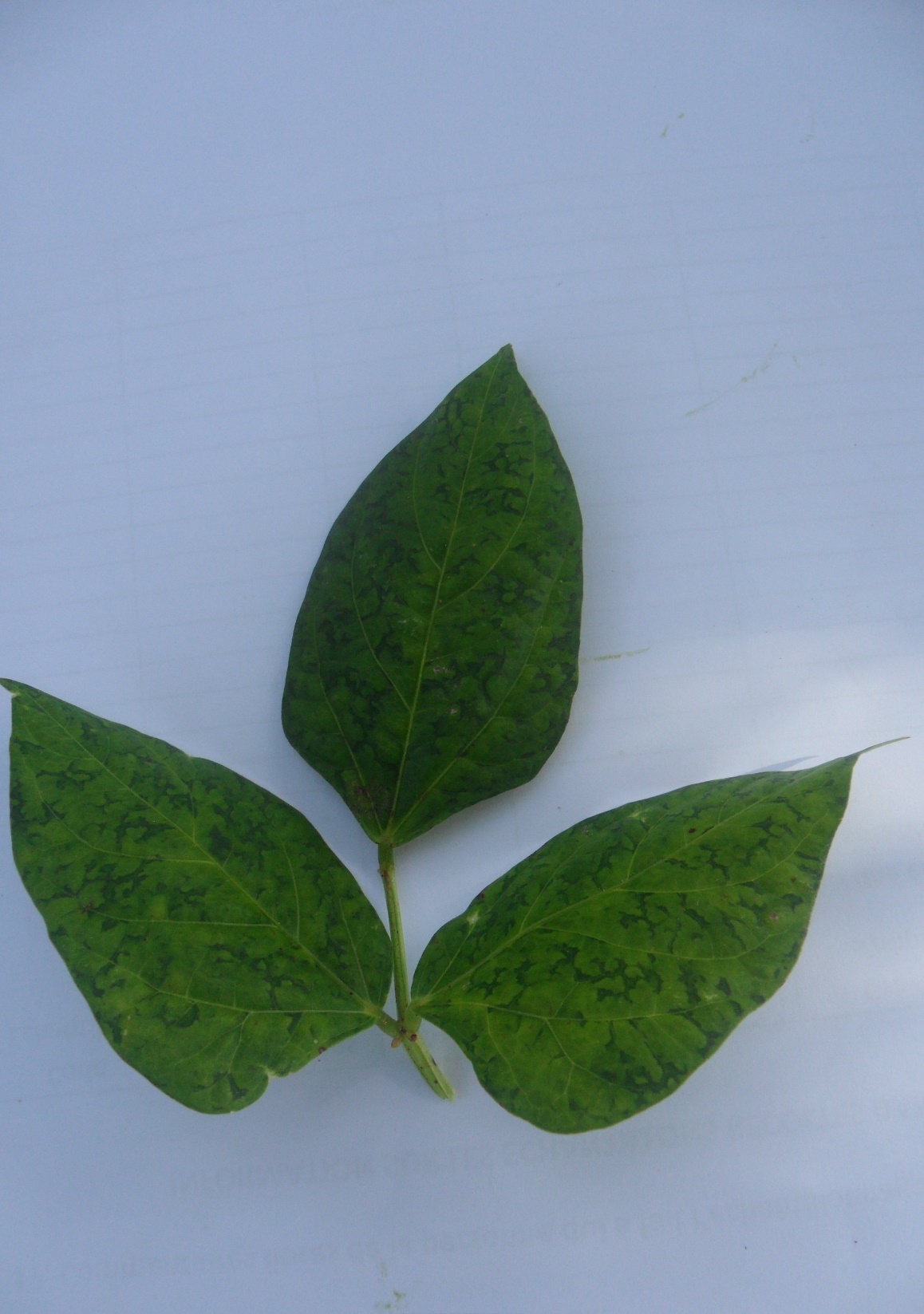


E. BE6

**Symptoms observed on cowpea plants naturally co-infected by CABMV and Cowpea mottle virus (CPMoV)**

**A.** Mosaic (BE274); **B.** Mild mosaic (BE275)


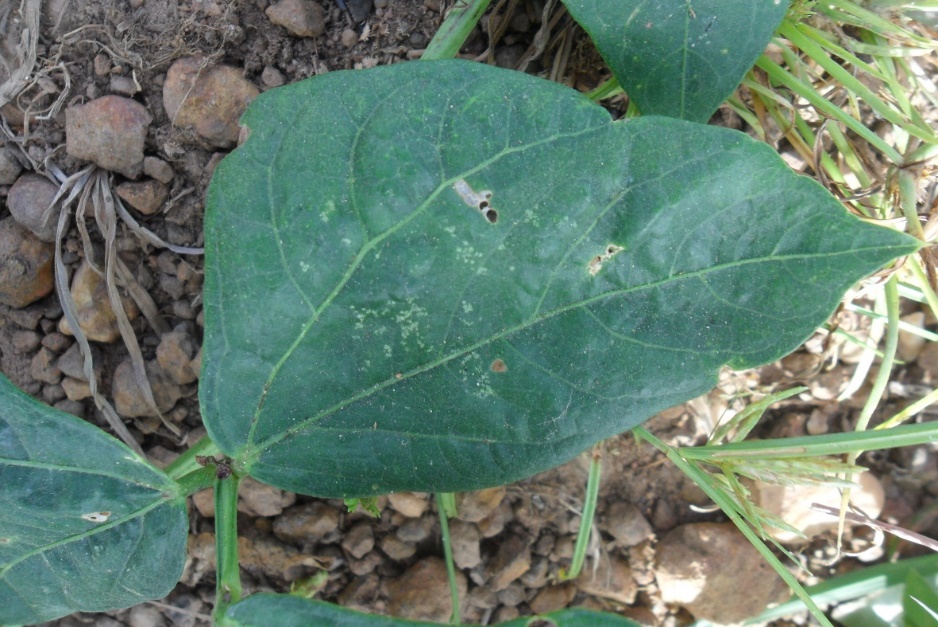


B. BE275


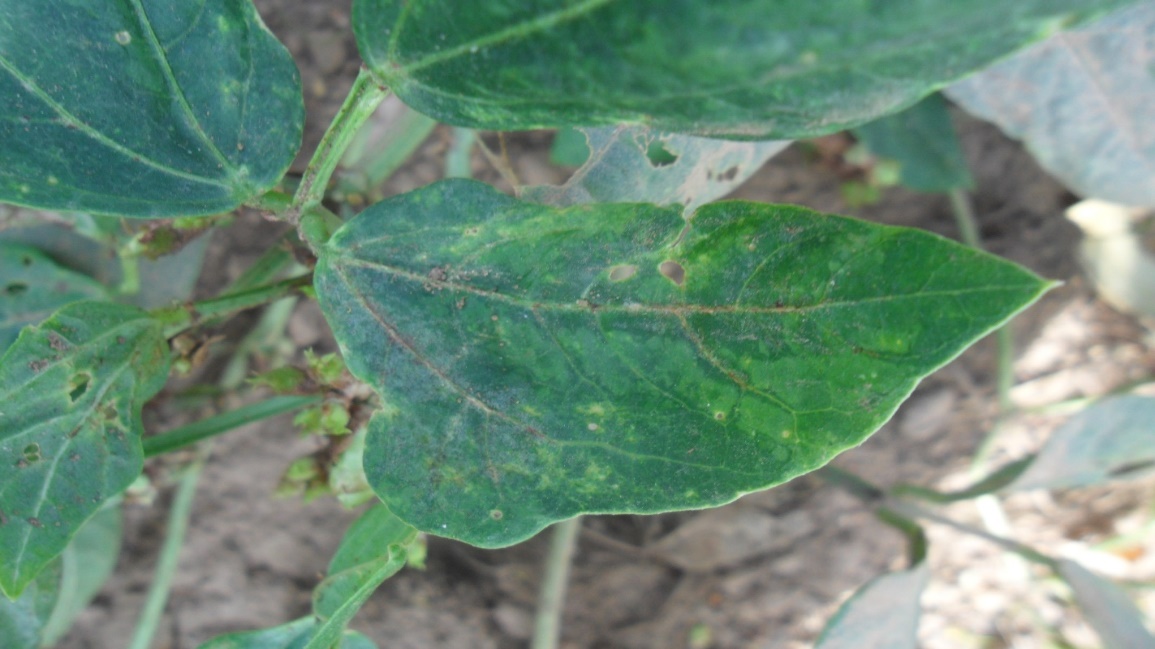


A. BE274

**Symptoms observed on cowpea plants naturally co-infected by one polerovirus and CABMV.**

**A.** Yellow mosaic (BE167, Cowpea polerovirus 1 and CABMV); **B.** Yellowing following vein (BE168, Cowpea polerovirus 1 and CABMV); **C.** Interveinal yellowing (BE170, Cowpea polerovirus 1 and CABMV) ; **D.** Mild yellow mosaic (BE179, Cowpea polerovirus 2 and CABMV).


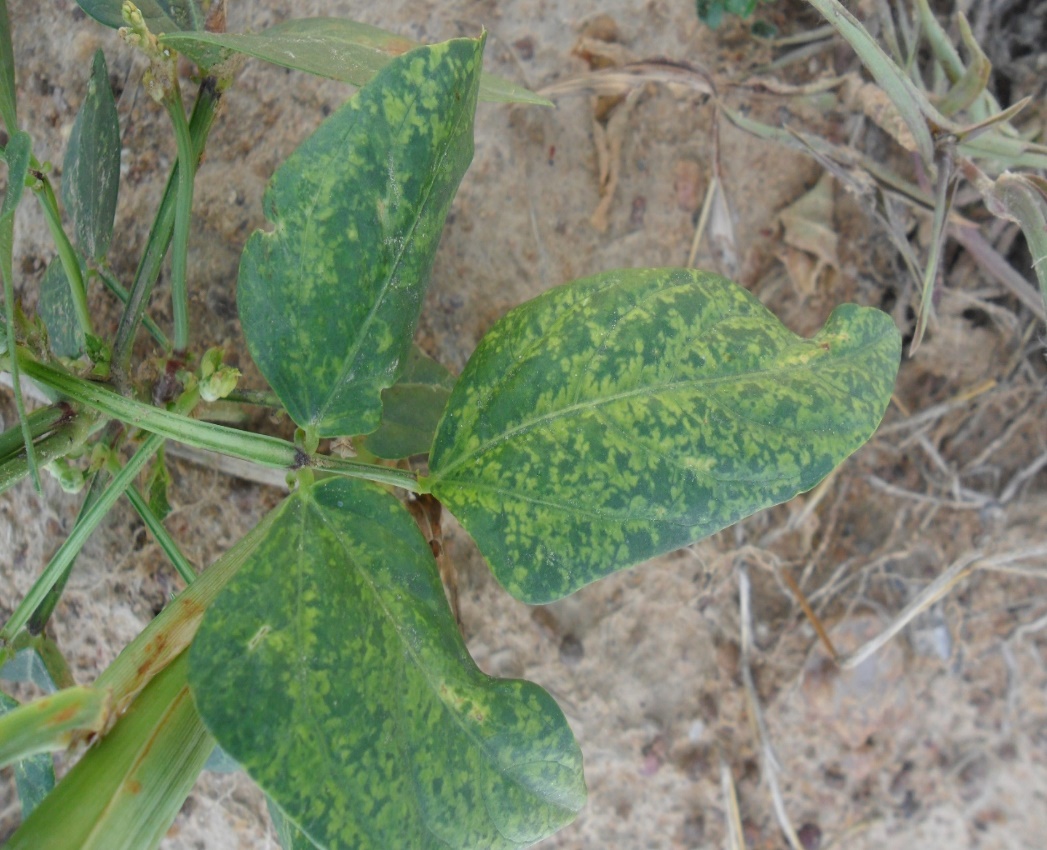


B. BE168


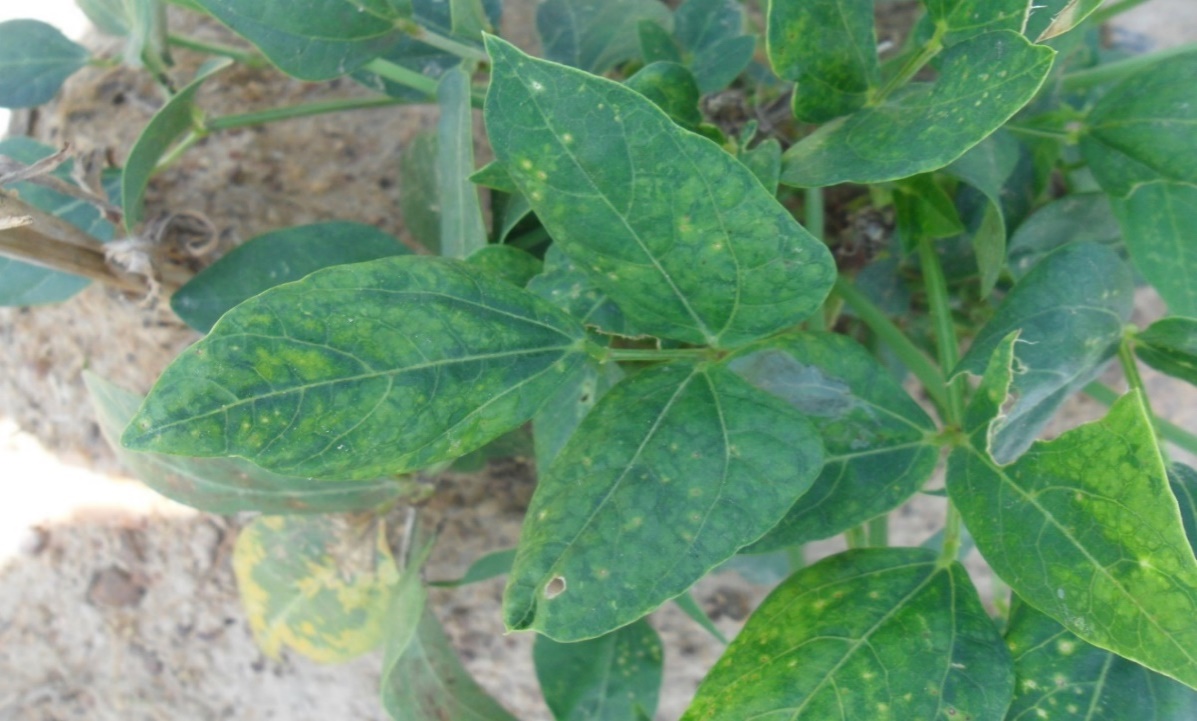


A. BE167


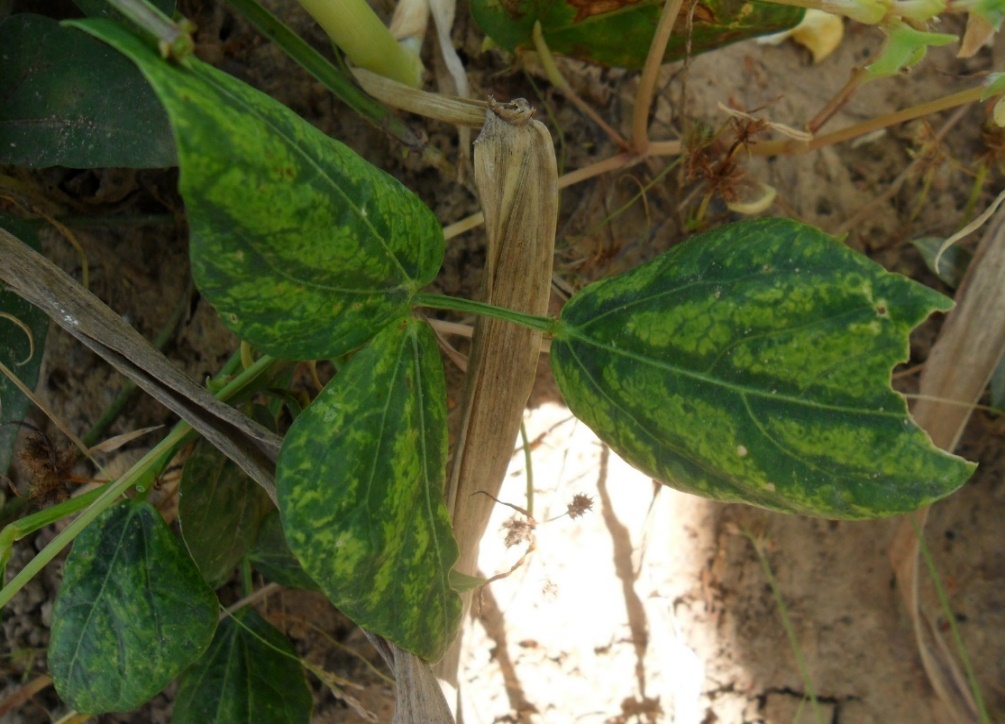


C. BE170


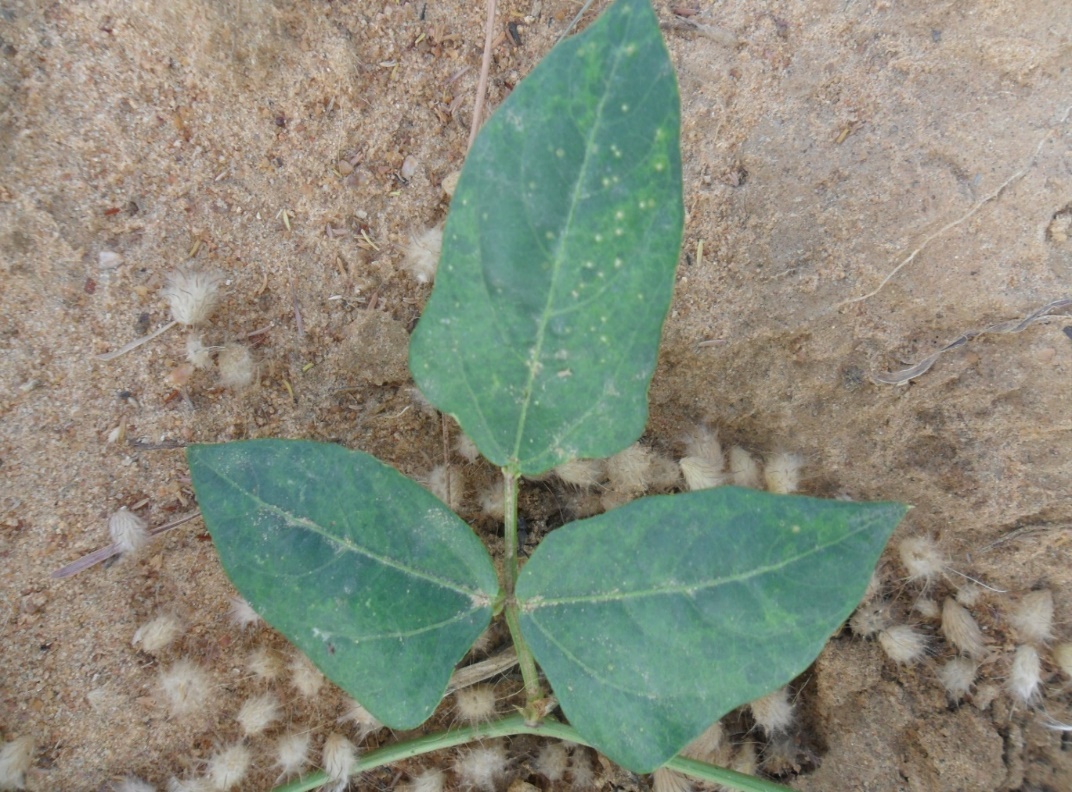


E. BE179

**Symptoms observed on cowpea plants naturally infected by Southern cowpea mosaic virus (SCPMV).**

Mild mosaic on sample BE250 (**A**) and sample BE28-14 (**B**)


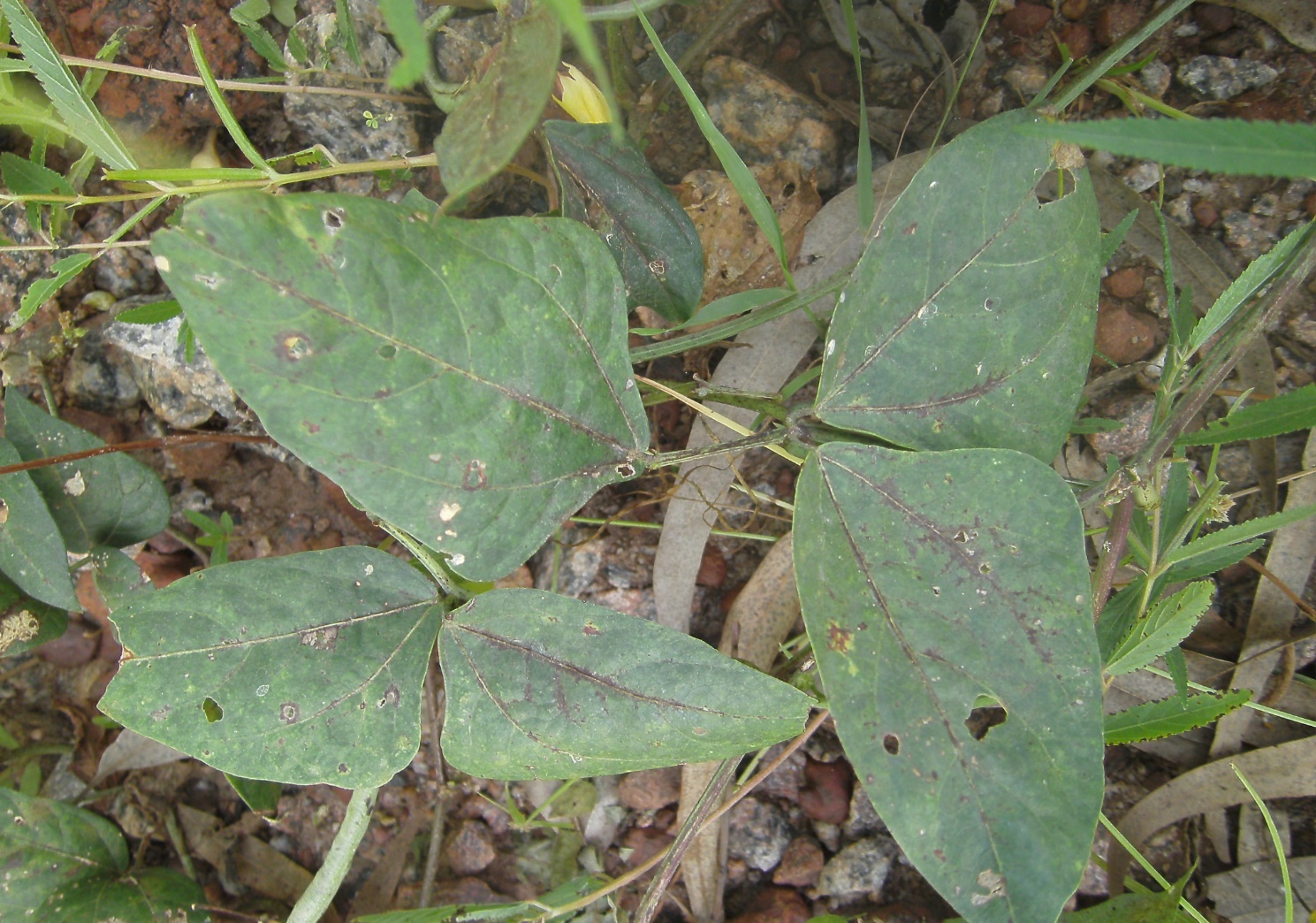


B. BE28-14


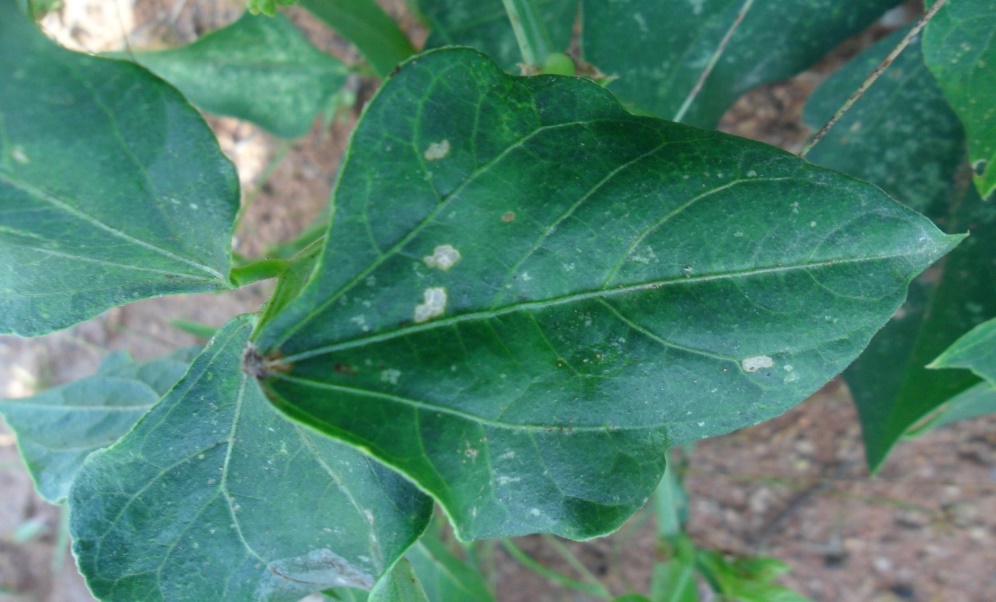


A. BE250

**Symptoms observed on plants naturally infected by Blackeye cowpea mosaic virus – a strain of Bean common mosaic virus (BlCMV-BCMV)**

**A.** Vein yellow mosaic (BE3); **B.** Mosaic (BE230).


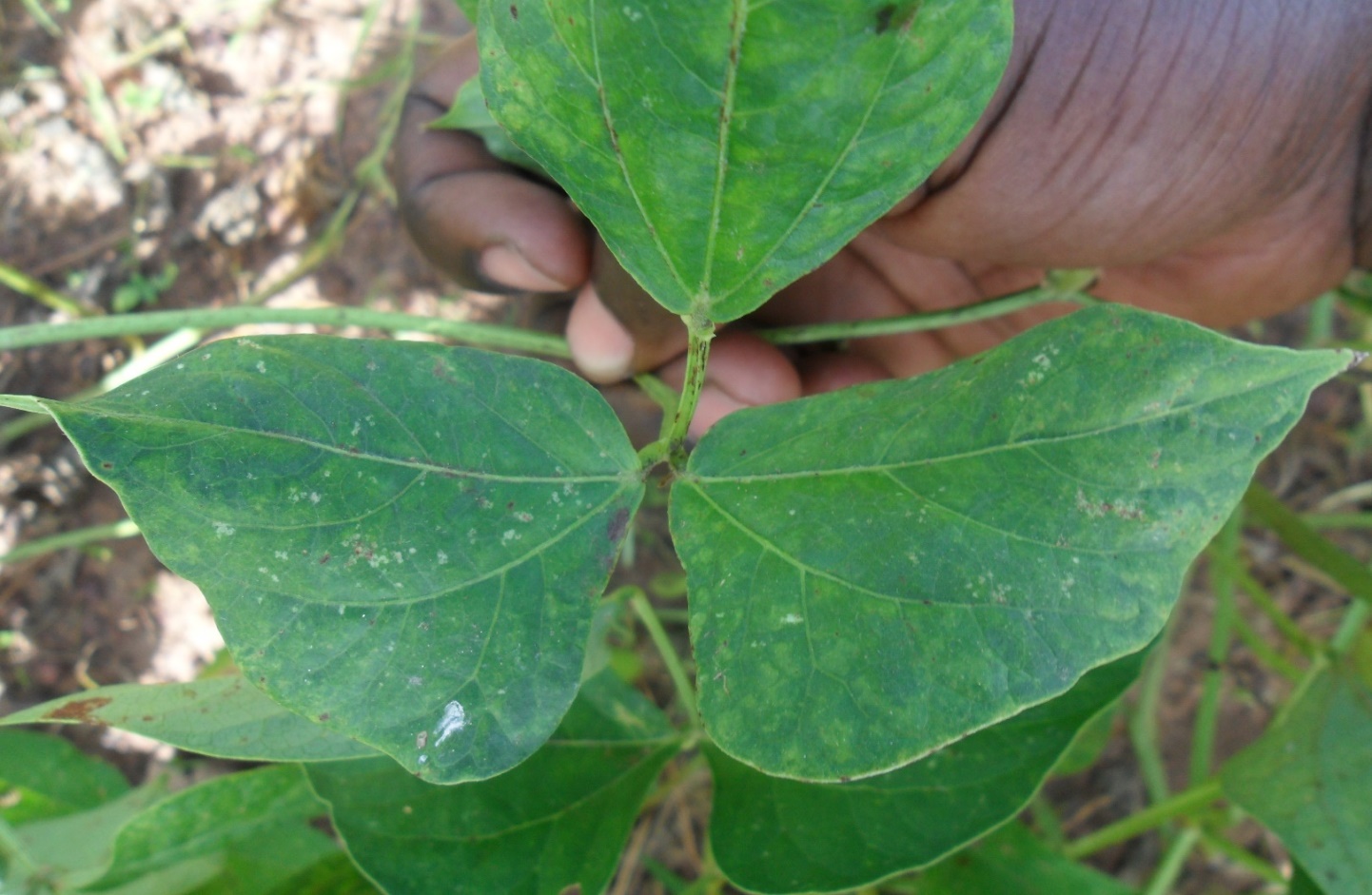


C. BE230


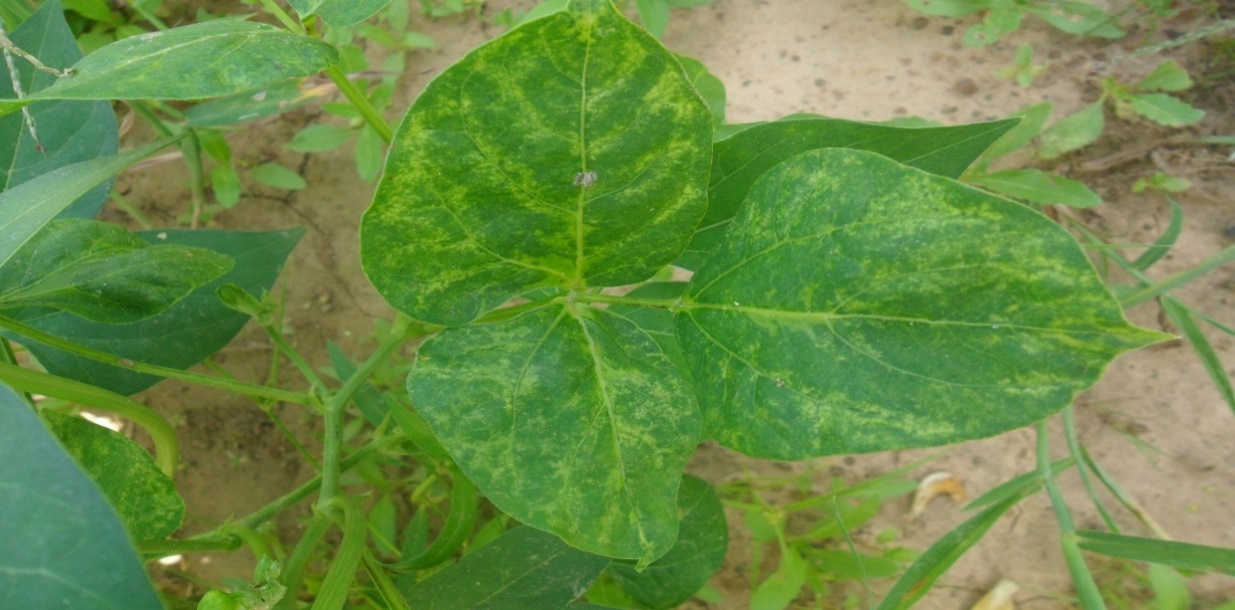


A. BE3

**Symptoms observed on plants naturally infected by Cowpea tombusvirid 1**

**A.** Leaf distorsion (BE81).


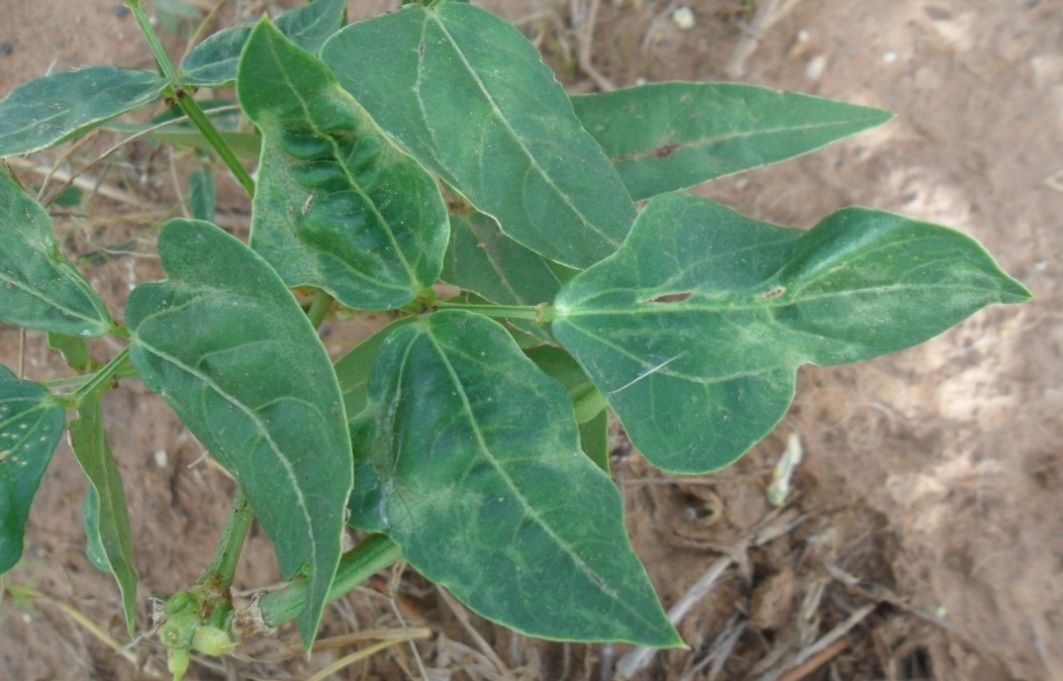


BE81
