# Supplementary material for: Metagenomic-Based Screening and Molecular Characterization of Cowpea-Infecting Viruses in Burkina Faso
Source: PLoS One. 2016 Oct 20;11(10):e0165188. doi: 10.1371/journal.pone.0165188 (PMC5072566; doi:10.1371/journal.pone.0165188)
Supplement: S1 Table — (DOCX) [file pone.0165188.s004.docx]

**Supplementary Table 1:** List of cowpea viruses detected using VANA-based metagenomic and RT-PCR approaches.

|  |  |  |  |  |  | NGS/RT-PCR | NGS/RT-PCR | NGS/RT-PCR | NGS/RT-PCR | NGS/RT-PCR | NGS/RT-PCR | NGS/RT-PCR | NGS/RT-PCR |
| --- | --- | --- | --- | --- | --- | --- | --- | --- | --- | --- | --- | --- | --- |
| Sample | Zone | Symptomatology | Country (Provinces) | Year | Core sample | Potyvirus | Carmovirus | Sobemovirus | Poleroviruses | Cowpea tombusvirid 1 | Cowpea tombusvirid 2 | Cowpea tombusvirid 3 | Cowpea associated mycotymovirid 1 |
| BE1 | SS | Green mosaic | BF (Kadiogo) | 2013 | **nt** | **+/+** | **-/-** | **-/-** | **-/-** | **-/-** | **-/-** | **-/-** | **-/-** |
| BE2 | SS | Green mosaic | BF (Kadiogo) | 2013 | **nt** | **-/-** | **-/-** | **-/-** | **-/-** | **-/-** | **-/-** | **-/-** | **-/-** |
| BE3 | SS | Vein yellow mosaic | BF (Kadiogo) | 2013 | **nt** | **-/+** | **-/-** | **-/-** | **-/-** | **-/-** | **-/-** | **-/-** | **-/-** |
| BE4 | SS | Mosaic | BF (Kadiogo) | 2013 | **nt** | **+/+** | **-/-** | **-/-** | **-/-** | **-/-** | **-/-** | **-/-** | **-/-** |
| BE5 | SS | Green mosaic | BF (Kadiogo) | 2013 | **nt** | **+/+** | **-/-** | **-/-** | **-/-** | **-/-** | **-/-** | **-/-** | **-/-** |
| BE6 | SS | Yellow mosaic | BF (Kadiogo) | 2013 | **nt** | **+/+** | **-/-** | **-/-** | **-/-** | **-/-** | **-/-** | **-/-** | **-/-** |
| BE7 | SS | Asymptomatic | BF (Kadiogo) | 2013 | **nt** | **+/-** | **-/-** | **-/-** | **-/-** | **-/-** | **-/-** | **-/-** | **-/-** |
| BE8 | SS | Mosaic and leaf distorsion | BF (Kadiogo) | 2013 | **nt** | **+/+** | **-/-** | **-/-** | **-/-** | **-/-** | **-/-** | **-/-** | **-/-** |
| BE9 | SS | Leaf distorsion | BF (Kadiogo) | 2013 | **nt** | **+/+** | **-/-** | **-/-** | **-/-** | **-/-** | **-/-** | **-/-** | **-/-** |
| BE10 | SS | Leaf distorsion, vein chlorosis | BF (Kadiogo) | 2013 | **nt** | **-/-** | **-/-** | **-/-** | **-/-** | **-/-** | **-/-** | **-/-** | **-/-** |
| BE11 | SS | Mosaic | BF (Kadiogo) | 2013 | **nt** | **+/+** | **-/-** | **-/-** | **-/-** | **-/-** | **-/-** | **-/-** | **-/-** |
| BE12 | SS | Asymptomatic | BF (Kadiogo) | 2013 | **nt** | **-/-** | **-/-** | **-/-** | **-/-** | **-/-** | **-/-** | **-/-** | **-/-** |
| BE13 | SS | Asymptomatic | BF (Kadiogo) | 2013 | **nt** | **-/-** | **-/-** | **-/-** | **-/-** | **-/-** | **-/-** | **-/-** | **-/-** |
| BE14 | SS | Necrosis | BF (Kadiogo) | 2013 | **nt** | **+/-** | **-/-** | **-/-** | **-/-** | **-/-** | **-/-** | **-/-** | **-/-** |
| BE15 | SS | Asymptomatic | BF (Kadiogo) | 2013 | **nt** | **+/-** | **-/-** | **-/-** | **-/-** | **-/-** | **-/-** | **-/-** | **-/-** |
| BE16 | SS | Mosaic and stunt | BF (Kadiogo) | 2013 | **nt** | **+/-** | **-/-** | **-/-** | **-/-** | **-/-** | **-/-** | **-/-** | **-/-** |
| BE17 | SS | Asymptomatic | BF (Kadiogo) | 2013 | **nt** | **-/-** | **-/-** | **-/-** | **-/-** | **-/-** | **-/-** | **-/-** | **-/-** |
| BE18 | SS | Mosaic and stunt | BF (Kadiogo) | 2013 | **nt** | **+/+** | **-/-** | **-/-** | **-/-** | **-/-** | **-/-** | **-/-** | **-/-** |
| BE19 | SS | Asymptomatic | BF (Kadiogo) | 2013 | **nt** | **-/-** | **-/-** | **-/-** | **-/-** | **-/-** | **-/-** | **-/-** | **-/-** |
| BE20 | SS | Asymptomatic | BF (Kadiogo) | 2013 | **nt** | **-/-** | **-/-** | **-/-** | **-/-** | **-/-** | **-/-** | **-/-** | **-/-** |
| BE21 | SS | Asymptomatic | BF (Kadiogo) | 2013 | **nt** | **-/-** | **-/-** | **-/-** | **-/-** | **-/-** | **-/-** | **-/-** | **-/-** |
| BE22 | SS | Necrosis | BF (Kadiogo) | 2013 | **nt** | **-/-** | **-/-** | **-/-** | **-/-** | **-/-** | **-/-** | **-/-** | **-/-** |
| BE23 | SS | Asymptomatic | BF (Kadiogo) | 2013 | **nt** | **-/-** | **-/-** | **-/-** | **-/-** | **-/-** | **-/-** | **-/-** | **-/-** |
| BE24 | SS | Mosaic | BF (Kadiogo) | 2013 | **nt** | **+/+** | **-/-** | **-/-** | **-/-** | **-/-** | **-/-** | **-/-** | **-/-** |
| BE25 | SS | Mosaic | BF (Kadiogo) | 2013 | **nt** | **+/+** | **-/-** | **-/-** | **-/-** | **-/-** | **-/-** | **-/-** | **-/-** |
| BE26 | SS | Asymptomatic | BF (Kadiogo) | 2013 | **nt** | **-/-** | **-/-** | **-/-** | **-/-** | **-/-** | **-/-** | **-/-** | **-/-** |
| BE27 | SS | Mosaic and leaf distorsion | BF (Kadiogo) | 2013 | **nt** | **+/+** | **-/-** | **-/-** | **-/-** | **-/-** | **-/-** | **-/-** | **-/-** |
| BE28 | SS | Asymptomatic | BF (Kadiogo) | 2013 | **nt** | **+/-** | **-/-** | **-/-** | **-/-** | **-/-** | **-/-** | **-/-** | **-/-** |
| BE29 | SS | Yellow mosaic | BF (Kadiogo) | 2013 | **nt** | **+/+** | **-/-** | **-/-** | **-/-** | **-/-** | **-/-** | **-/-** | **-/-** |
| BE30 | SS | Asymptomatic | BF (Kadiogo) | 2013 | **nt** | **-/-** | **-/-** | **-/-** | **-/-** | **-/-** | **-/-** | **-/-** | **-/-** |
| BE31 | SS | Mosaic | BF (Passore) | 2013 | **nt** | **+/+** | **-/-** | **-/-** | **-/-** | **-/-** | **-/-** | **-/-** | **-/-** |
| BE32 | SS | Chlorosis and necrosis | BF (Passore) | 2013 | **nt** | **-/-** | **-/-** | **-/-** | **-/-** | **-/-** | **-/-** | **-/-** | **-/-** |
| BE33 | SS | Asymptomatic | BF (Passore) | 2013 | **nt** | **-/-** | **-/-** | **-/-** | **-/-** | **-/-** | **-/-** | **-/-** | **-/-** |
| BE34 | SS | Chlorosis | BF (Passore) | 2013 | **nt** | **-/-** | **-/-** | **-/-** | **-/-** | **-/-** | **-/-** | **-/-** | **-/-** |
| BE35 | SS | Mosaic and stunt | BF (Passore) | 2013 | **nt** | **+/+** | **-/-** | **-/-** | **-/-** | **-/-** | **-/-** | **-/-** | **-/-** |
| BE36 | SS | Mosaic | BF (Passore) | 2013 | **nt** | **+/+** | **-/-** | **-/-** | **-/-** | **-/-** | **-/-** | **-/-** | **-/-** |
| BE37 | SS | Mosaic | BF (Passore) | 2013 | **nt** | **+/+** | **-/-** | **-/-** | **-/-** | **-/-** | **-/-** | **-/-** | **-/-** |
| BE38 | SS | Mosaic | BF (Passore) | 2013 | **nt** | **+/-** | **-/-** | **-/-** | **-/-** | **-/-** | **-/-** | **-/-** | **-/-** |
| BE39 | SS | Asymptomatic | BF (Passore) | 2013 | **nt** | **+/-** | **-/-** | **-/-** | **-/-** | **-/-** | **-/-** | **-/-** | **-/-** |
| BE40 | SS | Yellow mosaic | BF (Passore) | 2013 | **nt** | **+/-** | **-/-** | **-/-** | **-/-** | **-/-** | **-/-** | **-/-** | **-/-** |
| BE41 | SS | Asymptomatic | BF (Passore) | 2013 | **nt** | **-/-** | **-/-** | **-/-** | **-/-** | **-/-** | **-/-** | **-/-** | **-/-** |
| BE42 | SS | Necrosis | BF (Passore) | 2013 | **nt** | **+/+** | **-/-** | **-/-** | **-/-** | **-/-** | **-/-** | **-/-** | **-/-** |
| BE43 | SS | Green mosaic | BF (Passore) | 2013 | **nt** | **-/-** | **-/-** | **-/-** | **-/-** | **-/-** | **-/-** | **-/-** | **-/-** |
| BE44 | SS | Yellow mosaic | BF (Passore) | 2013 | **nt** | **+/+** | **-/-** | **-/-** | **-/-** | **-/-** | **-/-** | **-/-** | **-/-** |
| BE45 | SS | Mosaic | BF (Passore) | 2013 | **nt** | **+/+** | **-/-** | **-/-** | **-/-** | **-/-** | **-/-** | **-/-** | **-/-** |
| BE46 | SS | Asymptomatic | BF (Passore) | 2013 | **nt** | **+/-** | **-/-** | **-/-** | **-/-** | **-/-** | **-/-** | **-/-** | **-/-** |
| BE47 | SS | Green mosaic | BF (Passore) | 2013 | **nt** | **+/+** | **-/-** | **-/-** | **-/-** | **-/-** | **-/-** | **-/-** | **-/-** |
| BE48 | SS | Asymptomatic | BF (Passore) | 2013 | **nt** | **+/-** | **-/-** | **-/-** | **-/-** | **-/-** | **-/-** | **-/-** | **-/-** |
| BE49 | SS | Mosaic | BF (Passore) | 2013 | **nt** | **+/+** | **-/-** | **-/-** | **-/-** | **-/-** | **-/-** | **-/-** | **-/-** |
| BE50 | SS | Yellow mosaic | BF (Passore) | 2013 | **nt** | **+/+** | **-/-** | **-/-** | **-/-** | **-/-** | **-/-** | **-/-** | **-/-** |
| BE51 | Sahel | Mosaic | BF (Yatenga) | 2013 | **nt** | **+/+** | **-/-** | **-/-** | **-/-** | **-/-** | **-/-** | **-/-** | **-/-** |
| BE52 | Sahel | Yellowing and necrosis | BF (Yatenga) | 2013 | **nt** | **+/-** | **-/-** | **-/-** | **-/-** | **-/-** | **-/-** | **-/-** | **-/-** |
| BE53 | Sahel | Leaf distorsion | BF (Yatenga) | 2013 | **nt** | **+/+** | **-/-** | **-/-** | **-/-** | **-/-** | **-/-** | **-/-** | **-/-** |
| BE54 | Sahel | Asymptomatic | BF (Yatenga) | 2013 | **nt** | **-/-** | **-/-** | **-/-** | **-/-** | **-/-** | **-/-** | **-/-** | **-/-** |
| BE55 | Sahel | Asymptomatic | BF (Yatenga) | 2013 | **nt** | **-/-** | **-/-** | **-/-** | **-/-** | **-/-** | **-/-** | **-/-** | **-/-** |
| BE56 | Sahel | Mosaic | BF (Yatenga) | 2013 | **nt** | **+/+** | **-/-** | **-/-** | **-/-** | **-/-** | **-/-** | **-/-** | **-/-** |
| BE57 | Sahel | Severe mosaic | BF (Yatenga) | 2013 | **nt** | **+/+** | **-/-** | **-/-** | **-/-** | **-/-** | **-/-** | **-/-** | **-/-** |
| BE58 | Sahel | Mosaic and leaf distorsion | BF (Yatenga) | 2013 | **nt** | **+/+** | **-/-** | **-/-** | **-/-** | **-/-** | **-/-** | **-/-** | **-/-** |
| BE59 | Sahel | Asymptomatic | BF (Yatenga) | 2013 | **nt** | **+/+** | **-/-** | **-/-** | **-/-** | **-/-** | **-/-** | **-/-** | **-/-** |
| BE60 | Sahel | Chlorosis | BF (Yatenga) | 2013 | **nt** | **+/-** | **-/-** | **-/-** | **-/-** | **-/-** | **-/-** | **-/-** | **-/-** |
| BE61 | Sahel | Asymptomatic | BF (Yatenga) | 2013 | **nt** | **-/-** | **-/-** | **-/-** | **-/-** | **-/-** | **-/-** | **-/-** | **-/-** |
| BE62 | Sahel | Asymptomatic | BF (Yatenga) | 2013 | **nt** | **-/-** | **-/-** | **-/-** | **-/-** | **-/-** | **-/-** | **-/-** | **-/-** |
| BE63 | Sahel | Asymptomatic | BF (Yatenga) | 2013 | **nt** | **-/-** | **-/-** | **-/-** | **-/-** | **-/-** | **-/-** | **-/-** | **-/-** |
| BE64 | Sahel | Asymptomatic | BF (Yatenga) | 2013 | **nt** | **-/-** | **-/-** | **-/-** | **-/-** | **-/-** | **-/-** | **-/-** | **-/-** |
| BE65 | Sahel | Asymptomatic | BF (Yatenga) | 2013 | **nt** | **-/-** | **-/-** | **-/-** | **-/-** | **-/-** | **-/-** | **-/-** | **-/-** |
| BE66 | Sahel | Asymptomatic | BF (Yatenga) | 2013 | **nt** | **-/-** | **-/-** | **-/-** | **-/-** | **-/-** | **-/-** | **-/-** | **-/-** |
| BE67 | Sahel | Asymptomatic | BF (Yatenga) | 2013 | **nt** | **+/-** | **-/-** | **-/-** | **-/-** | **-/-** | **-/-** | **-/-** | **-/-** |
| BE68 | Sahel | Asymptomatic | BF (Yatenga) | 2013 | **nt** | **-/-** | **-/-** | **-/-** | **-/-** | **-/-** | **-/-** | **-/-** | **-/-** |
| BE69 | Sahel | Asymptomatic | BF (Yatenga) | 2013 | **nt** | **+/-** | **-/-** | **-/-** | **-/-** | **-/-** | **-/-** | **-/-** | **-/-** |
| BE70 | Sahel | Asymptomatic | BF (Yatenga) | 2013 | **nt** | **-/-** | **-/-** | **-/-** | **-/-** | **-/-** | **-/-** | **-/-** | **-/-** |
| BE71 | Sahel | Asymptomatic | BF (Soum) | 2013 | **nt** | **-/-** | **-/-** | **-/-** | **-/-** | **-/-** | **-/-** | **-/-** | **-/-** |
| BE72 | Sahel | Mild mosaic and necrosis | BF (Soum) | 2013 | **nt** | **-/-** | **-/-** | **-/-** | **-/-** | **-/-** | **-/-** | **-/-** | **-/-** |
| BE73 | Sahel | Yellow mosaic | BF (Soum) | 2013 | **nt** | **-/-** | **-/-** | **-/-** | **-/-** | **-/-** | **-/-** | **-/-** | **-/-** |
| BE74 | Sahel | Yellow mosaic | BF (Soum) | 2013 | **nt** | **-/-** | **-/-** | **-/-** | **-/-** | **-/-** | **-/-** | **-/-** | **-/-** |
| BE75 | Sahel | Asymptomatic | BF (Soum) | 2013 | **nt** | **-/-** | **-/-** | **-/-** | **-/-** | **-/-** | **-/-** | **-/-** | **-/-** |
| BE76 | Sahel | Leaf distorsion | BF (Soum) | 2013 | **nt** | **-/-** | **-/-** | **-/-** | **-/-** | **-/-** | **-/-** | **-/-** | **-/-** |
| BE77 | Sahel | Asymptomatic | BF (Soum) | 2013 | **nt** | **+/-** | **-/-** | **-/-** | **-/-** | **-/-** | **-/-** | **-/-** | **-/-** |
| BE78 | Sahel | Asymptomatic | BF (Soum) | 2013 | **nt** | **+/-** | **-/-** | **-/-** | **-/-** | **-/-** | **-/-** | **-/-** | **-/-** |
| BE79 | Sahel | Necrosis | BF (Soum) | 2013 | **nt** | **-/-** | **-/-** | **-/-** | **-/-** | **-/-** | **-/-** | **-/-** | **-/-** |
| BE80 | Sahel | Necrosis | BF (Soum) | 2013 | **nt** | **-/-** | **-/-** | **-/-** | **-/-** | **-/-** | **-/-** | **-/-** | **-/-** |
| BE80' | Sahel | Necrosis | BF (Soum) | 2013 | **nt** | **-/-** | **-/-** | **-/-** | **-/-** | **-/-** | **-/-** | **-/-** | **-/-** |
| BE81 | Sahel | Leaf distorsion | BF (Soum) | 2013 | **x** | **-/-** | **-/-** | **-/-** | **-/-** | **+/+** | **-/-** | **-/-** | **-/-** |
| BE82 | Sahel | Necrosis | BF (Soum) | 2013 | **x** | **-/-** | **-/-** | **-/-** | **-/-** | **-/-** | **-/-** | **-/-** | **-/-** |
| BE83 | Sahel | Asymptomatic | BF (Soum) | 2013 | **x** | **-/-** | **-/-** | **-/-** | **-/-** | **-/-** | **-/-** | **-/-** | **-/-** |
| BE84 | Sahel | Asymptomatic | BF (Soum) | 2013 | **nt** | **+/-** | **-/-** | **-/-** | **-/-** | **-/-** | **-/-** | **-/-** | **-/-** |
| BE85 | Sahel | Necrosis | BF (Soum) | 2013 | **nt** | **-/-** | **-/-** | **-/-** | **-/-** | **-/-** | **-/-** | **-/-** | **-/-** |
| BE86 | Sahel | Necrosis | BF (Soum) | 2013 | **nt** | **-/-** | **-/-** | **-/-** | **-/-** | **-/-** | **-/-** | **-/-** | **-/-** |
| BE87 | Sahel | Asymptomatic | BF (Soum) | 2013 | **nt** | **-/-** | **-/-** | **-/-** | **-/-** | **-/-** | **-/-** | **-/-** | **-/-** |
| BE88 | Sahel | Leaf distorsion | BF (Soum) | 2013 | **nt** | **-/-** | **-/-** | **-/-** | **-/-** | **-/-** | **-/-** | **-/-** | **-/-** |
| BE89 | Sahel | Chlorosis | BF (Soum) | 2013 | **nt** | **+/-** | **-/-** | **-/-** | **-/-** | **-/-** | **-/-** | **-/-** | **-/-** |
| BE90 | Sahel | Chlorosis | BF (Soum) | 2013 | **nt** | **+/+** | **-/-** | **-/-** | **-/-** | **-/-** | **-/-** | **-/-** | **-/-** |
| BE91 | Sahel | Leaf distorsion | BF (Soum) | 2013 | **nt** | **-/-** | **-/-** | **-/-** | **-/-** | **-/-** | **-/-** | **-/-** | **-/-** |
| BE92 | Sahel | Asymptomatic | BF (Soum) | 2013 | **nt** | **-/-** | **-/-** | **-/-** | **-/-** | **-/-** | **-/-** | **-/-** | **-/-** |
| BE93 | Sahel | Chlorosis | BF (Soum) | 2013 | **nt** | **-/-** | **-/-** | **-/-** | **-/-** | **-/-** | **-/-** | **-/-** | **-/-** |
| BE94 | Sahel | Leaf distorsion | BF (Soum) | 2013 | **nt** | **-/-** | **-/-** | **-/-** | **-/-** | **-/-** | **-/-** | **-/-** | **-/-** |
| BE95 | Sahel | Chlorosis | BF (Soum) | 2013 | **x** | **-/-** | **-/-** | **-/-** | **-/-** | **-/-** | **-/-** | **-/-** | **-/-** |
| BE96 | Sahel | Leaf distorsion | BF (Soum) | 2013 | **nt** | **nd/-** | **nd/-** | **nd/-** | **nd/-** | **nd/-** | **nd/-** | **nd/-** | **nd/-** |
| BE97 | Sahel | Leaf distorsion | BF (Soum) | 2013 | **nt** | **-/-** | **-/-** | **-/-** | **-/-** | **-/-** | **-/-** | **-/-** | **-/-** |
| BE98 | Sahel | Mosaic | BF (Seno) | 2013 | **x** | **-/-** | **-/-** | **-/-** | **-/-** | **-/-** | **-/-** | **-/-** | **-/-** |
| BE99 | Sahel | Necrosis | BF (Seno) | 2013 | **nt** | **-/-** | **-/-** | **-/-** | **-/-** | **-/-** | **-/-** | **-/-** | **-/-** |
| BE100 | Sahel | Chlorosis | BF (Seno) | 2013 | **nt** | **-/-** | **-/-** | **-/-** | **-/-** | **-/-** | **-/-** | **-/-** | **-/-** |
| BE101 | Sahel | Asymptomatic | BF (Seno) | 2013 | **nt** | **-/-** | **-/-** | **-/-** | **-/-** | **-/-** | **-/-** | **-/-** | **-/-** |
| BE102 | Sahel | Necrosis | BF (Seno) | 2013 | **nt** | **-/-** | **-/-** | **-/-** | **-/-** | **-/-** | **-/-** | **-/-** | **-/-** |
| BE103 | Sahel | Asymptomatic | BF (Seno) | 2013 | **nt** | **-/-** | **-/-** | **-/-** | **-/-** | **-/-** | **-/-** | **-/-** | **-/-** |
| BE104 | Sahel | Necrosis | BF (Seno) | 2013 | **nt** | **-/-** | **-/-** | **-/-** | **-/-** | **-/-** | **-/-** | **-/-** | **-/-** |
| BE105 | Sahel | Leaf distorsion | BF (Seno) | 2013 | **nt** | **-/-** | **-/-** | **-/-** | **-/-** | **-/-** | **-/-** | **-/-** | **-/-** |
| BE106 | Sahel | Necrosis | BF (Seno) | 2013 | **nt** | **-/-** | **-/-** | **-/-** | **-/-** | **-/-** | **-/-** | **-/-** | **-/-** |
| BE107 | Sahel | Leaf distorsion | BF (Seno) | 2013 | **nt** | **-/-** | **-/-** | **-/-** | **-/-** | **-/-** | **-/-** | **-/-** | **-/-** |
| BE108 | Sahel | Asymptomatic | BF (Seno) | 2013 | **nt** | **-/-** | **-/-** | **-/-** | **-/-** | **-/-** | **-/-** | **-/-** | **-/-** |
| BE109 | Sahel | Leaf distorsion | BF (Seno) | 2013 | **nt** | **-/-** | **-/-** | **-/-** | **-/-** | **-/-** | **-/-** | **-/-** | **-/-** |
| BE110 | Sahel | Asymptomatic | BF (Seno) | 2013 | **nt** | **-/-** | **-/-** | **-/-** | **-/-** | **-/-** | **-/-** | **-/-** | **-/-** |
| BE111 | Sahel | Necrosis | BF (Seno) | 2013 | **nt** | **-/-** | **-/-** | **-/-** | **-/-** | **-/-** | **-/-** | **-/-** | **-/-** |
| BE112 | Sahel | Asymptomatic | BF (Seno) | 2013 | **x** | **-/-** | **-/-** | **-/-** | **-/-** | **-/-** | **-/-** | **-/-** | **-/-** |
| BE113 | Sahel | Chlorosis | BF (Seno) | 2013 | **nt** | **-/-** | **-/-** | **-/-** | **-/-** | **-/-** | **-/-** | **-/-** | **-/-** |
| BE114 | Sahel | Asymptomatic | BF (Seno) | 2013 | **nt** | **-/-** | **-/-** | **-/-** | **-/-** | **-/-** | **-/-** | **-/-** | **-/-** |
| BE115 | Sahel | Asymptomatic | BF (Seno) | 2013 | **x** | **-/-** | **-/-** | **-/-** | **-/-** | **-/-** | **-/-** | **-/-** | **-/-** |
| BE116 | SS | Mild mosaic | BF (Sanmatenga) | 2013 | **x** | **+/+** | **-/-** | **-/-** | **-/-** | **-/-** | **-/-** | **-/-** | **-/-** |
| BE117 | SS | Mosaic | BF (Sanmatenga) | 2013 | **x** | **+/+** | **-/-** | **-/-** | **-/-** | **-/-** | **-/-** | **-/-** | **-/-** |
| BE118 | SS | Asymptomatic | BF (Sanmatenga) | 2013 | **nt** | **-/-** | **-/-** | **-/-** | **-/-** | **-/-** | **-/-** | **-/-** | **-/-** |
| BE119 | SS | Necrosis | BF (Sanmatenga) | 2013 | **x** | **+/+** | **-/-** | **-/-** | **-/-** | **-/-** | **-/-** | **-/-** | **-/-** |
| BE120 | SS | Mosaic | BF (Sanmatenga) | 2013 | **x** | **+/+** | **-/-** | **-/-** | **-/-** | **-/-** | **-/-** | **-/-** | **+/+** |
| BE121 | SS | Mosaic | BF (Sanmatenga) | 2013 | **x** | **-/+** | **-/-** | **-/-** | **-/-** | **-/-** | **-/-** | **-/-** | **-/+** |
| BE122 | SS | Mosaic | BF (Sanmatenga) | 2013 | **nt** | **+/+** | **-/-** | **-/-** | **-/-** | **-/-** | **-/-** | **-/-** | **-/-** |
| BE123 | SS | Green mosaic | BF (Sanmatenga) | 2013 | **nt** | **+/+** | **-/-** | **-/-** | **-/-** | **-/-** | **-/-** | **-/-** | **-/-** |
| BE124 | SS | Asymptomatic | BF (Sanmatenga) | 2013 | **nt** | **-/-** | **-/-** | **-/-** | **-/-** | **-/-** | **-/-** | **-/-** | **-/-** |
| BE125 | SS | Leaf distorsion and necrosis | BF (Sanmatenga) | 2013 | **nt** | **-/-** | **-/-** | **-/-** | **-/-** | **-/-** | **-/-** | **-/-** | **-/-** |
| BE126 | SS | Mosaic | BF (Sanmatenga) | 2013 | **nt** | **+/-** | **-/-** | **-/-** | **-/-** | **-/-** | **-/-** | **-/-** | **-/-** |
| BE127 | SS | Chlorosis | BF (Sanmatenga) | 2013 | **nt** | **-/-** | **-/-** | **-/-** | **-/-** | **-/-** | **-/-** | **-/-** | **-/-** |
| BE128 | SS | Asymptomatic | BF (Sanmatenga) | 2013 | **nt** | **-/-** | **-/-** | **-/-** | **-/-** | **-/-** | **-/-** | **-/-** | **-/-** |
| BE129 | SS | Chlorosis | BF (Sanmatenga) | 2013 | **nt** | **-/-** | **-/-** | **-/-** | **-/-** | **-/-** | **-/-** | **-/-** | **-/-** |
| BE130 | SS | Mosaic | BF (Sanmatenga) | 2013 | **nt** | **-/-** | **-/-** | **-/-** | **-/-** | **-/-** | **-/-** | **-/-** | **-/-** |
| BE131 | SS | Mosaic | BF (Sanmatenga) | 2013 | **nt** | **-/-** | **-/-** | **-/-** | **-/-** | **-/-** | **-/-** | **-/-** | **-/-** |
| BE132 | SS | Yellow mosaic | BF (Sanmatenga) | 2013 | **nt** | **+/-** | **-/-** | **-/-** | **-/-** | **-/-** | **-/-** | **-/-** | **-/-** |
| BE133 | SS | Mosaic | BF (Sanmatenga) | 2013 | **nt** | **+/-** | **-/-** | **-/-** | **-/-** | **-/-** | **-/-** | **-/-** | **-/-** |
| BE134 | SS | Mosaic and leaf distorsion | BF (Sanmatenga) | 2013 | **nt** | **-/-** | **-/-** | **-/-** | **-/-** | **-/-** | **-/-** | **-/-** | **-/-** |
| BE135 | SS | Green mosaic | BF (Sanmatenga) | 2013 | **nt** | **+/-** | **-/-** | **-/-** | **-/-** | **-/-** | **-/-** | **-/-** | **-/-** |
| BE136 | SS | Green mosaic | BF (Sanmatenga) | 2013 | **nt** | **+/-** | **-/-** | **-/-** | **-/-** | **-/-** | **-/-** | **-/-** | **-/-** |
| BE137 | SS | Mosaic | BF (Sanmatenga) | 2013 | **x** | **+/+** | **-/-** | **-/-** | **-/-** | **-/+** | **-/-** | **+/+** | **-/-** |
| BE138 | SS | Yellow mosaic | BF (Sanmatenga) | 2013 | **x** | **+/+** | **-/-** | **-/-** | **-/-** | **-/-** | **-/-** | **-/-** | **-/-** |
| BE139 | SS | Yellow mosaic | BF (Sanmatenga) | 2013 | **x** | **+/+** | **-/-** | **-/-** | **-/-** | **-/-** | **-/-** | **-/-** | **-/-** |
| BE140 | SS | Asymptomatic | BF (Sanmatenga) | 2013 | **nt** | **+/+** | **-/-** | **-/-** | **-/-** | **-/-** | **-/-** | **-/-** | **-/-** |
| BE141 | SS | Mosaic and leaf distorsion | BF (Sanmatenga) | 2013 | **nt** | **+/+** | **-/-** | **-/-** | **-/-** | **-/-** | **-/-** | **-/-** | **-/-** |
| BE142 | SS | Mosaic and necrosis | BF (Sanmatenga) | 2013 | **nt** | **-/+** | **-/-** | **-/-** | **-/-** | **-/-** | **-/-** | **-/-** | **-/-** |
| BE143 | SS | Mosaic | BF (Sanmatenga) | 2013 | **nt** | **+/+** | **-/-** | **-/-** | **-/-** | **-/-** | **-/-** | **-/-** | **-/-** |
| BE144 | SS | Mosaic | BF (Sanmatenga) | 2013 | **nt** | **+/+** | **-/-** | **-/-** | **-/-** | **-/-** | **-/-** | **-/-** | **-/-** |
| BE145 | SS | Mild mosaic | BF (Sanmatenga) | 2013 | **nt** | **-/-** | **-/-** | **-/-** | **-/-** | **-/-** | **-/-** | **-/-** | **-/-** |
| BE146 | SS | Mosaic | BF (Sanmatenga) | 2013 | **nt** | **+/+** | **-/-** | **-/-** | **-/-** | **-/-** | **-/-** | **-/-** | **-/-** |
| BE147 | SS | Necrosis | BF (Oubritenga) | 2013 | **nt** | **-/-** | **-/-** | **-/-** | **-/-** | **-/-** | **-/-** | **-/-** | **-/-** |
| BE148 | SS | Asymptomatic | BF (Oubritenga) | 2013 | **nt** | **-/-** | **-/-** | **-/-** | **-/-** | **-/-** | **-/-** | **-/-** | **-/-** |
| BE149 | SS | Mosaic | BF (Oubritenga) | 2013 | **nt** | **+/+** | **-/-** | **-/-** | **-/-** | **-/-** | **-/-** | **-/-** | **-/-** |
| BE150 | SS | Yellowing | BF (Oubritenga) | 2013 | **nt** | **+/+** | **-/-** | **-/-** | **-/-** | **-/-** | **-/-** | **-/-** | **-/-** |
| BE151 | SS | Yellow mosaic | BF (Oubritenga) | 2013 | **nt** | **+/+** | **-/-** | **-/-** | **-/-** | **-/-** | **-/-** | **-/-** | **-/-** |
| BE152 | SS | Mosaic | BF (Oubritenga) | 2013 | **nt** | **+/+** | **-/-** | **-/-** | **-/-** | **-/-** | **-/-** | **-/-** | **-/-** |
| BE153 | SS | Green mosaic | BF (Oubritenga) | 2013 | **nt** | **+/+** | **-/-** | **-/-** | **-/-** | **-/-** | **-/-** | **-/-** | **-/-** |
| BE154 | SS | Asymptomatic | BF (Oubritenga) | 2013 | **nt** | **-/-** | **-/-** | **-/-** | **-/-** | **-/-** | **-/-** | **-/-** | **-/-** |
| BE155 | SS | Green mosaic | BF (Oubritenga) | 2013 | **nt** | **+/+** | **-/-** | **-/-** | **-/-** | **-/-** | **-/-** | **-/-** | **-/-** |
| BE156 | SS | Mosaic and necrosis | BF (Oubritenga) | 2013 | **nt** | **+/+** | **-/-** | **-/-** | **-/-** | **-/-** | **-/-** | **-/-** | **-/-** |
| BE157 | SS | Mild mosaic | BF (Oubritenga) | 2013 | **nt** | **+/-** | **-/-** | **-/-** | **-/-** | **-/-** | **-/-** | **-/-** | **-/-** |
| BE158 | SS | Mosaic | BF (Oubritenga) | 2013 | **x** | **+/+** | **-/-** | **-/-** | **-/-** | **-/-** | **+/+** | **-/-** | **-/+** |
| BE159 | SS | Green mosaic | BF (Oubritenga) | 2013 | **x** | **+/+** | **-/-** | **-/-** | **-/-** | **-/-** | **-/-** | **-/-** | **-/-** |
| BE160 | SS | Mosaic | BF (Oubritenga) | 2013 | **x** | **+/+** | **-/-** | **-/-** | **-/-** | **-/-** | **-/-** | **-/-** | **-/-** |
| BE161 | SS | Asymptomatic | BF (Oubritenga) | 2013 | **nt** | **-/-** | **-/-** | **-/-** | **-/-** | **-/-** | **-/-** | **-/-** | **-/-** |
| BE162 | SS | Severe mosaic | BF (Oubritenga) | 2013 | **x** | **+/+** | **-/-** | **-/-** | **-/-** | **-/-** | **-/-** | **-/-** | **-/-** |
| BE163 | SS | Mosaic | BF (Oubritenga) | 2013 | **nt** | **+/+** | **-/-** | **-/-** | **-/-** | **-/-** | **-/-** | **-/-** | **-/-** |
| BE164 | SS | Mosaic | BF (Oubritenga) | 2013 | **nt** | **+/+** | **-/-** | **-/-** | **-/-** | **-/-** | **-/-** | **-/-** | **-/-** |
| BE165 | SS | Mosaic | BF (Oubritenga) | 2013 | **nt** | **+/+** | **-/-** | **-/-** | **-/-** | **-/-** | **-/-** | **-/-** | **-/-** |
| BE166 | SS | Asymptomatic | BF (Oubritenga) | 2013 | **nt** | **-/-** | **-/-** | **-/-** | **-/-** | **-/-** | **-/-** | **-/-** | **-/-** |
| BE167 | SS | Yellow mosaic | BF (Gourma) | 2013 | **x** | **+/+** | **-/-** | **-/-** | **+/+** | **-/-** | **-/-** | **-/-** | **-/-** |
| BE168 | SS | Yellowing following vein | BF (Gourma) | 2013 | **x** | **+/+** | **-/-** | **-/-** | **-/+** | **-/-** | **-/-** | **-/-** | **-/-** |
| BE169 | SS | Mosaic | BF (Gourma) | 2013 | **x** | **+/+** | **-/-** | **-/-** | **+/+** | **-/-** | **-/-** | **-/-** | **-/-** |
| BE170 | SS | Interveinal yellowing | BF (Gourma) | 2013 | **x** | **+/+** | **-/-** | **-/-** | **+/+** | **-/-** | **-/-** | **-/-** | **-/-** |
| BE171 | SS | Mosaic and leaf distorsion | BF (Gourma) | 2013 | **nt** | **+/+** | **-/-** | **-/-** | **-/-** | **-/-** | **-/-** | **-/-** | **-/-** |
| BE172 | SS | Mosaic and leaf distorsion | BF (Gourma) | 2013 | **x** | **+/+** | **-/-** | **-/-** | **+/+** | **-/-** | **-/-** | **-/-** | **-/-** |
| BE173 | SS | Yellowing | BF (Gourma) | 2013 | **x** | **+/+** | **-/-** | **-/-** | **+/+** | **-/-** | **-/-** | **-/-** | **-/-** |
| BE174 | SS | Mosaic and necrosis | BF (Gourma) | 2013 | **nt** | **+/+** | **-/-** | **-/-** | **-/-** | **-/-** | **-/-** | **-/-** | **-/-** |
| BE175 | SS | Mild mosaic | BF (Gourma) | 2013 | **nt** | **+/+** | **-/-** | **-/-** | **-/-** | **-/-** | **-/-** | **-/-** | **-/-** |
| BE176 | SS | Mosaic | BF (Gourma) | 2013 | **nt** | **nd/-** | **nd/nt** | **nd/nt** | **nd/nt** | **nd/nt** | **nd/nt** | **nd/nt** | **nd/nt** |
| BE177 | SS | Chlorosis | BF (Gourma) | 2013 | **nt** | **+/+** | **-/-** | **-/-** | **-/-** | **-/-** | **-/-** | **-/-** | **-/-** |
| BE178 | SS | Mosaic and leaf distorsion | BF (Gourma) | 2013 | **nt** | **+/+** | **-/-** | **-/-** | **-/-** | **-/-** | **-/-** | **-/-** | **-/-** |
| BE179 | SS | Mild yellow mosaic | BF (Gourma) | 2013 | **x** | **+/+** | **-/-** | **-/-** | **+/+** | **-/-** | **-/-** | **-/-** | **-/-** |
| BE180 | SS | Mosaic and leaf distorsion | BF (Gourma) | 2013 | **x** | **+/+** | **-/-** | **-/-** | **-/-** | **-/-** | **-/-** | **-/-** | **-/-** |
| BE181 | SS | Mosaic and leaf distorsion | BF (Gourma) | 2013 | **nt** | **+/+** | **-/-** | **-/-** | **-/-** | **-/-** | **-/-** | **-/-** | **-/-** |
| BE182 | SS | Chlorosis | BF (Gourma) | 2013 | **nt** | **-/-** | **-/-** | **-/-** | **-/-** | **-/-** | **-/-** | **-/-** | **-/-** |
| BE183 | SS | Asymptomatic | BF (Gourma) | 2013 | **nt** | **-/-** | **-/-** | **-/-** | **-/-** | **-/-** | **-/-** | **-/-** | **-/-** |
| BE184 | SS | Mosaic | BF (Gourma) | 2013 | **nt** | **-/-** | **-/-** | **-/-** | **-/-** | **-/-** | **-/-** | **-/-** | **-/-** |
| BE185 | SS | Mosaic and leaf distorsion | BF (Gourma) | 2013 | **nt** | **-/-** | **-/-** | **-/-** | **-/-** | **-/-** | **-/-** | **-/-** | **-/-** |
| BE186 | SS | Mosaic | BF (Gourma) | 2013 | **x** | **+/+** | **-/-** | **-/-** | **+/-** | **-/-** | **-/-** | **-/-** | **-/-** |
| BE187 | SS | Mosaic | BF (Gourma) | 2013 | **x** | **+/+** | **-/-** | **-/-** | **-/-** | **-/-** | **-/-** | **-/-** | **-/+** |
| BE188 | SS | Mosaic | BF (Boulgou) | 2013 | **nt** | **+/+** | **-/-** | **-/-** | **-/-** | **-/-** | **-/-** | **-/-** | **-/-** |
| BE189 | SS | Green mosaic | BF (Boulgou) | 2013 | **nt** | **+/+** | **-/-** | **-/-** | **-/-** | **-/-** | **-/-** | **-/-** | **-/-** |
| BE190 | SS | Mild yellow mosaic | BF (Boulgou) | 2013 | **x** | **+/+** | **-/-** | **-/-** | **+/+** | **-/+** | **-/-** | **-/-** | **-/+** |
| BE191 | SS | Mosaic and stunt | BF (Boulgou) | 2013 | **x** | **+/+** | **-/-** | **-/-** | **-/-** | **-/-** | **-/-** | **-/-** | **-/-** |
| BE192 | SS | Green mosaic | BF (Boulgou) | 2013 | **nt** | **+/+** | **-/-** | **-/-** | **-/-** | **-/-** | **-/-** | **-/-** | **-/-** |
| BE193 | SS | Green mosaic | BF (Boulgou) | 2013 | **nt** | **+/-** | **-/-** | **-/-** | **-/-** | **-/-** | **-/-** | **-/-** | **-/-** |
| BE194 | SS | Leaf distorsion | BF (Boulgou) | 2013 | **nt** | **+/-** | **-/-** | **-/-** | **-/-** | **-/-** | **-/-** | **-/-** | **-/-** |
| BE195 | SS | Yellow severe mosaic | BF (Boulgou) | 2013 | **nt** | **+/-** | **-/-** | **-/-** | **-/-** | **-/-** | **-/-** | **-/-** | **-/-** |
| BE196 | SS | Green mosaic | BF (Boulgou) | 2013 | **nt** | **+/+** | **-/-** | **-/-** | **-/-** | **-/-** | **-/-** | **-/-** | **-/-** |
| BE197 | SS | Yellow mosaic | BF (Boulgou) | 2013 | **x** | **+/+** | **-/-** | **-/-** | **+/+** | **-/+** | **-/-** | **-/-** | **-/-** |
| BE198 | SS | Green mosaic | BF (Boulgou) | 2013 | **x** | **+/+** | **-/-** | **-/-** | **-/-** | **-/-** | **-/-** | **-/-** | **-/-** |
| BE199 | SS | Severe mosaic ant stunt | BF (Boulgou) | 2013 | **x** | **+/+** | **-/-** | **-/-** | **-/-** | **-/-** | **-/-** | **-/-** | **-/-** |
| BE200 | SS | Yellow mosaic | BF (Boulgou) | 2013 | **nt** | **+/+** | **-/-** | **-/-** | **-/-** | **-/-** | **-/-** | **-/-** | **-/-** |
| BE201 | SS | Yellow mosaic | BF (Boulgou) | 2013 | **nt** | **+/+** | **-/-** | **-/-** | **-/-** | **-/-** | **-/-** | **-/-** | **-/-** |
| BE202 | SS | Yellow mosaic | BF (Boulgou) | 2013 | **nt** | **+/+** | **-/-** | **-/-** | **-/-** | **-/-** | **-/-** | **-/-** | **-/-** |
| BE203 | SS | Green mosaic | BF (Boulgou) | 2013 | **nt** | **+/-** | **-/-** | **-/-** | **-/-** | **-/-** | **-/-** | **-/-** | **-/-** |
| BE204 | SS | Leaf distorsion | BF (Boulgou) | 2013 | **nt** | **-/-** | **-/-** | **-/-** | **-/-** | **-/-** | **-/-** | **-/-** | **-/-** |
| BE205 | SS | Leaf distorsion | BF (Boulgou) | 2013 | **nt** | **-/-** | **-/-** | **-/-** | **-/-** | **-/-** | **-/-** | **-/-** | **-/-** |
| BE206 | SS | Mosaic and leaf distorsion | BF (Boulgou) | 2013 | **nt** | **-/+** | **-/-** | **-/-** | **-/-** | **-/-** | **-/-** | **-/-** | **-/-** |
| BE207 | SS | Green mosaic | BF (Boulgou) | 2013 | **nt** | **+/+** | **-/-** | **-/-** | **-/-** | **-/-** | **-/-** | **-/-** | **-/-** |
| BE208 | Soudan | Yellow mosaic | BF (Houet) | 2013 | **nt** | **nd/-** | **nd/nt** | **nd/nt** | **nd/nt** | **nd/nt** | **nd/nt** | **nd/nt** | **nd/nt** |
| BE209 | Soudan | Green mosaic | BF (Houet) | 2013 | **nt** | **+/-** | **-/-** | **-/-** | **-/-** | **-/-** | **-/-** | **-/-** | **-/-** |
| BE210 | Soudan | Chlorosis | BF (Houet) | 2013 | **nt** | **+/-** | **-/-** | **-/-** | **-/-** | **-/-** | **-/-** | **-/-** | **-/-** |
| BE211 | Soudan | Chlorosis | BF (Houet) | 2013 | **nt** | **-/-** | **-/-** | **-/-** | **-/-** | **-/-** | **-/-** | **-/-** | **-/-** |
| BE212 | Soudan | Vein yellow mosaic | BF (Houet) | 2013 | **nt** | **+/+** | **-/-** | **-/-** | **-/-** | **-/-** | **-/-** | **-/-** | **-/-** |
| BE213 | Soudan | Mosaic and leaf distorsion | BF (Houet) | 2013 | **nt** | **+/-** | **-/-** | **-/-** | **-/-** | **-/-** | **-/-** | **-/-** | **-/-** |
| BE214 | Soudan | Mild mosaic | BF (Houet) | 2013 | **nt** | **+/+** | **-/-** | **-/-** | **-/-** | **-/-** | **-/-** | **-/-** | **-/-** |
| BE215 | Soudan | Mosaic and stunt | BF (Houet) | 2013 | **nt** | **-/-** | **-/-** | **-/-** | **-/-** | **-/-** | **-/-** | **-/-** | **-/-** |
| BE216 | Soudan | Chlorosis | BF (Houet) | 2013 | **nt** | **-/-** | **-/-** | **-/-** | **-/-** | **-/-** | **-/-** | **-/-** | **-/-** |
| BE217 | Soudan | Chlorosis | BF (Houet) | 2013 | **nt** | **-/-** | **-/-** | **-/-** | **-/-** | **-/-** | **-/-** | **-/-** | **-/-** |
| BE218 | Soudan | Mosaic | BF (Houet) | 2013 | **nt** | **+/+** | **-/-** | **-/-** | **-/-** | **-/-** | **-/-** | **-/-** | **-/-** |
| BE219 | Soudan | Leaf distorsion | BF (Houet) | 2013 | **nt** | **+/+** | **-/-** | **-/-** | **-/-** | **-/-** | **-/-** | **-/-** | **-/-** |
| BE220 | Soudan | Mosaic | BF (Houet) | 2013 | **nt** | **nd/+** | **nd/nt** | **nd/nt** | **nd/nt** | **nd/nt** | **nd/nt** | **nd/nt** | **nd/nt** |
| BE221 | Soudan | Green mosaic | BF (Houet) | 2013 | **nt** | **+/-** | **-/-** | **-/-** | **-/-** | **-/-** | **-/-** | **-/-** | **-/-** |
| BE222 | Soudan | Mosaic | BF (Houet) | 2013 | **nt** | **+/+** | **-/-** | **-/-** | **-/-** | **-/-** | **-/-** | **-/-** | **-/-** |
| BE223 | Soudan | Yellow mosaic | BF (Houet) | 2013 | **nt** | **+/+** | **-/-** | **-/-** | **-/-** | **-/-** | **-/-** | **-/-** | **-/-** |
| BE224 | Soudan | Mosaic and leaf distorsion | BF (Houet) | 2013 | **nt** | **+/+** | **-/-** | **-/-** | **-/-** | **-/-** | **-/-** | **-/-** | **-/-** |
| BE225 | Soudan | Yellow mosaic | BF (Houet) | 2013 | **nt** | **+/+** | **-/-** | **-/-** | **-/-** | **-/-** | **-/-** | **-/-** | **-/-** |
| BE226 | Soudan | Chlorosis | BF (Houet) | 2013 | **nt** | **+/+** | **-/-** | **-/-** | **-/-** | **-/-** | **-/-** | **-/-** | **-/-** |
| BE227 | Soudan | Asymptomatic | BF (Houet) | 2013 | **nt** | **+/+** | **-/-** | **-/-** | **-/-** | **-/-** | **-/-** | **-/-** | **-/-** |
| BE228 | Soudan | Mosaic | BF (Kenedougou) | 2013 | **nt** | **+/-** | **-/-** | **-/-** | **-/-** | **-/-** | **-/-** | **-/-** | **-/-** |
| BE229 | Soudan | Asymptomatic | BF (Kenedougou) | 2013 | **nt** | **+/-** | **-/-** | **-/-** | **-/-** | **-/-** | **-/-** | **-/-** | **-/-** |
| BE230 | Soudan | Mosaic | BF (Kenedougou) | 2013 | **nt** | **+/+** | **-/-** | **-/-** | **-/-** | **-/-** | **-/-** | **-/-** | **-/-** |
| BE231 | Soudan | Mosaic | BF (Kenedougou) | 2013 | **nt** | **+/-** | **-/-** | **-/-** | **-/-** | **-/-** | **-/-** | **-/-** | **-/-** |
| BE232 | Soudan | Mosaic and stunt | BF (Kenedougou) | 2013 | **nt** | **+/-** | **-/-** | **-/-** | **-/-** | **-/-** | **-/-** | **-/-** | **-/-** |
| BE233 | Soudan | Yellow severe mosaic | BF (Kenedougou) | 2013 | **nt** | **+/+** | **-/-** | **-/-** | **-/-** | **-/-** | **-/-** | **-/-** | **-/-** |
| BE234 | Soudan | Yellow mosaic | BF (Kenedougou) | 2013 | **nt** | **+/+** | **-/-** | **-/-** | **-/-** | **-/-** | **-/-** | **-/-** | **-/-** |
| BE235 | Soudan | Mosaic and leaf distorsion | BF (Kenedougou) | 2013 | **nt** | **+/+** | **-/-** | **-/-** | **-/-** | **-/-** | **-/-** | **-/-** | **-/-** |
| BE236 | Soudan | Green mosaic | BF (Kenedougou) | 2013 | **nt** | **+/+** | **-/-** | **-/-** | **-/-** | **-/-** | **-/-** | **-/-** | **-/-** |
| BE237 | Soudan | Yellow mosaic | BF (Kenedougou) | 2013 | **nt** | **+/+** | **-/-** | **-/-** | **-/-** | **-/-** | **-/-** | **-/-** | **-/-** |
| BE238 | Soudan | Mosaic | BF (Kenedougou) | 2013 | **nt** | **+/+** | **-/-** | **-/-** | **-/-** | **-/-** | **-/-** | **-/-** | **-/-** |
| BE239 | Soudan | Mosaic | BF (Kenedougou) | 2013 | **nt** | **+/-** | **-/-** | **-/-** | **-/-** | **-/-** | **-/-** | **-/-** | **-/-** |
| BE240 | Soudan | Green mosaic | BF (Kenedougou) | 2013 | **nt** | **+/+** | **-/-** | **-/-** | **-/-** | **-/-** | **-/-** | **-/-** | **-/-** |
| BE241 | Soudan | Mosaic | BF (Kenedougou) | 2013 | **nt** | **+/+** | **-/-** | **-/-** | **-/-** | **-/-** | **-/-** | **-/-** | **-/-** |
| BE242 | Soudan | Necrosis | BF (Kenedougou) | 2013 | **nt** | **-/-** | **-/-** | **-/-** | **-/-** | **-/-** | **-/-** | **-/-** | **-/-** |
| BE243 | Soudan | Mosaic | BF (Kenedougou) | 2013 | **nt** | **+/-** | **-/-** | **-/-** | **-/-** | **-/-** | **-/-** | **-/-** | **-/-** |
| BE244 | Soudan | Mosaic and stunt | BF (Kenedougou) | 2013 | **nt** | **+/-** | **-/-** | **-/-** | **-/-** | **-/-** | **-/-** | **-/-** | **-/-** |
| BE245 | Soudan | Asymptomatic | BF (Kenedougou) | 2013 | **nt** | **-/+** | **-/-** | **-/-** | **-/-** | **-/-** | **-/-** | **-/-** | **-/-** |
| BE246 | Soudan | Mosaic | BF (Kenedougou) | 2013 | **nt** | **+/+** | **-/-** | **-/-** | **-/-** | **-/-** | **-/-** | **-/-** | **-/-** |
| BE247 | Soudan | Mosaic and leaf distorsion | BF (Kenedougou) | 2013 | **nt** | **+/-** | **-/-** | **-/-** | **-/-** | **-/-** | **-/-** | **-/-** | **-/-** |
| BE248 | Soudan | Mosaic | BF (Kenedougou) | 2013 | **nt** | **-/+** | **-/-** | **-/-** | **-/-** | **-/-** | **-/-** | **-/-** | **-/-** |
| BE249 | Soudan | Chlorosis | BF (Comoe) | 2013 | **nt** | **-/-** | **-/-** | **-/-** | **-/-** | **-/-** | **-/-** | **-/-** | **-/-** |
| BE250 | Soudan | Mild mosaic | BF (Comoe) | 2013 | **x** | **-/-** | **-/-** | **+/+** | **-/-** | **-/-** | **-/-** | **-/-** | **-/-** |
| BE251 | Soudan | Mosaic | BF (Comoe) | 2013 | **x** | **+/+** | **-/-** | **-/-** | **-/-** | **-/-** | **-/-** | **-/-** | **-/-** |
| BE252 | Soudan | Mosaic | BF (Comoe) | 2013 | **x** | **+/+** | **-/-** | **+/+** | **-/-** | **-/-** | **-/-** | **-/-** | **-/-** |
| BE253 | Soudan | Mosaic | BF (Comoe) | 2013 | **nt** | **+/+** | **-/-** | **-/-** | **-/-** | **-/-** | **-/-** | **-/-** | **-/-** |
| BE254 | Soudan | Mosaic | BF (Comoe) | 2013 | **x** | **+/+** | **-/-** | **-/-** | **-/-** | **-/-** | **-/-** | **-/-** | **-/-** |
| BE255 | Soudan | Mosaic | BF (Comoe) | 2013 | **nt** | **+/-** | **-/-** | **-/-** | **-/-** | **-/-** | **-/-** | **-/-** | **-/-** |
| BE256 | Soudan | Severe mosaic | BF (Comoe) | 2013 | **nt** | **+/+** | **-/-** | **-/-** | **-/-** | **-/-** | **-/-** | **-/-** | **-/-** |
| BE257 | Soudan | Mosaic | BF (Comoe) | 2013 | **nt** | **+/+** | **-/-** | **-/-** | **-/-** | **-/-** | **-/-** | **-/-** | **-/-** |
| BE258 | Soudan | Mosaic | BF (Comoe) | 2013 | **nt** | **+/+** | **-/-** | **-/-** | **-/-** | **-/-** | **-/-** | **-/-** | **-/-** |
| BE259 | Soudan | Mosaic | BF (Comoe) | 2013 | **nt** | **+/+** | **-/-** | **-/-** | **-/-** | **-/-** | **-/-** | **-/-** | **-/-** |
| BE260 | Soudan | Mosaic | BF (Comoe) | 2013 | **nt** | **+/-** | **-/-** | **-/-** | **-/-** | **-/-** | **-/-** | **-/-** | **-/-** |
| BE261 | Soudan | Mosaic | BF (Comoe) | 2013 | **nt** | **+/-** | **-/-** | **-/-** | **-/-** | **-/-** | **-/-** | **-/-** | **-/-** |
| BE262 | Soudan | Yellow mosaic | BF (Comoe) | 2013 | **nt** | **+/+** | **-/-** | **-/-** | **-/-** | **-/-** | **-/-** | **-/-** | **-/-** |
| BE263 | Soudan | Green mosaic | BF (Comoe) | 2013 | **nt** | **+/+** | **-/-** | **-/-** | **-/-** | **-/-** | **-/-** | **-/-** | **-/-** |
| BE264 | Soudan | Mosaic and leaf distorsion | BF (Comoe) | 2013 | **nt** | **+/+** | **-/-** | **-/-** | **-/-** | **-/-** | **-/-** | **-/-** | **-/-** |
| BE265 | Soudan | Mosaic | BF (Comoe) | 2013 | **nt** | **+/+** | **-/-** | **-/-** | **-/-** | **-/-** | **-/-** | **-/-** | **-/-** |
| BE266 | Soudan | Yellow mosaic | BF (Comoe) | 2013 | **nt** | **+/+** | **-/-** | **-/-** | **-/-** | **-/-** | **-/-** | **-/-** | **-/-** |
| BE267 | Soudan | Mosaic and leaf distorsion | BF (Comoe) | 2013 | **nt** | **+/+** | **-/-** | **-/-** | **-/-** | **-/-** | **-/-** | **-/-** | **-/-** |
| BE268 | Soudan | Severe mosaic | BF (Comoe) | 2013 | **nt** | **+/+** | **-/-** | **-/-** | **-/-** | **-/-** | **-/-** | **-/-** | **-/-** |
| BE269 | Soudan | Mosaic | BF (Comoe) | 2013 | **nt** | **+/+** | **-/-** | **-/-** | **-/-** | **-/-** | **-/-** | **-/-** | **-/-** |
| BE270 | Soudan | Mosaic | BF (Poni) | 2013 | **nt** | **-/-** | **-/-** | **-/-** | **-/-** | **-/-** | **-/-** | **-/-** | **-/-** |
| BE271 | Soudan | Mosaic | BF (Poni) | 2013 | **nt** | **+/+** | **-/-** | **-/-** | **-/-** | **-/-** | **-/-** | **-/-** | **-/-** |
| BE272 | Soudan | Mild mosaic | BF (Poni) | 2013 | **nt** | **-/-** | **-/-** | **-/-** | **-/-** | **-/-** | **-/-** | **-/-** | **-/-** |
| BE273 | Soudan | Mosaic and necrosis | BF (Poni) | 2013 | **x** | **+/+** | **+/+** | **+/+** | **-/-** | **-/-** | **-/-** | **-/-** | **-/-** |
| BE274 | Soudan | Mosaic | BF (Poni) | 2013 | **x** | **+/+** | **-/+** | **-/-** | **-/-** | **-/-** | **-/-** | **-/-** | **-/-** |
| BE275 | Soudan | Mild mosaic | BF (Poni) | 2013 | **x** | **+/+** | **-/+** | **-/-** | **-/-** | **-/-** | **-/-** | **-/-** | **-/-** |
| BE276 | Soudan | Mosaic | BF (Poni) | 2013 | **x** | **+/+** | **+/+** | **-/-** | **-/-** | **-/-** | **-/-** | **-/-** | **-/-** |
| BE277 | Soudan | Asymptomatic | BF (Poni) | 2013 | **nt** | **+/-** | **-/-** | **-/-** | **-/-** | **-/-** | **-/-** | **-/-** | **-/-** |
| BE278 | Soudan | Mosaic and necrosis | BF (Poni) | 2013 | **x** | **+/+** | **-/-** | **-/-** | **-/-** | **-/-** | **-/-** | **-/-** | **-/-** |
| BE279 | Soudan | Mosaic | BF (Poni) | 2013 | **x** | **+/+** | **-/-** | **-/-** | **+/+** | **-/-** | **-/-** | **-/-** | **-/-** |
| BE280 | Soudan | Mild mosaic | BF (Poni) | 2013 | **nt** | **+/+** | **-/-** | **-/-** | **-/-** | **-/-** | **-/-** | **-/-** | **-/-** |
| BE281 | Soudan | Mosaic | BF (Poni) | 2013 | **nt** | **+/-** | **-/-** | **-/-** | **-/-** | **-/-** | **-/-** | **-/-** | **-/-** |
| BE282 | Soudan | Green mosaic | BF (Poni) | 2013 | **nt** | **+/-** | **-/-** | **-/-** | **-/-** | **-/-** | **-/-** | **-/-** | **-/-** |
| BE283 | Soudan | Yellow mosaic | BF (Poni) | 2013 | **nt** | **+/+** | **-/-** | **-/-** | **-/-** | **-/-** | **-/-** | **-/-** | **-/-** |
| BE284 | Soudan | Severe mosaic | BF (Poni) | 2013 | **nt** | **+/+** | **-/-** | **-/-** | **-/-** | **-/-** | **-/-** | **-/-** | **-/-** |
| BE285 | Soudan | Yellow mosaic | BF (Poni) | 2013 | **nt** | **+/-** | **-/-** | **-/-** | **-/-** | **-/-** | **-/-** | **-/-** | **-/-** |
| BE286 | Soudan | Yellow mosaic | BF (Poni) | 2013 | **x** | **+/+** | **-/-** | **-/-** | **-/-** | **-/-** | **-/-** | **-/-** | **-/-** |
| BE287 | Soudan | Yellow mosaic | BF (Poni) | 2013 | **x** | **+/+** | **+/-** | **-/-** | **-/-** | **-/-** | **-/-** | **-/-** | **-/-** |
| BE288 | Soudan | Yellow mosaic | BF (Poni) | 2013 | **x** | **+/+** | **-/-** | **-/-** | **-/-** | **-/-** | **-/-** | **-/-** | **-/-** |
| BE289 | Soudan | Mosaic and leaf distorsion | BF (Poni) | 2013 | **nt** | **+/-** | **-/-** | **-/-** | **-/-** | **-/-** | **-/-** | **-/-** | **-/-** |
| BE290 | Soudan | Mosaic | BF (Bougouriba) | 2013 | **nt** | **+/+** | **-/-** | **-/-** | **-/-** | **-/-** | **-/-** | **-/-** | **-/-** |
| BE291 | Soudan | Mosaic | BF (Bougouriba) | 2013 | **nt** | **+/-** | **-/-** | **-/-** | **-/-** | **-/-** | **-/-** | **-/-** | **-/-** |
| BE292 | Soudan | Mosaic and leaf distorsion | BF (Bougouriba) | 2013 | **nt** | **+/+** | **-/-** | **-/-** | **-/-** | **-/-** | **-/-** | **-/-** | **-/-** |
| BE293 | Soudan | Mosaic | BF (Bougouriba) | 2013 | **nt** | **+/+** | **-/-** | **-/-** | **-/-** | **-/-** | **-/-** | **-/-** | **-/-** |
| BE294 | Soudan | Yellow mosaic | BF (Bougouriba) | 2013 | **nt** | **+/+** | **-/-** | **-/-** | **-/-** | **-/-** | **-/-** | **-/-** | **-/-** |
| BE295 | Soudan | Mosaic | BF (Bougouriba) | 2013 | **nt** | **+/+** | **-/-** | **-/-** | **-/-** | **-/-** | **-/-** | **-/-** | **-/-** |
| BE296 | Soudan | Mosaic and leaf distorsion | BF (Bougouriba) | 2013 | **nt** | **+/+** | **-/-** | **-/-** | **-/-** | **-/-** | **-/-** | **-/-** | **-/-** |
| BE297 | Soudan | Severe mosaic | BF (Bougouriba) | 2013 | **nt** | **+/+** | **-/-** | **-/-** | **-/-** | **-/-** | **-/-** | **-/-** | **-/-** |
| BE298 | Soudan | Severe mosaic | BF (Bougouriba) | 2013 | **nt** | **+/-** | **-/-** | **-/-** | **-/-** | **-/-** | **-/-** | **-/-** | **-/-** |
| BE299 | Soudan | Mosaic | BF (Bougouriba) | 2013 | **nt** | **+/+** | **-/-** | **-/-** | **-/-** | **-/-** | **-/-** | **-/-** | **-/-** |
| BE300 | Soudan | Yellow mosaic | BF (Bougouriba) | 2013 | **nt** | **+/+** | **-/-** | **-/-** | **-/-** | **-/-** | **-/-** | **-/-** | **-/-** |
| BE301 | Soudan | Severe mosaic | BF (Bougouriba) | 2013 | **nt** | **+/-** | **-/-** | **-/-** | **-/-** | **-/-** | **-/-** | **-/-** | **-/-** |
| BE302 | Soudan | Severe mosaic ant stunt | BF (Bougouriba) | 2013 | **nt** | **+/+** | **-/-** | **-/-** | **-/-** | **-/-** | **-/-** | **-/-** | **-/-** |
| BE303 | Soudan | Mosaic | BF (Bougouriba) | 2013 | **nt** | **+/+** | **-/-** | **-/-** | **-/-** | **-/-** | **-/-** | **-/-** | **-/-** |
| BE304 | Soudan | Mosaic and leaf distorsion | BF (Bougouriba) | 2013 | **nt** | **nd/+** | **nd/nt** | **nd/nt** | **nd/nt** | **nd/nt** | **nd/nt** | **nd/nt** | **nd/nt** |
| BE305 | Soudan | Asymptomatic | BF (Bougouriba) | 2013 | **nt** | **-/-** | **-/-** | **-/-** | **-/-** | **-/-** | **-/-** | **-/-** | **-/-** |
| BE306 | Soudan | Asymptomatic | BF (Bougouriba) | 2013 | **nt** | **+/-** | **-/-** | **-/-** | **-/-** | **-/-** | **-/-** | **-/-** | **-/-** |
| BE307 | Soudan | Severe mosaic | BF (Bougouriba) | 2013 | **nt** | **-/-** | **-/-** | **-/-** | **-/-** | **-/-** | **-/-** | **-/-** | **-/-** |
| BE308 | Soudan | Yellow mosaic | BF (Bougouriba) | 2013 | **nt** | **+/-** | **-/-** | **-/-** | **-/-** | **-/-** | **-/-** | **-/-** | **-/-** |
| BE309 | Soudan | Asymptomatic | BF (Bougouriba) | 2013 | **nt** | **-/-** | **-/-** | **-/-** | **-/-** | **-/-** | **-/-** | **-/-** | **-/-** |
| BE310 | Soudan | Mosaic | BF (Bougouriba) | 2013 | **nt** | **+/-** | **-/-** | **-/-** | **-/-** | **-/-** | **-/-** | **-/-** | **-/-** |
| BE311 | Soudan | Mosaic | BF (Bougouriba) | 2013 | **nt** | **+/+** | **-/-** | **-/-** | **-/-** | **-/-** | **-/-** | **-/-** | **-/-** |
| A1_Kom |  | russet-red | BF | 2014 | **nt** | **-/nt** | **-/nt** | **-/nt** | **-/nt** | **-/nt** | **-/nt** | **-/nt** | **-/nt** |
| A3_Kom |  | russet-red | BF | 2014 | **nt** | **-/nt** | **-/nt** | **-/nt** | **-/nt** | **-/nt** | **-/nt** | **-/nt** | **-/nt** |
| A9_Kom |  | russet-red | BF | 2014 | **nt** | **-/nt** | **-/nt** | **-/nt** | **-/nt** | **-/nt** | **-/nt** | **-/nt** | **-/nt** |
| C6_Kom |  | russet-red | BF | 2014 | **nt** | **-/nt** | **-/nt** | **-/nt** | **-/nt** | **-/nt** | **-/nt** | **-/nt** | **-/nt** |
| L3_Kom |  | russet-red | BF | 2014 | **nt** | **-/nt** | **-/nt** | **-/nt** | **-/nt** | **-/nt** | **-/nt** | **-/nt** | **-/nt** |
| K6_Kom |  | Embossing | BF | 2014 | **nt** | **-/nt** | **-/nt** | **-/nt** | **-/nt** | **-/nt** | **-/nt** | **-/nt** | **-/nt** |
| D1_Kom |  | Embossing | BF | 2014 | **nt** | **-/nt** | **-/nt** | **-/nt** | **-/nt** | **-/nt** | **-/nt** | **-/nt** | **-/nt** |
| D4_Kom |  | Asymptomatic | BF | 2014 | **nt** | **-/nt** | **-/nt** | **-/nt** | **-/nt** | **-/nt** | **-/nt** | **-/nt** | **-/nt** |
| A2_Naf |  | russet-red and mosaic | BF | 2014 | **x** | **+/+** | **-/-** | **-/-** | **-/-** | **-/-** | **-/-** | **-/-** | **-/-** |
| B2_Naf |  | Embossing | BF | 2014 | **x** | **+/-** | **-/-** | **-/-** | **-/-** | **-/-** | **-/-** | **-/-** | **-/-** |
| E1_Naf |  | russet-red | BF | 2014 | **nt** | **-/nt** | **-/nt** | **-/nt** | **-/nt** | **-/nt** | **-/nt** | **-/nt** | **-/nt** |
| E6_Naf |  | Asymptomatic | BF | 2014 | **nt** | **-/nt** | **-/nt** | **-/nt** | **-/nt** | **-/nt** | **-/nt** | **-/nt** | **-/nt** |
| I2_Naf |  | russet-red | BF | 2014 | **nt** | **-/nt** | **-/nt** | **-/nt** | **-/nt** | **-/nt** | **-/nt** | **-/nt** | **-/nt** |
| I9_Naf |  | russet-red | BF | 2014 | **nt** | **-/nt** | **-/nt** | **-/nt** | **-/nt** | **-/nt** | **-/nt** | **-/nt** | **-/nt** |
| C2_Naf |  | Embossing | BF | 2014 | **nt** | **-/nt** | **-/nt** | **-/nt** | **-/nt** | **-/nt** | **-/nt** | **-/nt** | **-/nt** |
| B6_Naf |  | russet-red | BF | 2014 | **nt** | **-/nt** | **-/nt** | **-/nt** | **-/nt** | **-/nt** | **-/nt** | **-/nt** | **-/nt** |
| B7_Tilg |  | russet-red | BF | 2014 | **nt** | **-/.** | **-/.** | **-/.** | **-/.** | **-/.** | **-/.** | **-/.** | **-/.** |
| B7_Tilg |  | russet-red | BF | 2014 | **nt** | **-/nt** | **-/nt** | **-/nt** | **-/nt** | **-/nt** | **-/nt** | **-/nt** | **-/nt** |
| D1_Tilg |  | russet-red | BF | 2014 | **nt** | **-/nt** | **-/nt** | **-/nt** | **-/nt** | **-/nt** | **-/nt** | **-/nt** | **-/nt** |
| D2_Tilg |  | russet-red and mosaic | BF | 2014 | **x** | **+/-** | **-/-** | **-/-** | **-/-** | **-/-** | **-/-** | **-/-** | **-/-** |
| D6_Tilg |  | russet-red | BF | 2014 | **nt** | **-/nt** | **-/nt** | **-/nt** | **-/nt** | **-/nt** | **-/nt** | **-/nt** | **-/nt** |
| I9_Tilg |  | Embossing | BF | 2014 | **nt** | **-/nt** | **-/nt** | **-/nt** | **-/nt** | **-/nt** | **-/nt** | **-/nt** | **-/nt** |
| L9_Tilg |  | Embossing | BF | 2014 | **nt** | **-/nt** | **-/nt** | **-/nt** | **-/nt** | **-/nt** | **-/nt** | **-/nt** | **-/nt** |
| L3_Tilg |  | Asymptomatic | BF | 2014 | **nt** | **-/nt** | **-/nt** | **-/nt** | **-/nt** | **-/nt** | **-/nt** | **-/nt** | **-/nt** |
| K9_Tilg |  | Asymptomatic | BF | 2014 | **nt** | **-/nt** | **-/nt** | **-/nt** | **-/nt** | **-/nt** | **-/nt** | **-/nt** | **-/nt** |
| L8_Gorg |  | Asymptomatic | BF | 2014 | **nt** | **-/nt** | **-/nt** | **-/nt** | **-/nt** | **-/nt** | **-/nt** | **-/nt** | **-/nt** |
| I8_Gorg |  | Asymptomatic | BF | 2014 | **nt** | **-/nt** | **-/nt** | **-/nt** | **-/nt** | **-/nt** | **-/nt** | **-/nt** | **-/nt** |
| A5_Gorg |  | Asymptomatic | BF | 2014 | **nt** | **-/nt** | **-/nt** | **-/nt** | **-/nt** | **-/nt** | **-/nt** | **-/nt** | **-/nt** |
| E8_Gorg |  | Asymptomatic | BF | 2014 | **nt** | **-/nt** | **-/nt** | **-/nt** | **-/nt** | **-/nt** | **-/nt** | **-/nt** | **-/nt** |
| I5_Gorg |  | Embossing | BF | 2014 | **nt** | **-/nt** | **-/nt** | **-/nt** | **-/nt** | **-/nt** | **-/nt** | **-/nt** | **-/nt** |
| E9_Gorg |  | Embossing | BF | 2014 | **nt** | **-/nt** | **-/nt** | **-/nt** | **-/nt** | **-/nt** | **-/nt** | **-/nt** | **-/nt** |
| A1_Gorg |  | Asymptomatic | BF | 2014 | **nt** | **-/nt** | **-/nt** | **-/nt** | **-/nt** | **-/nt** | **-/nt** | **-/nt** | **-/nt** |
| D1_Gorg |  | Asymptomatic | BF | 2014 | **nt** | **-/nt** | **-/nt** | **-/nt** | **-/nt** | **-/nt** | **-/nt** | **-/nt** | **-/nt** |
| K5_Niz |  | Asymptomatic | BF | 2014 | **nt** | **-/nt** | **-/nt** | **-/nt** | **-/nt** | **-/nt** | **-/nt** | **-/nt** | **-/nt** |
| K9_Niz |  | Asymptomatic | BF | 2014 | **nt** | **-/nt** | **-/nt** | **-/nt** | **-/nt** | **-/nt** | **-/nt** | **-/nt** | **-/nt** |
| I9_Niz |  | Asymptomatic | BF | 2014 | **nt** | **-/nt** | **-/nt** | **-/nt** | **-/nt** | **-/nt** | **-/nt** | **-/nt** | **-/nt** |
| J4_Niz |  | Asymptomatic | BF | 2014 | **nt** | **-/nt** | **-/nt** | **-/nt** | **-/nt** | **-/nt** | **-/nt** | **-/nt** | **-/nt** |
| C4_Niz |  | Embossing | BF | 2014 | **nt** | **-/nt** | **-/nt** | **-/nt** | **-/nt** | **-/nt** | **-/nt** | **-/nt** | **-/nt** |
| D7_Niz |  | Embossing | BF | 2014 | **nt** | **-/nt** | **-/nt** | **-/nt** | **-/nt** | **-/nt** | **-/nt** | **-/nt** | **-/nt** |
| D4_Niz |  | Asymptomatic | BF | 2014 | **nt** | **-/nt** | **-/nt** | **-/nt** | **-/nt** | **-/nt** | **-/nt** | **-/nt** | **-/nt** |
| G7_Niz |  | Asymptomatic | BF | 2014 | **nt** | **-/nt** | **-/nt** | **-/nt** | **-/nt** | **-/nt** | **-/nt** | **-/nt** | **-/nt** |
| K1_Yiss |  | Mosaic | BF | 2014 | **x** | **+/-** | **-/-** | **-/-** | **-/-** | **-/-** | **-/-** | **-/-** | **-/-** |
| E8_Yiss |  | Asymptomatic | BF | 2014 | **nt** | **-/nt** | **-/nt** | **-/nt** | **-/nt** | **-/nt** | **-/nt** | **-/nt** | **-/nt** |
| B5_Yiss |  | Mosaic | BF | 2014 | **nt** | **-/nt** | **-/nt** | **-/nt** | **-/nt** | **-/nt** | **-/nt** | **-/nt** | **-/nt** |
| L8_Yiss |  | Asymptomatic | BF | 2014 | **nt** | **-/nt** | **-/nt** | **-/nt** | **-/nt** | **-/nt** | **-/nt** | **-/nt** | **-/nt** |
| J3_Yiss |  | Embossing | BF | 2014 | **nt** | **-/nt** | **-/nt** | **-/nt** | **-/nt** | **-/nt** | **-/nt** | **-/nt** | **-/nt** |
| I8_Yiss |  | Embossing | BF | 2014 | **nt** | **-/nt** | **-/nt** | **-/nt** | **-/nt** | **-/nt** | **-/nt** | **-/nt** | **-/nt** |
| D2_Yiss |  | Asymptomatic | BF | 2014 | **nt** | **-/nt** | **-/nt** | **-/nt** | **-/nt** | **-/nt** | **-/nt** | **-/nt** | **-/nt** |
| J2_Yiss |  | Asymptomatic | BF | 2014 | **nt** | **-/nt** | **-/nt** | **-/nt** | **-/nt** | **-/nt** | **-/nt** | **-/nt** | **-/nt** |
| A5_Kvx_61-1 |  | Asymptomatic | BF | 2014 | **nt** | **-/nt** | **-/nt** | **-/nt** | **-/nt** | **-/nt** | **-/nt** | **-/nt** | **-/nt** |
| B9_Kvx_61-1 |  | Asymptomatic | BF | 2014 | **nt** | **-/nt** | **-/nt** | **-/nt** | **-/nt** | **-/nt** | **-/nt** | **-/nt** | **-/nt** |
| E2_Kvx_61-1 |  | russet-red | BF | 2014 | **nt** | **-/nt** | **-/nt** | **-/nt** | **-/nt** | **-/nt** | **-/nt** | **-/nt** | **-/nt** |
| I3_Kvx_61-1 |  | Embossing | BF | 2014 | **nt** | **-/nt** | **-/nt** | **-/nt** | **-/nt** | **-/nt** | **-/nt** | **-/nt** | **-/nt** |
| J3_Kvx_61-1 |  | Mosaic | BF | 2014 | **nt** | **-/nt** | **-/nt** | **-/nt** | **-/nt** | **-/nt** | **-/nt** | **-/nt** | **-/nt** |
| L4_Kvx_61-1 |  | Embossing | BF | 2014 | **nt** | **-/nt** | **-/nt** | **-/nt** | **-/nt** | **-/nt** | **-/nt** | **-/nt** | **-/nt** |
| E9_Kvx_61-1 |  | Asymptomatic | BF | 2014 | **x** | **+/-** | **-/-** | **-/-** | **-/-** | **-/-** | **-/-** | **-/-** | **-/-** |
| D8_Kvx_61-1 |  | Asymptomatic | BF | 2014 | **nt** | **-/nt** | **-/nt** | **-/nt** | **-/nt** | **-/nt** | **-/nt** | **-/nt** | **-/nt** |
| A1_Moussa |  | Asymptomatic | BF | 2014 | **nt** | **-/nt** | **-/nt** | **-/nt** | **-/nt** | **-/nt** | **-/nt** | **-/nt** | **-/nt** |
| D9_Moussa |  | Asymptomatic | BF | 2014 | **nt** | **-/nt** | **-/nt** | **-/nt** | **-/nt** | **-/nt** | **-/nt** | **-/nt** | **-/nt** |
| G5_Moussa |  | russet-red | BF | 2014 | **nt** | **-/nt** | **-/nt** | **-/nt** | **-/nt** | **-/nt** | **-/nt** | **-/nt** | **-/nt** |
| J4_Moussa |  | russet-red | BF | 2014 | **nt** | **-/nt** | **-/nt** | **-/nt** | **-/nt** | **-/nt** | **-/nt** | **-/nt** | **-/nt** |
| J2_Moussa |  | Embossing | BF | 2014 | **nt** | **-/nt** | **-/nt** | **-/nt** | **-/nt** | **-/nt** | **-/nt** | **-/nt** | **-/nt** |
| L1_Moussa |  | Embossing | BF | 2014 | **nt** | **-/nt** | **-/nt** | **-/nt** | **-/nt** | **-/nt** | **-/nt** | **-/nt** | **-/nt** |
| G1_Moussa |  | Asymptomatic | BF | 2014 | **nt** | **-/nt** | **-/nt** | **-/nt** | **-/nt** | **-/nt** | **-/nt** | **-/nt** | **-/nt** |
| D5_Moussa |  | Asymptomatic | BF | 2014 | **nt** | **-/nt** | **-/nt** | **-/nt** | **-/nt** | **-/nt** | **-/nt** | **-/nt** | **-/nt** |
| TG1 | Togo | Mosaic | Togo | 2014 | **nt** | **+/nt** | **-/nt** | **-/nt** | **-/nt** | **-/nt** | **-/nt** | **-/nt** | **-/nt** |
| TG2 | Togo | Mosaic | Togo | 2014 | **nt** | **+/nt** | **-/nt** | **-/nt** | **-/nt** | **-/nt** | **-/nt** | **-/nt** | **-/nt** |
| TG3 | Togo | Mosaic | Togo | 2014 | **nt** | **+/nt** | **-/nt** | **-/nt** | **-/nt** | **-/nt** | **-/nt** | **-/nt** | **-/nt** |
| TG4 | Togo | Mosaic | Togo | 2014 | **nt** | **+/nt** | **-/nt** | **-/nt** | **-/nt** | **-/nt** | **-/nt** | **-/nt** | **-/nt** |
| TG5 | Togo | Mosaic | Togo | 2014 | **nt** | **+/nt** | **-/nt** | **-/nt** | **-/nt** | **-/nt** | **-/nt** | **-/nt** | **-/nt** |
| TG6 | Togo | Mosaic | Togo | 2014 | **nt** | **+/nt** | **-/nt** | **-/nt** | **-/nt** | **-/nt** | **-/nt** | **-/nt** | **-/nt** |
| TG7 | Togo | Mosaic | Togo | 2014 | **nt** | **+/nt** | **-/nt** | **-/nt** | **-/nt** | **-/nt** | **-/nt** | **-/nt** | **-/nt** |
| BE1-14 | SS | nd | BF (Gourma) | 2014 | **nt** | **nt/+** | **nt/-** | **nt/-** | **nt/-** | **nt/-** | **nt/-** | **nt/-** | **nt/-** |
| BE2/14 | SS | nd | BF (Gourma) | 2014 | **nt** | **nt/+** | **nt/-** | **nt/-** | **nt/-** | **nt/-** | **nt/-** | **nt/-** | **nt/-** |
| BE3/14 | SS | nd | BF (Gourma) | 2014 | **nt** | **nt/-** | **nt/-** | **nt/-** | **nt/-** | **nt/-** | **nt/-** | **nt/-** | **nt/-** |
| BE4/14 | SS | nd | BF (Gourma) | 2014 | **nt** | **nt/+** | **nt/-** | **nt/-** | **nt/-** | **nt/-** | **nt/-** | **nt/-** | **nt/-** |
| BE5/14 | SS | nd | BF (Gourma) | 2014 | **nt** | **nt/+** | **nt/-** | **nt/-** | **nt/-** | **nt/-** | **nt/-** | **nt/-** | **nt/-** |
| BE6/14 | SS | nd | BF (Gourma) | 2014 | **nt** | **nt/nt** | **nt/-** | **nt/-** | **nt/-** | **nt/-** | **nt/-** | **nt/-** | **nt/-** |
| BE7/14 | SS | nd | BF (Gourma) | 2014 | **nt** | **nt/nt** | **nt/-** | **nt/-** | **nt/-** | **nt/-** | **nt/-** | **nt/-** | **nt/-** |
| BE8/14 | SS | nd | BF (Gourma) | 2014 | **nt** | **nt/nt** | **nt/-** | **nt/-** | **nt/-** | **nt/-** | **nt/-** | **nt/-** | **nt/-** |
| BE9/14 | SS | nd | BF (Gourma) | 2014 | **nt** | **nt/nt** | **nt/-** | **nt/-** | **nt/-** | **nt/-** | **nt/-** | **nt/-** | **nt/-** |
| BE10/14 | SS | nd | BF (Gourma) | 2014 | **nt** | **nt/+** | **nt/-** | **nt/-** | **nt/-** | **nt/-** | **nt/-** | **nt/-** | **nt/-** |
| BE11/14 | SS | nd | BF (Boulgou) | 2014 | **nt** | **nt/+** | **nt/-** | **nt/-** | **nt/-** | **nt/-** | **nt/-** | **nt/-** | **nt/-** |
| BE12/14 | SS | nd | BF (Boulgou) | 2014 | **nt** | **nt/+** | **nt/-** | **nt/-** | **nt/-** | **nt/-** | **nt/-** | **nt/-** | **nt/-** |
| BE13/14 | SS | nd | BF (Boulgou) | 2014 | **nt** | **nt/+** | **nt/-** | **nt/-** | **nt/-** | **nt/-** | **nt/-** | **nt/-** | **nt/-** |
| BE14/14 | SS | nd | BF (Boulgou) | 2014 | **nt** | **nt/+** | **nt/-** | **nt/-** | **nt/-** | **nt/-** | **nt/-** | **nt/-** | **nt/-** |
| BE15/14 | SS | nd | BF (Boulgou) | 2014 | **nt** | **nt/+** | **nt/-** | **nt/-** | **nt/-** | **nt/-** | **nt/-** | **nt/-** | **nt/-** |
| BE16/14 | SS | nd | BF (Boulgou) | 2014 | **nt** | **nt/nt** | **nt/-** | **nt/-** | **nt/-** | **nt/-** | **nt/-** | **nt/-** | **nt/-** |
| BE17/14 | SS | nd | BF (Boulgou) | 2014 | **nt** | **nt/nt** | **nt/-** | **nt/-** | **nt/-** | **nt/-** | **nt/-** | **nt/-** | **nt/-** |
| BE18/14 | SS | nd | BF (Boulgou) | 2014 | **nt** | **nt/nt** | **nt/-** | **nt/-** | **nt/-** | **nt/-** | **nt/-** | **nt/-** | **nt/-** |
| BE19/14 | SS | nd | BF (Boulgou) | 2014 | **nt** | **nt/nt** | **nt/-** | **nt/-** | **nt/-** | **nt/-** | **nt/-** | **nt/-** | **nt/-** |
| BE20/14 | SS | nd | BF (Boulgou) | 2014 | **nt** | **nt/nt** | **nt/-** | **nt/-** | **nt/-** | **nt/-** | **nt/-** | **nt/-** | **nt/-** |
| BE21/14 | SS | nd | BF (Boulgou) | 2014 | **nt** | **nt/nt** | **nt/-** | **nt/-** | **nt/-** | **nt/-** | **nt/-** | **nt/-** | **nt/-** |
| BE22/14 | SS | nd | BF (Boulgou) | 2014 | **nt** | **nt/nt** | **nt/-** | **nt/-** | **nt/-** | **nt/-** | **nt/-** | **nt/-** | **nt/-** |
| BE23/14 | SS | nd | BF (Boulgou) | 2014 | **nt** | **nt/nt** | **nt/-** | **nt/-** | **nt/-** | **nt/-** | **nt/-** | **nt/-** | **nt/-** |
| BE24/14 | SS | nd | BF (Boulgou) | 2014 | **nt** | **nt/nt** | **nt/-** | **nt/-** | **nt/-** | **nt/-** | **nt/-** | **nt/-** | **nt/-** |
| BE25/14 | SS | nd | BF (Boulgou) | 2014 | **nt** | **nt/nt** | **nt/-** | **nt/-** | **nt/-** | **nt/-** | **nt/-** | **nt/-** | **nt/-** |
| BE26/14 | Sudan | nd | BF (Houet) | 2014 | **nt** | **nt/nt** | **nt/-** | **nt/-** | **nt/-** | **nt/-** | **nt/-** | **nt/-** | **nt/-** |
| BE27/14 | Sudan | nd | BF (Houet) | 2014 | **nt** | **nt/nt** | **nt/-** | **nt/-** | **nt/-** | **nt/-** | **nt/-** | **nt/-** | **nt/-** |
| BE28/14 | Sudan | nd | BF (Houet) | 2014 | **nt** | **nt/nt** | **nt/-** | **nt/+** | **nt/-** | **nt/-** | **nt/-** | **nt/-** | **nt/-** |
| BE29/14 | Sudan | nd | BF (Houet) | 2014 | **nt** | **nt/nt** | **nt/-** | **nt/-** | **nt/-** | **nt/-** | **nt/-** | **nt/-** | **nt/-** |
| BE30/14 | Sudan | nd | BF (Houet) | 2014 | **nt** | **nt/nt** | **nt/-** | **nt/-** | **nt/-** | **nt/-** | **nt/-** | **nt/-** | **nt/-** |
| BE31/14 | Sudan | nd | BF (Houet) | 2014 | **nt** | **nt/nt** | **nt/-** | **nt/-** | **nt/-** | **nt/-** | **nt/-** | **nt/-** | **nt/-** |
| BE32/14 | Sudan | nd | BF (Houet) | 2014 | **nt** | **nt/nt** | **nt/-** | **nt/-** | **nt/-** | **nt/-** | **nt/-** | **nt/-** | **nt/-** |
| BE33/14 | Sudan | nd | BF (Houet) | 2014 | **nt** | **nt/nt** | **nt/-** | **nt/-** | **nt/-** | **nt/-** | **nt/-** | **nt/-** | **nt/-** |
| BE34/14 | Sudan | nd | BF (Houet) | 2014 | **nt** | **nt/nt** | **nt/-** | **nt/-** | **nt/-** | **nt/-** | **nt/-** | **nt/-** | **nt/-** |
| BE35/14 | Sudan | nd | BF (Houet) | 2014 | **nt** | **nt/nt** | **nt/-** | **nt/-** | **nt/-** | **nt/-** | **nt/-** | **nt/-** | **nt/-** |
| BE36/14 | Sudan | nd | BF (Houet) | 2014 | **nt** | **nt/nt** | **nt/-** | **nt/-** | **nt/-** | **nt/-** | **nt/-** | **nt/-** | **nt/-** |
| BE37/14 | Sudan | nd | BF (Houet) | 2014 | **nt** | **nt/nt** | **nt/-** | **nt/-** | **nt/-** | **nt/-** | **nt/-** | **nt/-** | **nt/-** |
| BE38/14 | Sudan | nd | BF (Houet) | 2014 | **nt** | **nt/+** | **nt/-** | **nt/-** | **nt/-** | **nt/-** | **nt/-** | **nt/-** | **nt/-** |
| BE39/14 | Sudan | nd | BF (Houet) | 2014 | **nt** | **nt/nt** | **nt/-** | **nt/-** | **nt/-** | **nt/-** | **nt/-** | **nt/-** | **nt/-** |
| BE40/14 | Sudan | nd | BF (Houet) | 2014 | **nt** | **nt/+** | **nt/-** | **nt/-** | **nt/-** | **nt/-** | **nt/-** | **nt/-** | **nt/-** |
| BE41/14 | Sudan | nd | BF (Houet) | 2014 | **nt** | **nt/nt** | **nt/-** | **nt/-** | **nt/-** | **nt/-** | **nt/-** | **nt/-** | **nt/-** |
| BE42/14 | Sudan | nd | BF (Houet) | 2014 | **nt** | **nt/nt** | **nt/-** | **nt/-** | **nt/-** | **nt/-** | **nt/-** | **nt/-** | **nt/-** |
| BE43/14 | Sudan | nd | BF (Houet) | 2014 | **nt** | **nt/nt** | **nt/-** | **nt/-** | **nt/-** | **nt/-** | **nt/-** | **nt/-** | **nt/-** |
| BE44/14 | Sudan | nd | BF (Houet) | 2014 | **nt** | **nt/nt** | **nt/-** | **nt/-** | **nt/-** | **nt/-** | **nt/-** | **nt/-** | **nt/-** |
| BE45/14 | Sudan | nd | BF (Houet) | 2014 | **nt** | **nt/nt** | **nt/-** | **nt/-** | **nt/-** | **nt/-** | **nt/-** | **nt/-** | **nt/-** |
| BE46/14 | Sudan | nd | BF (Comoe) | 2014 | **nt** | **nt/nt** | **nt/-** | **nt/-** | **nt/-** | **nt/-** | **nt/-** | **nt/-** | **nt/-** |
| BE47/14 | Sudan | nd | BF (Comoe) | 2014 | **nt** | **nt/nt** | **nt/-** | **nt/-** | **nt/-** | **nt/-** | **nt/-** | **nt/-** | **nt/-** |
| BE48/14 | Sudan | nd | BF (Comoe) | 2014 | **nt** | **nt/nt** | **nt/-** | **nt/-** | **nt/-** | **nt/-** | **nt/-** | **nt/-** | **nt/-** |
| BE49/14 | Sudan | nd | BF (Comoe) | 2014 | **nt** | **nt/nt** | **nt/-** | **nt/-** | **nt/-** | **nt/-** | **nt/-** | **nt/-** | **nt/-** |
| BE50/14 | Sudan | nd | BF (Comoe) | 2014 | **nt** | **nt/+** | **nt/-** | **nt/-** | **nt/-** | **nt/-** | **nt/-** | **nt/-** | **nt/-** |
| BE51/14 | Sudan | nd | BF (Comoe) | 2014 | **nt** | **nt/nt** | **nt/-** | **nt/-** | **nt/-** | **nt/-** | **nt/-** | **nt/-** | **nt/-** |
| BE52/14 | Sudan | nd | BF (Comoe) | 2014 | **nt** | **nt/nt** | **nt/-** | **nt/-** | **nt/-** | **nt/-** | **nt/-** | **nt/-** | **nt/-** |
| BE53/14 | Sudan | nd | BF (Comoe) | 2014 | **nt** | **nt/nt** | **nt/-** | **nt/-** | **nt/-** | **nt/-** | **nt/-** | **nt/-** | **nt/-** |
| BE54/14 | Sudan | nd | BF (Comoe) | 2014 | **nt** | **nt/nt** | **nt/-** | **nt/-** | **nt/-** | **nt/-** | **nt/-** | **nt/-** | **nt/-** |
| BE55/14 | Sudan | nd | BF (Comoe) | 2014 | **nt** | **nt/nt** | **nt/-** | **nt/-** | **nt/-** | **nt/-** | **nt/-** | **nt/-** | **nt/-** |
| BE56/14 | Sudan | nd | BF (Comoe) | 2014 | **nt** | **nt/nt** | **nt/-** | **nt/-** | **nt/-** | **nt/-** | **nt/-** | **nt/-** | **nt/-** |
| BE57/14 | Sudan | nd | BF (Comoe) | 2014 | **nt** | **nt/nt** | **nt/-** | **nt/-** | **nt/-** | **nt/-** | **nt/-** | **nt/-** | **nt/-** |
| BE58/14 | Sudan | nd | BF (Comoe) | 2014 | **nt** | **nt/nt** | **nt/-** | **nt/-** | **nt/-** | **nt/-** | **nt/-** | **nt/-** | **nt/-** |
| BE59/14 | Sudan | nd | BF (Comoe) | 2014 | **nt** | **nt/nt** | **nt/-** | **nt/-** | **nt/-** | **nt/-** | **nt/-** | **nt/-** | **nt/-** |
| BE60/14 | Sudan | nd | BF (Comoe) | 2014 | **nt** | **nt/nt** | **nt/-** | **nt/-** | **nt/-** | **nt/-** | **nt/-** | **nt/-** | **nt/-** |
| BE61/14 | Sudan | nd | BF (Comoe) | 2014 | **nt** | **nt/nt** | **nt/-** | **nt/-** | **nt/-** | **nt/-** | **nt/-** | **nt/-** | **nt/-** |
| BE62/14 | Sudan | nd | BF (Comoe) | 2014 | **nt** | **nt/nt** | **nt/-** | **nt/-** | **nt/-** | **nt/-** | **nt/-** | **nt/-** | **nt/-** |
| BE63/14 | Sudan | nd | BF (Comoe) | 2014 | **nt** | **nt/+** | **nt/-** | **nt/-** | **nt/-** | **nt/-** | **nt/-** | **nt/-** | **nt/-** |
| BE64/14 | Sudan | nd | BF (Comoe) | 2014 | **nt** | **nt/nt** | **nt/-** | **nt/-** | **nt/-** | **nt/-** | **nt/-** | **nt/-** | **nt/-** |
| BE65/14 | Sudan | nd | BF (Comoe) | 2014 | **nt** | **nt/nt** | **nt/-** | **nt/-** | **nt/-** | **nt/-** | **nt/-** | **nt/-** | **nt/-** |
| BE66/14 | Sudan | nd | BF (Comoe) | 2014 | **nt** | **nt/nt** | **nt/-** | **nt/-** | **nt/-** | **nt/-** | **nt/-** | **nt/-** | **nt/-** |
| BE67/14 | Sudan | nd | BF (Comoe) | 2014 | **nt** | **nt/nt** | **nt/-** | **nt/-** | **nt/-** | **nt/-** | **nt/-** | **nt/-** | **nt/-** |
| BE68/14 | Sudan | nd | BF (Comoe) | 2014 | **nt** | **nt/nt** | **nt/-** | **nt/-** | **nt/-** | **nt/-** | **nt/-** | **nt/-** | **nt/-** |
| BE69/14 | Sudan | nd | BF (Comoe) | 2014 | **nt** | **nt/nt** | **nt/-** | **nt/-** | **nt/-** | **nt/-** | **nt/-** | **nt/-** | **nt/-** |
| BE70/14 | Sudan | nd | BF (Comoe) | 2014 | **nt** | **nt/nt** | **nt/+** | **nt/-** | **nt/-** | **nt/-** | **nt/-** | **nt/-** | **nt/-** |
| BE71/14 | Sudan | nd | BF (Poni) | 2014 | **nt** | **nt/nt** | **nt/-** | **nt/-** | **nt/-** | **nt/-** | **nt/-** | **nt/-** | **nt/-** |
| BE72/14 | Sudan | nd | BF (Poni) | 2014 | **nt** | **nt/nt** | **nt/-** | **nt/-** | **nt/-** | **nt/-** | **nt/-** | **nt/-** | **nt/-** |
| BE73/14 | Sudan | nd | BF (Poni) | 2014 | **nt** | **nt/nt** | **nt/-** | **nt/-** | **nt/-** | **nt/-** | **nt/-** | **nt/-** | **nt/-** |
| BE74/14 | Sudan | nd | BF (Poni) | 2014 | **nt** | **nt/nt** | **nt/-** | **nt/-** | **nt/-** | **nt/-** | **nt/-** | **nt/-** | **nt/-** |
| BE75/14 | Sudan | nd | BF (Poni) | 2014 | **nt** | **nt/nt** | **nt/-** | **nt/-** | **nt/-** | **nt/-** | **nt/-** | **nt/-** | **nt/-** |
| BE76/14 | Sudan | nd | BF (Poni) | 2014 | **nt** | **nt/nt** | **nt/-** | **nt/-** | **nt/-** | **nt/-** | **nt/-** | **nt/-** | **nt/-** |
| BE77/14 | Sudan | nd | BF (Poni) | 2014 | **nt** | **nt/nt** | **nt/-** | **nt/-** | **nt/-** | **nt/-** | **nt/-** | **nt/-** | **nt/-** |
| BE78/14 | Sudan | nd | BF (Poni) | 2014 | **nt** | **nt/nt** | **nt/-** | **nt/-** | **nt/-** | **nt/-** | **nt/-** | **nt/-** | **nt/-** |
| BE79/14 | Sudan | nd | BF (Poni) | 2014 | **nt** | **nt/nt** | **nt/-** | **nt/-** | **nt/-** | **nt/-** | **nt/-** | **nt/-** | **nt/-** |
| BE80/14 | Sudan | nd | BF (Poni) | 2014 | **nt** | **nt/nt** | **nt/-** | **nt/-** | **nt/-** | **nt/-** | **nt/-** | **nt/-** | **nt/-** |
| BE81/14 | Sudan | nd | BF (Poni) | 2014 | **nt** | **nt/nt** | **nt/-** | **nt/-** | **nt/-** | **nt/-** | **nt/-** | **nt/-** | **nt/-** |
| BE82/14 | Sudan | nd | BF (Poni) | 2014 | **nt** | **nt/nt** | **nt/-** | **nt/-** | **nt/-** | **nt/-** | **nt/-** | **nt/-** | **nt/-** |
| BE83/14 | Sudan | nd | BF (Poni) | 2014 | **nt** | **nt/nt** | **nt/-** | **nt/-** | **nt/-** | **nt/-** | **nt/-** | **nt/-** | **nt/-** |
| BE84/14 | Sudan | nd | BF (Poni) | 2014 | **nt** | **nt/nt** | **nt/-** | **nt/-** | **nt/-** | **nt/-** | **nt/-** | **nt/-** | **nt/-** |
| BE85/14 | Sudan | nd | BF (Poni) | 2014 | **nt** | **nt/+** | **nt/-** | **nt/-** | **nt/-** | **nt/-** | **nt/-** | **nt/-** | **nt/-** |
| BE86/14 | Sudan | nd | BF (Poni) | 2014 | **nt** | **nt/nt** | **nt/-** | **nt/-** | **nt/-** | **nt/-** | **nt/-** | **nt/-** | **nt/-** |
| BE87/14 | Sudan | nd | BF (Poni) | 2014 | **nt** | **nt/nt** | **nt/-** | **nt/-** | **nt/-** | **nt/-** | **nt/-** | **nt/-** | **nt/-** |
| BE88/14 | Sudan | nd | BF (Poni) | 2014 | **nt** | **nt/nt** | **nt/-** | **nt/-** | **nt/-** | **nt/-** | **nt/-** | **nt/-** | **nt/-** |
| BE89/14 | Sudan | nd | BF (Poni) | 2014 | **nt** | **nt/nt** | **nt/-** | **nt/-** | **nt/-** | **nt/-** | **nt/-** | **nt/-** | **nt/-** |
| BE90/14 | Sudan | nd | BF (Poni) | 2014 | **nt** | **nt/nt** | **nt/-** | **nt/-** | **nt/-** | **nt/-** | **nt/-** | **nt/-** | **nt/-** |
| BE91/14 | Sudan | nd | BF (Poni) | 2014 | **nt** | **nt/nt** | **nt/-** | **nt/+** | **nt/-** | **nt/-** | **nt/-** | **nt/-** | **nt/-** |
| BE92/14 | Sudan | nd | BF (Poni) | 2014 | **nt** | **nt/nt** | **nt/-** | **nt/-** | **nt/-** | **nt/-** | **nt/-** | **nt/-** | **nt/-** |
| BE93/14 | Sudan | nd | BF (Poni) | 2014 | **nt** | **nt/nt** | **nt/-** | **nt/+** | **nt/-** | **nt/-** | **nt/-** | **nt/-** | **nt/+** |
| BE94/14 | Sudan | nd | BF (Poni) | 2014 | **nt** | **nt/nt** | **nt/-** | **nt/-** | **nt/-** | **nt/-** | **nt/-** | **nt/-** | **nt/-** |
| BE95/14 | Sudan | nd | BF (Poni) | 2014 | **nt** | **nt/nt** | **nt/-** | **nt/-** | **nt/-** | **nt/-** | **nt/-** | **nt/-** | **nt/-** |
| BE96/14 | Sudan | nd | BF (Poni) | 2014 | **nt** | **nt/nt** | **nt/-** | **nt/-** | **nt/-** | **nt/-** | **nt/-** | **nt/-** | **nt/-** |
| BE97/14 | Sudan | nd | BF (Poni) | 2014 | **nt** | **nt/nt** | **nt/-** | **nt/-** | **nt/-** | **nt/-** | **nt/-** | **nt/-** | **nt/-** |
| BE98/14 | Sudan | nd | BF (Poni) | 2014 | **nt** | **nt/nt** | **nt/-** | **nt/-** | **nt/-** | **nt/-** | **nt/-** | **nt/-** | **nt/-** |
| BE99/14 | Sudan | nd | BF (Poni) | 2014 | **nt** | **nt/nt** | **nt/-** | **nt/-** | **nt/-** | **nt/-** | **nt/-** | **nt/-** | **nt/-** |
| BE100/14 | Sudan | nd | BF (Poni) | 2014 | **nt** | **nt/+** | **nt/-** | **nt/-** | **nt/-** | **nt/-** | **nt/-** | **nt/-** | **nt/-** |
| BE101/14 | Sudan | nd | BF (Poni) | 2014 | **nt** | **nt/nt** | **nt/-** | **nt/-** | **nt/-** | **nt/-** | **nt/-** | **nt/-** | **nt/-** |
| BE102/14 | Sudan | nd | BF (Poni) | 2014 | **nt** | **nt/nt** | **nt/-** | **nt/-** | **nt/-** | **nt/-** | **nt/-** | **nt/-** | **nt/-** |
| BE103/14 | Sudan | nd | BF (Poni) | 2014 | **nt** | **nt/nt** | **nt/-** | **nt/-** | **nt/-** | **nt/-** | **nt/-** | **nt/-** | **nt/-** |

SS: Sudan-Sahel zone; BF: Burkina Faso; nd: not determined; nt: not tested.
