# Supplementary material for: Metagenomic-Based Screening and Molecular Characterization of Cowpea-Infecting Viruses in Burkina Faso
Source: PLoS One. 2016 Oct 20;11(10):e0165188. doi: 10.1371/journal.pone.0165188 (PMC5072566; doi:10.1371/journal.pone.0165188)
Supplement: S2 Table — (DOC) [file pone.0165188.s005.doc]

**Supplementary Table 2.** 10-nucleotide multiplex identifier (MID) tagged DNA primers used for PCR from cDNA

Number Name Sequence (5’ 3’)

1 MID-003 **AGACGCACT**CCCTTCGGATCCTCC

2 MID-004 **AGCACTGTAG**CCTTCGGATCCTCC

3 MID-005 **ATCAGACACG**CCTTCGGATCCTCC

4 MID-006 **ATATCGCGAG**CCTTCGGATCCTCC

5 MID-007 **CGTGTCTCTA**CCTTCGGATCCTCC

6 MID-013 **CATAGTAGTG**CCTTCGGATCCTCC

7 MID-014 **CGAGAGATAC**CCTTCGGATCCTCC

8 MID-015 **ATACGACGTA**CCTTCGGATCCTCC

9 MID-017 **CGTCTAGTAC**CCTTCGGATCCTCC

10 MID-020 **ACGACTACAG**CCTTCGGATCCTCC

11 MID-021 **CGTAGACTAG**CCTTCGGATCCTCC

12 MID-026 **ACATACGCGT**CCTTCGGATCCTCC

13 MID-027 **ACGCGAGTAT**CCTTCGGATCCTCC

14 MID-035 **CAGTAGACGT**CCTTCGGATCCTCC

15 MID-049 **ACGCGATCGA**CCTTCGGATCCTCC

16 MID-051 **AGCTCACGTA**CCTTCGGATCCTCC

17 MID-053 **AGTCGAGAGA**CCTTCGGATCCTCC

18 MID-054 **AGTGCTACGA**CCTTCGGATCCTCC

19 MID-056 **CGCAGTACGA**CCTTCGGATCCTCC

20 MID-057 **CGCGTATACA**CCTTCGGATCCTCC

21 MID-058 **CGTACAGTCA**CCTTCGGATCCTCC

22 MID-059 **CGTACTCAGA**CCTTCGGATCCTCC

23 MID-060 **CTACGCTCTA**CCTTCGGATCCTCC

24 MID-061 **CTATAGCGTA**CCTTCGGATCCTCC

25 MID-074 **ACACATACGC**CCTTCGGATCCTCC

26 MID-075 **ACAGTCGTGC**CCTTCGGATCCTCC

27 MID-076 **ACATGACGAC**CCTTCGGATCCTCC

28 MID-077 **ACGACAGCTC**CCTTCGGATCCTCC

29 MID-078 **ACGTCTCATC**CCTTCGGATCCTCC

30 MID-079 **ACTCATCTAC**CCTTCGGATCCTCC

31 MID-083 **AGTAGTGATC**CCTTCGGATCCTCC

32 MID-084 **AGTGACACAC**CCTTCGGATCCTCC

33 MID-087 **ATATAGTCGC**CCTTCGGATCCTCC

34 MID-088 **ATCTACTGAC**CCTTCGGATCCTCC

35 MID-089 **CACGTAGATC**CCTTCGGATCCTCC

36 MID-091 **CATACTCTAC**CCTTCGGATCCTCC

37 MID-092 **CGACACTATC**CCTTCGGATCCTCC

38 MID-094 **CGTATGCGAC**CCTTCGGATCCTCC

39 MID-095 **CGTCGATCTC**CCTTCGGATCCTCC

40 MID-096 **CTACGACTGC**CCTTCGGATCCTCC

41 MID-097 **CTAGTCACTC**CCTTCGGATCCTCC

42 MID-098 **CTCTACGCTC**CCTTCGGATCCTCC

43 MID-115 **ACTCACAGAG**CCTTCGGATCCTCC

44 MID-116 **AGACTCAGCG**CCTTCGGATCCTCC

45 MID-117 **AGAGAGTGTG**CCTTCGGATCCTCC

46 MID-118 **AGCTATCGCG**CCTTCGGATCCTCC

47 MID-119 **AGTCTGACTG**CCTTCGGATCCTCC

48 MID-120 **AGTGAGCTCG**CCTTCGGATCCTCC

49 MID-121 **ATAGCTCTCG**CCTTCGGATCCTCC

50 MID-123 **ATCGTAGCAG**CCTTCGGATCCTCC

51 MID-125 **ATGTACGATG**CCTTCGGATCCTCC

52 MID-126 **ATGTGTCTAG**CCTTCGGATCCTCC

53 MID-137 **CTCGATATAG**CCTTCGGATCCTCC

54 MID-8 **CTCGCGTGTC**CCTTCGGATCCTCC

55 MID-28 **ACTACTATGT**CCTTCGGATCCTCC

56 MID-33 **ATAGAGTACT**CCTTCGGATCCTCC

57 MID-48 **ACAGTATATA**CCTTCGGATCCTCC

58 MID-52 **AGTATACATA**CCTTCGGATCCTCC

59 MID-80 **ACTCGCGCAC**CCTTCGGATCCTCC

60 MID-86 **ATAGATAGAC**CCTTCGGATCCTCC

61 MID-90 **CACGTGTCGC**CCTTCGGATCCTCC

62 MID-93 **CGAGACGCGC**CCTTCGGATCCTCC

63 MID-114 **ACGTGCAGCG**CCTTCGGATCCTCC

64 MID-128 **CACTCGCACG**CCTTCGGATCCTCC

65 MID-131 **CGACAGCGAG**CCTTCGGATCCTCC

66 MID-134 **CGCTCGAGTG**CCTTCGGATCCTCC

67 MID-138 **CTCGCACGCG**CCTTCGGATCCTCC

68 MID-139 **CTGCGTCACG**CCTTCGGATCCTCC

69 MID-140 **CTGTGCGTCG**CCTTCGGATCCTCC

70 MID-2 **ACGCTCGACA**CCTTCGGATCCTCC

71 MID-18 **TCTACGTAGC**CCTTCGGATCCTCC

72 MID-34 **CACGCTACGT**CCTTCGGATCCTCC

73 MID-36 **CGACGTGACT**CCTTCGGATCCTCC

74 MID-37 **TACACACACT**CCTTCGGATCCTCC

75 MID-47 **TGTGAGTAGT**CCTTCGGATCCTCC

76 MID-64 **TATATATACA**CCTTCGGATCCTCC

77 MID-65 **TATGCTAGTA**CCTTCGGATCCTCC

78 MID-68 **TCGCTGCGTA**CCTTCGGATCCTCC

79 MID-73 **TGTCGTCGCA**CCTTCGGATCCTCC

80 MID-85 **AGTGTATGTC**CCTTCGGATCCTCC

81 MID-101 **TAGCGCGCGC**CCTTCGGATCCTCC

82 MID-102 **TAGCTCTATC**CCTTCGGATCCTCC

83 MID-103 **TATAGACATC**CCTTCGGATCCTCC

84 MID-106 **TCATCGAGTC**CCTTCGGATCCTCC

85 MID-107 **TCGAGCTCTC**CCTTCGGATCCTCC

86 MID-108 **TCGCAGACAC**CCTTCGGATCCTCC

87 MID-109 **TCTGTCTCGC**CCTTCGGATCCTCC

88 MID-122 **ATCACGTGCG**CCTTCGGATCCTCC

89 MID-124 **ATCGTCTGTG**CCTTCGGATCCTCC

90 MID-132 **CGATCTGTCG**CCTTCGGATCCTCC

91 MID-133 **CGCGTGCTAG**CCTTCGGATCCTCC

92 MID-136 **CTATGTACAG**CCTTCGGATCCTCC

93 MID-149 **TGATAGAGCG**CCTTCGGATCCTCC

94 MID-151 **TGCTAGTCAG**CCTTCGGATCCTCC

95 MID-152 **TGTATCACAG**CCTTCGGATCCTCC

96 MID-153 **TGTGCGCGTG**CCTTCGGATCCTCC
