# Supplementary material for: Metagenomic-Based Screening and Molecular Characterization of Cowpea-Infecting Viruses in Burkina Faso
Source: PLoS One. 2016 Oct 20;11(10):e0165188. doi: 10.1371/journal.pone.0165188 (PMC5072566; doi:10.1371/journal.pone.0165188)
Supplement: S3 Table — (DOCX) [file pone.0165188.s006.docx]

**Supplementary table 3:** List of NCBI GenBank sequences used for designing primers

| **Cowpea virus** | **NCBI accession number** | **Organism (NCBI)** |
| --- | --- | --- |
| CPMoV | NC_003535 | Cowpea mottle virus |
|  | FJ707484 | Soybean yellow mottle mosaic virus strain MS1 |
|  | FJ457015 | Soybean yellow mottle mosaic virus from South Korea |
|  | AB250686 | Melon necrotic spot virus strain: Nagasaki |
| SCPMV | NC_001625 | Southern cowpea mosaic virus |
|  | NC_016033 | Soybean yellow common mosaic virus |
|  | NC_004060 | Southern bean mosaic virus isolate Sao Paulo |
|  | AF055888 | Southern bean mosaic virus strain SBMV-S |
|  | AF055887 | Southern bean mosaic virus strain SBMV-B |
|  | NC_002568 | Sesbania mosaic virus |
| Cowpea polerovirus 1  Cowpea polerovirus 2 | AY956384 | Chickpea chlorotic stunt virus isolate Et-fb-am1 |
|  | HM804472 | Beet western yellows virus strain BJ genotype B |
|  | EU636992 | Cucurbit aphid-borne yellows virus from China |
|  | NC_010809 | Melon aphid-borne yellows virus |
|  | NC_018571 | Suakwa aphid-borne yellows virus |
| Cowpea associated mycotymovirid 1 | KT360947 | Fusarium graminearum mycotymovirus 1 isolate SX64 |
|  | NC_002164 | Poinsettia mosaic virus |
|  | U87832 | Oat blue dwarf virus |
| Cowpea tombusvirid 1 | NC_003633 | Oat chlorotic stunt virus |
|  | NC_003627 | Maize chlorotic mottle virus |
| Cowpea tombusvirid 2 | NC_009533 | Maize white line mosaic virus |
| Cowpea tombusvirid 3 | NC_014967 | Honeysuckle ringspot virus |
