# Supplementary material for: Metagenomic-Based Screening and Molecular Characterization of Cowpea-Infecting Viruses in Burkina Faso
Source: PLoS One. 2016 Oct 20;11(10):e0165188. doi: 10.1371/journal.pone.0165188 (PMC5072566; doi:10.1371/journal.pone.0165188)
Supplement: S4 Table — (DOCX) [file pone.0165188.s007.docx]

**Supplementary table 4:** List of primers used for Cowpea polerovirus 1 and Cowpea polerovirus 2 partial genome amplification

| **Virus** | **Primers** | **Sequence** | **Position** | **Gene** | **Ta °C** | **Amplicon**  **length (bp)** |
| --- | --- | --- | --- | --- | --- | --- |
| Cowpea polerovirus 1 | PoleroNB1F | CAAAAGAATTCAGGAGGGACTA | 1 | P0 | 55 | 161 |
|  | PoleroNB162R | AGTCTTTGTGGGAGGCTTTCGTC | 162 |  | 55 |  |
|  | PoleroNB1F | CAAAAGAATTCAGGAGGGACTA | 1 | P0 | 55 | 201 |
|  | PoleroNB202R | CTGGCACAGATGGTAGAGGAGG | 202 |  | 55 |  |
|  | PoleroNB1F | CAAAAGAATTCAGGAGGGACTA | 1 | P0 | 55 | 989 |
|  | PoleroNB990R | GCCGTGACCAAAGCATTGG | 990 |  | 55 |  |
|  | PoleroNB527F | GCGAGACTTTCAACAAGACG | 527 | P1 | 55 | 1008 |
|  | PoleroNB1535R | GACCAAACCTCTTTACCCTTG | 1535 |  | 55 |  |
|  | PoleroNB990F | CCAATGCTTTGGTCACGGC | 990 | P1 | 55 | 1012 |
|  | PoleroNB2002R | GGGTGTTTCCTGACGAGCT | 2002 |  | 55 |  |
|  | PoleroNB1535F | CAAGGGTAAAGAGGTTTGGTC | 1535 | P2 | 55 | 1036 |
|  | PoleroNB2571R | CGAGTTGGTCCACAAGTGAC | 2571 |  | 55 |  |
|  | PoleroNB2002F | AGCTCGTCAGGAAACACCC | 2002 | P2 | 55 | 1091 |
|  | PoleroNB3093R | CTCGACTTTGAGACCGAGC | 3093 |  | 55 |  |
|  | PoleroNB2571F | GTCACTTGTGGACCAACTCG | 2571 | P2/P3 | 55 | 1029 |
|  | PoleroNB3600R | ACGGTTAGGTCCTCCATTTC | 3600 |  | 55 |  |
|  | PoleroNB3093F | GCTCGGTCTCAAAGTCGAG | 3093 | P3 | 55 | 939 |
|  | PoleroNB4032R | GGTGTCATGCCACTCAACC | 4032 |  | 55 |  |
|  | PoleroNB3600F | GAAATGGAGGACCTAACCGT | 3600 | P3/P5 | 55 | 861 |
|  | PoleroNB4461R | CCATCATGCCATTACATTCCA | 4461 |  | 55 |  |
|  | PoleroNB4032F | GGTTGAGTGGCATGACACC | 4032 | P3/P5 | 55 | 944 |
|  | PoleroNB4976R | GGTCCATGACCTGTTTTCCT | 4976 |  | 55 |  |
| Cowpea polerovirus 2 | Polero2NB1637F | GCAGAACAACCGGAAAATCC | 1637 | P1 | 55 | 805 |
|  | Polero2NB2442R | GGATCGGGTCACAGAGACC | 2442 |  | 55 |  |
|  | Polero2NB1637F | GCAGAACAACCGGAAAATCC | 1637 | P1/P2 | 55 | 1390 |
|  | Polero2NB3027R | CTGATTCTAGGGCGTCATCG | 3027 |  | 55 |  |
|  | Polero2NB2423F | GGTCTCTGTGACCCGATCC | 2423 | P0 | 55 | 604 |
|  | Polero2NB3027R | CTGATTCTAGGGCGTCATCG | 3027 |  | 55 |  |
|  | Polero2NB2423F | GGTCTCTGTGACCCGATCC | 2423 | P2 | 55 | 1089 |
|  | Polero2NB3512R | CAATTGATCGAACGTGGGCA | 3512 |  | 55 |  |
|  | Polero2NB3008F | CGATGACGCCCTAGAATCAG | 3008 | P2 | 55 | 504 |
|  | Polero2NB3512R | CAATTGATCGAACGTGGGCA | 3512 |  | 55 |  |
|  | Polero2NB3008F | CGATGACGCCCTAGAATCAG | 3008 | P2/P3 | 55 | 1022 |
|  | Polero2NB4030R | CGGAATTGATCTTCGGTCGC | 4030 |  | 55 |  |
|  | Polero2NB3493F | TGCCCACGTTCGATCAATTG | 3493 | P3 | 55 | 537 |
|  | Polero2NB4030R | CGGAATTGATCTTCGGTCGC | 4030 |  | 55 |  |
|  | Polero2NB3493F | TGCCCACGTTCGATCAATTG | 3493 | P3/P5 | 55 | 1043 |
|  | Polero2NB4536R | CCTTCCAGTTGCCTATATTCC | 4536 |  | 55 |  |
|  | Polero2NB4011F | GCGACCGAAGATCAATTCCG | 4011 | P3/P5 | 55 | 525 |
|  | Polero2NB4536R | CCTTCCAGTTGCCTATATTCC | 4536 |  | 55 |  |
|  | Polero2NB4011F | GCGACCGAAGATCAATTCCG | 4011 | P5 | 55 | 768 |
|  | Polero2NB4779R | CCCATTCACACATGTTTTCTG | 4779 |  | 55 |  |
|  | Polero2NB4516F | GGAATATAGGCAACTGGAAGG | 4516 | P5 | 55 | 263 |
|  | Polero2NB4779R | CCCATTCACACATGTTTTCTG | 4779 |  | 55 |  |
